# Bacteria-host transcriptional response during endothelial invasion by *Staphylococcus aureus*

**DOI:** 10.1038/s41598-021-84050-x

**Published:** 2021-03-16

**Authors:** Rasmus Birkholm Grønnemose, Christian Garde, Claes Søndergaard Wassmann, Janne Kudsk Klitgaard, Ronni Nielsen, Susanne Mandrup, Andreas Holm Mattsson, Thomas Emil Andersen

**Affiliations:** 1 Research Unit of Clinical Microbiology, University of Southern Denmark and Odense University Hospital, J.B. Winsløws Vej 21.2, 5000 Odense, Denmark; 2 Evaxion Biotech A/S, Copenhagen, Denmark; 3 Research Unit of Molecular Microbiology, Department of Biochemistry and Molecular Biology, University of Southern Denmark, Odense, Denmark; 4 Functional Genomics and Metabolism Research Unit, Department of Biochemistry and Molecular Biology, University of Southern Denmark, Odense, Denmark

**Keywords:** Cellular microbiology, Bacterial host response, Bacterial transcription, Pathogens

## Abstract

*Staphylococcus aureus* is the cause of serious vascular infections such as sepsis and endocarditis. These infections are notoriously difficult to treat, and it is believed that the ability of *S. aureus* to invade endothelial cells and persist intracellularly is a key mechanism for persistence despite ongoing antibiotic treatment. Here, we used dual RNA sequencing to study the simultaneous transcriptional response of *S. aureus* and human endothelial cells during in vitro infections. We revealed discrete and shared differentially expressed genes for both host and pathogen at the different stages of infection. While the endothelial cells upregulated genes involved in interferon signalling and antigen presentation during late infection, *S. aureus* downregulated toxin expression while upregulating genes related to iron scavenging. In conclusion, the presented data provide an important resource to facilitate functional investigations into host–pathogen interaction during *S. aureus* invasive infection and a basis for identifying novel drug target sites.

## Introduction

*Staphylococcus aureus* is a major pathogen in human bloodstream infections. The bacterium is able to spread from an initial site of entry such as an indwelling central venous catheter to various organs including the lungs, bones, and heart valves. To establish itself on these sites, *S. aureus* expresses virulence factors involved in e.g. adhesion, immune evasion, and toxin production^1,2^. Historically, *S. aureus* has been viewed solely as an extracellular pathogen, but research conducted during the past decades has demonstrated a pronounced ability for this bacterium to invade and colonize both professional and non-professional phagocytes such as endothelial cells. This behaviour has since been linked to the pathogenesis of *S. aureus*, in particular its ability to spread via the blood to organs^3^ and its resilience against host response and antibiotic treatment e.g. in bloodstream infections such as endocarditis^4^. Though the role of cellular invasion has not been completely clarified, it is believed to entail immune or antibiotic escape leading to subsequent relapse of infection from the intracellular reservoir^4–6^. How the bacterium reemerges from the invaded endothelial cell or penetrates deeper into target tissues, however, remains to be revealed^6^.

Bacterial mutagenesis studies have shown which genes are utilized by the bacterium to facilitate endothelial adhesion, invasion, and intracellular survival^2,6–8^. To this date, however, a global genetic analysis is lacking which maps the expression of genes, essential as well as non-essential, for both the bacterium and the human cell simultaneously during invasive endothelial infection. This is important not only to understand the fundamental interactions between host and pathogen, but also from a treatment perspective, to identify new targets for intervention. To achieve such data, new research tools such as RNA-Seq have been employed, which quantify the global gene expression of an organism at a given sample timepoint. An expanded version of this technique is dual RNA-Seq, which has recently been employed to study the transcriptional response of both the host and the pathogen simultaneously hereby revealing new knowledge on disease pathogenesis and potential treatment targets^9,10^.

In this study, we used dual RNA-Seq to study the complex transcriptional response of primary human endothelial cells and *S. aureus* during in vitro invasive infection. Our results show both discrete and shared differentially expressed genes during the different stages of endothelial infection. Functional enrichment analysis furthermore revealed that during the late stage of the infection, an enrichment of endothelial genes involved in interferon signalling, antigen presentation, and apoptosis were upregulated, and on the other hand for *S. aureus*, genes for toxins and quorum sensing were downregulated whereas genes for iron scavenging were upregulated after infection. These data could indicate the importance of toxin repression and improved iron scavenging for intracellular survival of *S. aureus* during infection.

## Results

### Cell infection, sequence depth, and sequence mapping

**Figure 1:**
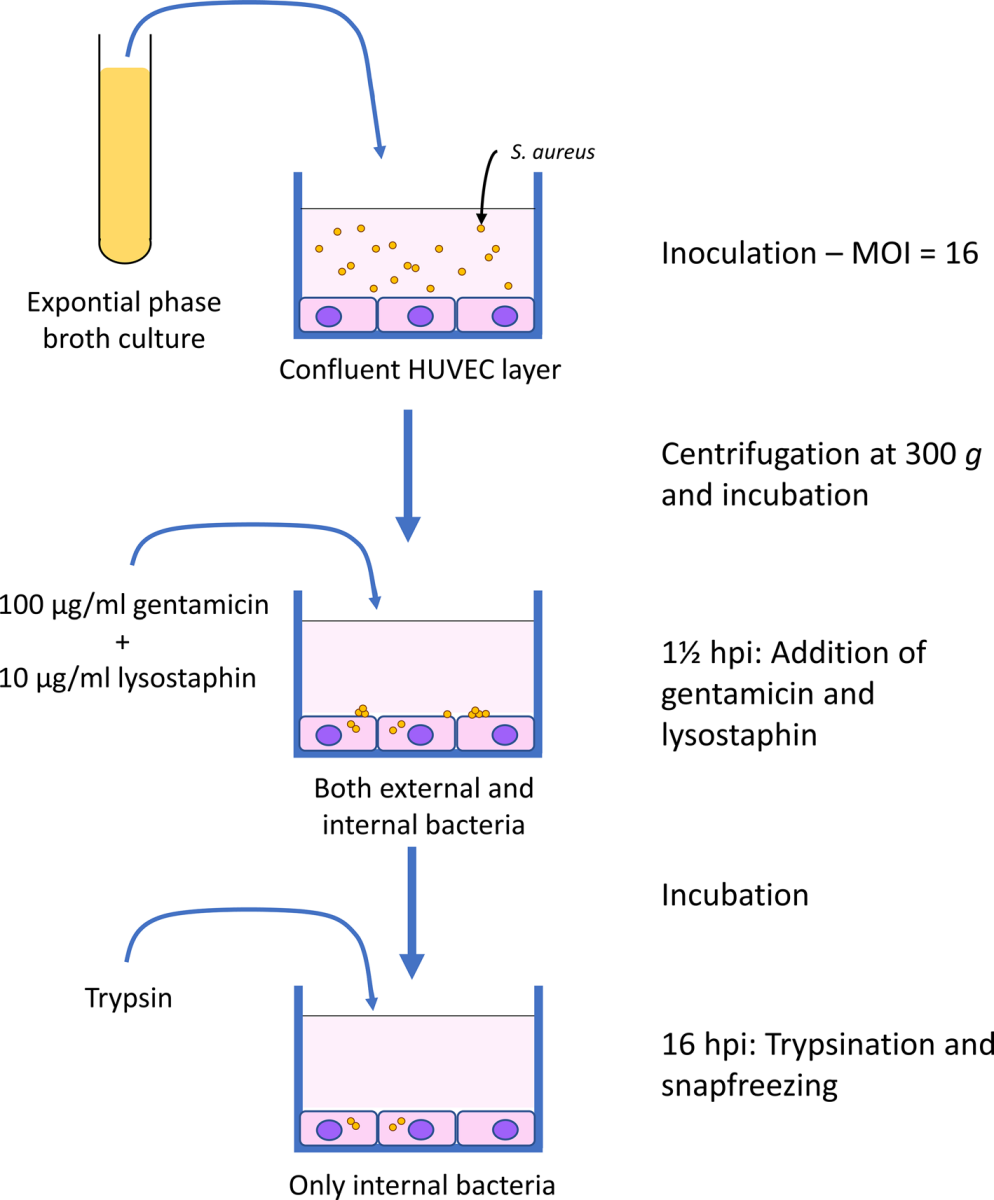
Flow chart of the infection assay. An exponential phase broth culture is prepared and used to inoculate microtiter plates containing confluent HUVEC layers using a multiplicity of infection of ∼ 16. After sedimentation of the bacteria by centrifugation at 300 *g*, the plates are incubated at 37 °C for 1½h. Then, 100 μg/ml gentamicin and 10 μg/ml lysostaphin is added to kill and lyse all extracellular bacteria. For sample collection at 1 hpi and 16 hpi, the plates are washed and trypsinized followed by snap freezing and storage at − 80 °C until RNA preparation.

To assess the bacteria-host transcriptional response during invasive endothelial infection, an infection protocol using human umbilical vein endothelial cells (HUVECs) and *S. aureus* ATCC strain 29213 was used as outlined in Fig. 1. Bacteria from an exponential phase broth culture was used at a multiplicity of infection (MOI) of 16 to infect the HUVECs, and bacteria-HUVEC contact synchronized by centrifugation.

After 1 h post infection (hpi), the first samples were collected by washing, trypsinization, and snap-freezing total material from the wells, containing HUVECs, external and invading bacteria. Examining the amount of internal vs. external bacteria at this time point by agar plating of untreated samples and samples treated with gentamicin and lysostaphin showed that around 67.0% ± 10.2% (mean ± SD, n = 4) of the bacteria appeared to be located internally compared to the untreated samples, which indicates that most of the bacteria are already internalized at 1 hpi.

At 1½ hpi onwards, the remaining wells were supplemented with lysostaphin and gentamicin to kill and lyse the extracellular bacteria to facilitate later harvest of intracellular bacteria alone. After 16 hpi, these final samples were collected by washing, trypsinization, and snap-freezing.

**Figure 2:**
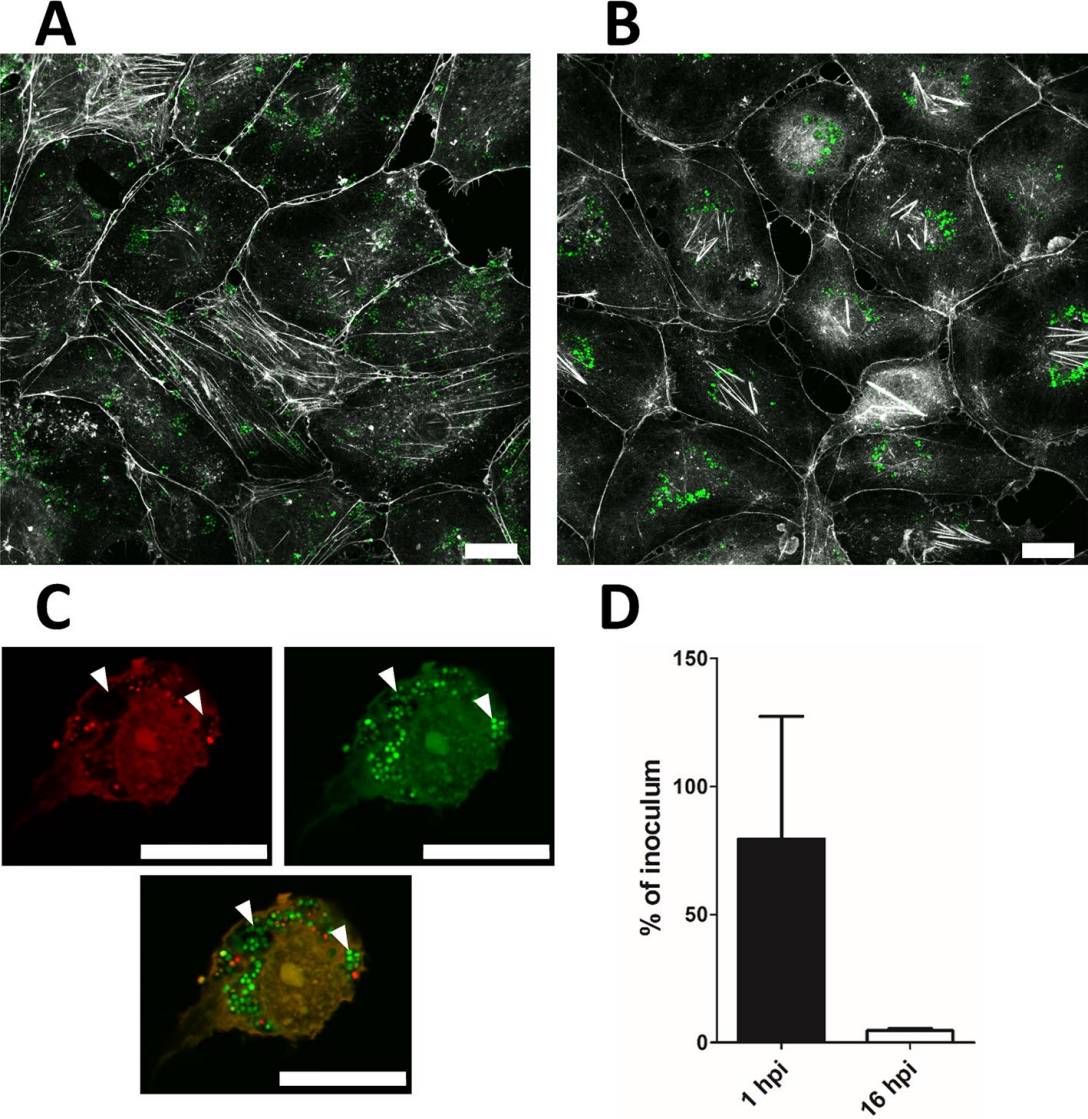
Infected HUVECs were visualized by confocal laser scanning microscopy (**A–C**) and quantified for bacterial numbers by plating (**D**). (**A–B**) GFP-producing *S. aureus* (green)have invaded the majority of the HUVECs (white, Acti-stain 670) at both 1 hpi (**A**) and 16 hpi (**B**). (**C**) At 16 hpi, bacteria are localised inside vacuolar compartments. Cells were stained with LIVE/DEAD cell viability stain (propidium iodide, red, and SYTO9, green). (**D**) Number of colony-forming units of *S. aureus* at 1 hpi and 16 hpi compared to the inoculum. Scale bars in (**A**–**C**) indicate 20 μm.

Bacterial infection/invasion efficiency was examined by confocal laser scanning microscopy (CLSM, Fig. 2A–C, Supplementary Fig. S1) and quantitatively assessed by agar plating (Fig. 2D). We experienced that agar plating yielded incongruent low numbers of colony forming units that did not agree well with the parallel microscopy examination of viability-stained bacterial specimens. This discrepancy most likely results in part from *S. aureus* aggregation and in part from the formation of dormant, non-dividing bacteria (small colony variants) in the intracellular state, both of which renders CFU enumeration on agar plates difficult and prone to underestimation^11,12^. From the CLSM analysis we found that the majority of the HUVEC were invaded at both 1 (Fig. 2A) and 16 hpi (Fig. 2B), with the bacteria appearing inside intracellular vacuoles (marked with white arrowheads in Fig. 2C and Supplementary Fig. S1). From this we assess that the RNA-Seq data generated from HUVECs reflect a genetic response almost exclusively from cells which are infected/invaded by *S. aureus*. Given that the infected human cells most likely contain much more human RNA than bacterial RNA (often more than 100-fold^13^), the number of bacterial reads that could be mapped from our data to the *S. aureus* ATCC 29213 genome from infected cultures were as expected relatively low (< 2 × 10^6^ reads) compared to the reads that could be mapped to the human hg38 genome (> 1 × 10^7^ reads), whereas the inoculum containing pure bacterial samples with approx. 6 × 10^7^ CFU resulted in a comparable number of reads (Supplementary Fig. S2).

### Overall transcriptional response during in vitro infection

**Figure 3:**
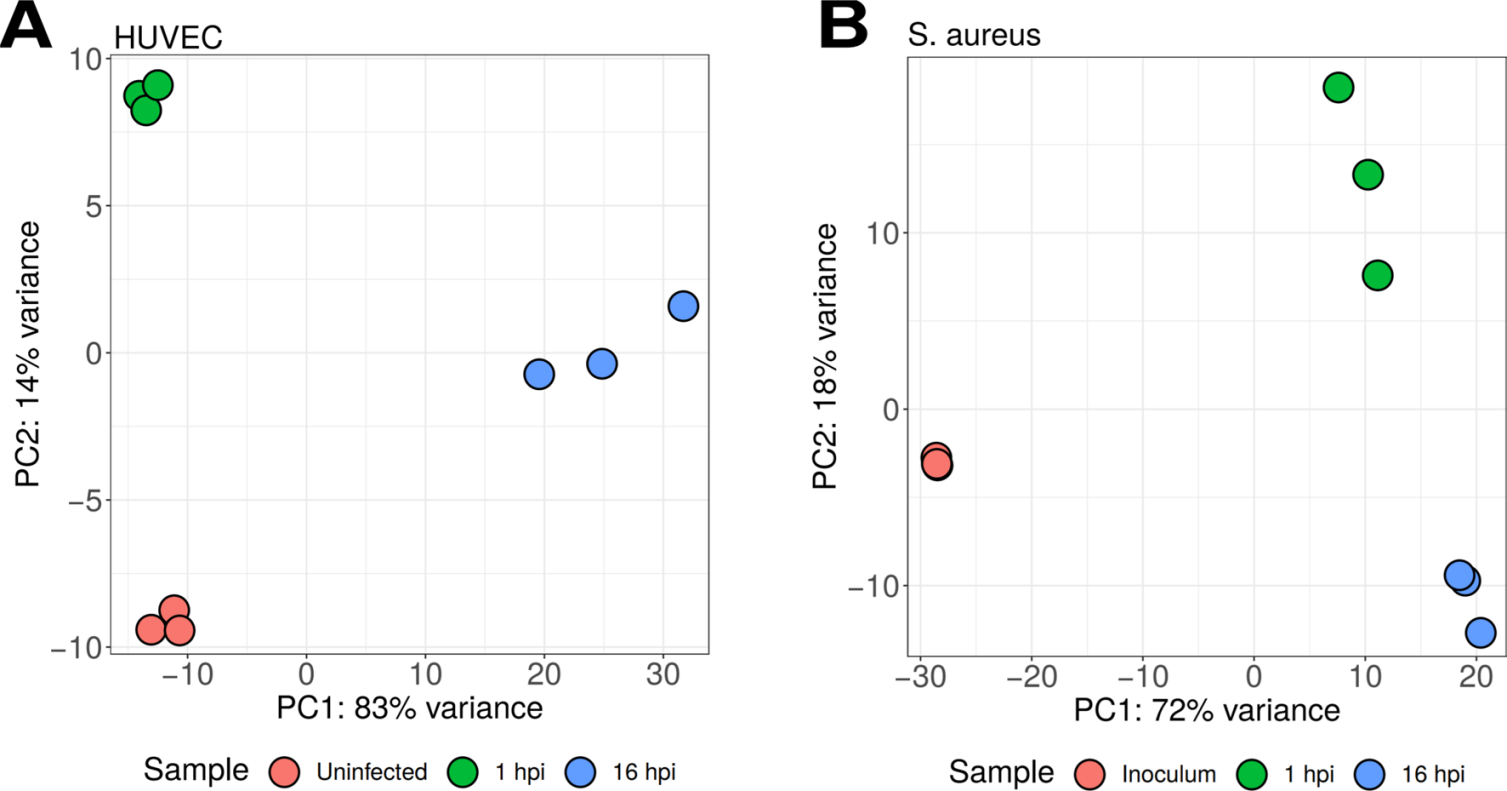
Principal component analysis (PCA) of HUVEC (**A**) and *S. aureus* (**B**) at different time-points during infection showing clustering into distinct expression profile groups corresponding to each infection time-point.

To assess the overall differences in gene expression during the 3 chosen time-points reflecting before infection, early stage infection, and late stage infection (uninfected, 1 hpi, and 16 hpi), we performed a dimensionality reduction using the principal component analysis (PCA). Here we saw a clear clustering of each triplicate into 3 distinct groups comprising each time-point for both HUVECs (Fig. 3A) and *S. aureus* (Fig. 3B) indicating a marked temporal gene regulation during infection.

To identify the shared differentially expressed genes between each timepoint, a pairwise comparison was conducted for both *S. aureus* and HUVECs using the DESeq2 workflow and the Wald test between 1 hpi and the inoculum/uninfected cells and between 1 and 16 hpi. For completeness, comparisons between the inoculum/uninfected cells and 16 hpi are included in the Supplementary Table S2. However, given that low expressed genes tend to have high coverage variability, we adjusted for false-positive differential genes by penalizing low coverage through logFC shrinkage^14^. Before logFC shrinkage, we identified 1460 and 577 differentially expressed genes for *S. aureus*, respectively, in the two time-point comparisons and 2362 and 3662 for HUVECs (Supplementary Fig. 3 and 4), respectively, at the two timepoint comparisons whereas after logFC shrinkage, this was reduced to 458 and 85, and 78 and 279, respectively (Supplementary Figs. 3 and 4).

### Differentially expressed genes for *S. aureus*

**Figure 4:**
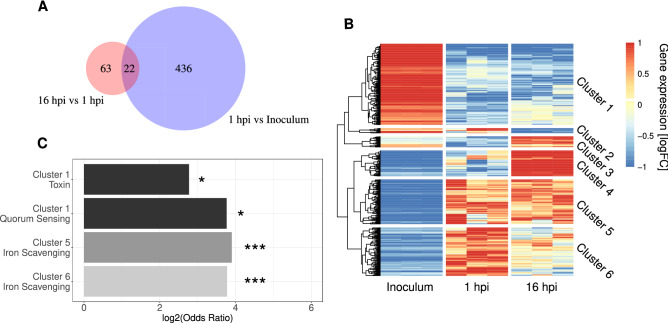
Analysis of differentially expressed genes for *S. aureus* between the inoculum, 1 hpi, and 16 hpi after logFC shrinkage. (**A**) Venn diagram visualizing the differentially expressed genes in the two comparisons with overlap showing the commonly regulated genes. (**B**) Heat map displaying the overall expression profile of all 521 differentially expressed genes grouped into 6 clusters indicating either up- or downregulation at specific time points during infection. Gene expression values were log2 transformed and hierarchically clustered according to Euclidean distances with the complete linkage approach. (**C**) Virulence ontology enrichment analysis of known *S. aureus* virulence factors using Fisher’s exact test. *p < 0.05, **p < 0.01, ***p < 0.001.

Looking at the overlap in differentially expressed genes for *S. aureus* between the two comparisons, 22 genes were commonly regulated (Fig. 4A), whereas 436 and 63 genes were differentially regulated between the two comparisons. The total 521 differentially expressed genes could furthermore be grouped into 6 discrete clusters (Cluster 1 to 6, Fig. 4B) that each represent a distinctive temporal expression profile. Cluster 1 to 3 consists of 196, 12, and 26 genes, respectively, that all have a significant upregulation of the grouped genes in the inoculum, but then takes 3 different expression routes. The largest cluster, cluster 1, follows an initial high expression of the assigned genes with a downregulation at both 1 and 16 hpi. In cluster 2, we see a continuous upregulation as in the inoculum at 1 hpi followed by a downregulation, and vice versa in cluster 3.

Cluster 4 to 6 consists of 62, 108, and 117 genes, respectively, that initially have a low expression, but then diverges into three regulation pathways. Cluster 4 is only upregulated at 16 hpi, whereas cluster 5 and 6 are upregulated at 1 hpi, but then diverge leading to either a continuous upregulation or a downregulation, respectively.

To identify if known virulence factors could be mapped to specific temporal expression clusters, a virulence ontology enrichment analysis was performed using a predefined list of known virulence factors (Supplementary Table S1). In cluster 1, a significant enrichment of genes related to toxins (i.e. the genes for γ-hemolysin; *hlgA*, *hlgB*, *hlgC*) and quorum sensing (*agrA*, *agrC*) was identified, whereas an enrichment of iron scavenging genes (*isdA*, *isdB*, *isdC*, *isdD*, *isdE, isdF*, *isdG*, *srtB*) could be observed in cluster 5 and 6 (Fig. 4C, Supplementary Fig. S5). This indicates that toxin and quorum sensing genes are downregulated post inoculation, whereas iron scavenging genes are upregulated post inoculation.

### Differentially expressed genes for HUVECs

**Figure 5:**
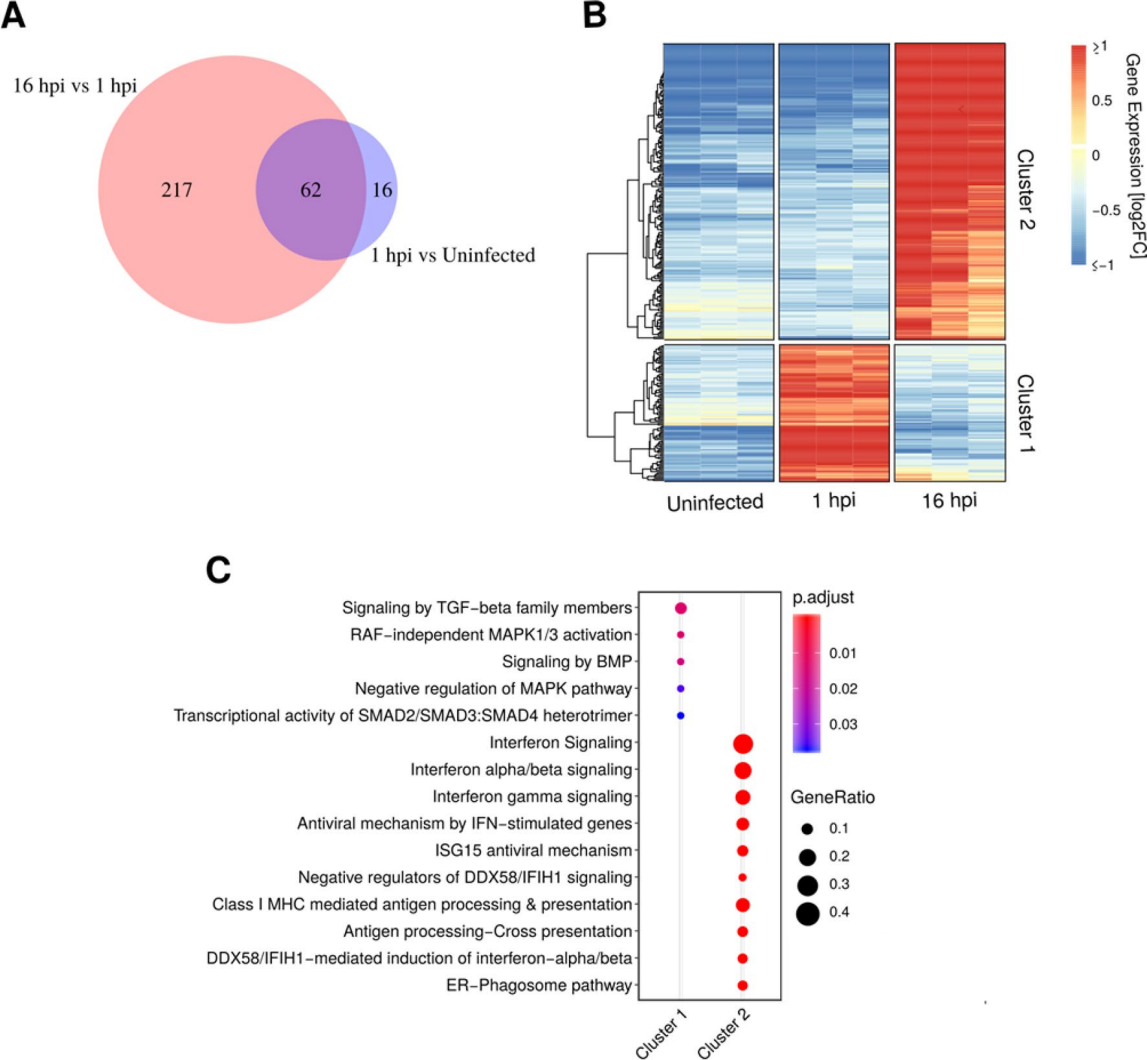
Analysis of differentially expressed genes for HUVEC between the uninfected, 1 hpi, and 16 hpi after logFC shrinkage. (**A**) Venn diagram visualizing the differentially expressed genes in the two comparisons with overlap showing the commonly regulated genes. (**B**) Heat map displaying the overall expression profile of all 295 differentially expressed genes grouped into 2 clusters indicating either up- or downregulation at specific time points during infection. Gene expression values have been log2 transformed and hierarchically clustered according to Euclidean distances with complete linkage. (**C**) Functional enrichment analysis of HUVEC genes that have been mapped to the Reactome Pathway database.

For HUVECs, an overlap of differentially expressed genes was also evident with 62 genes commonly regulated between the two comparisons (Fig. 5A), whereas 217 and 16 genes were differentially regulated in each of the two comparisons, respectively. The differentially expressed genes could be further grouped into 2 distinctive temporal expression clusters with cluster 1 and cluster 2 showing high expression only at 1 hpi or 16 hpi, respectively (Fig. 5B).

**Figure 6:**
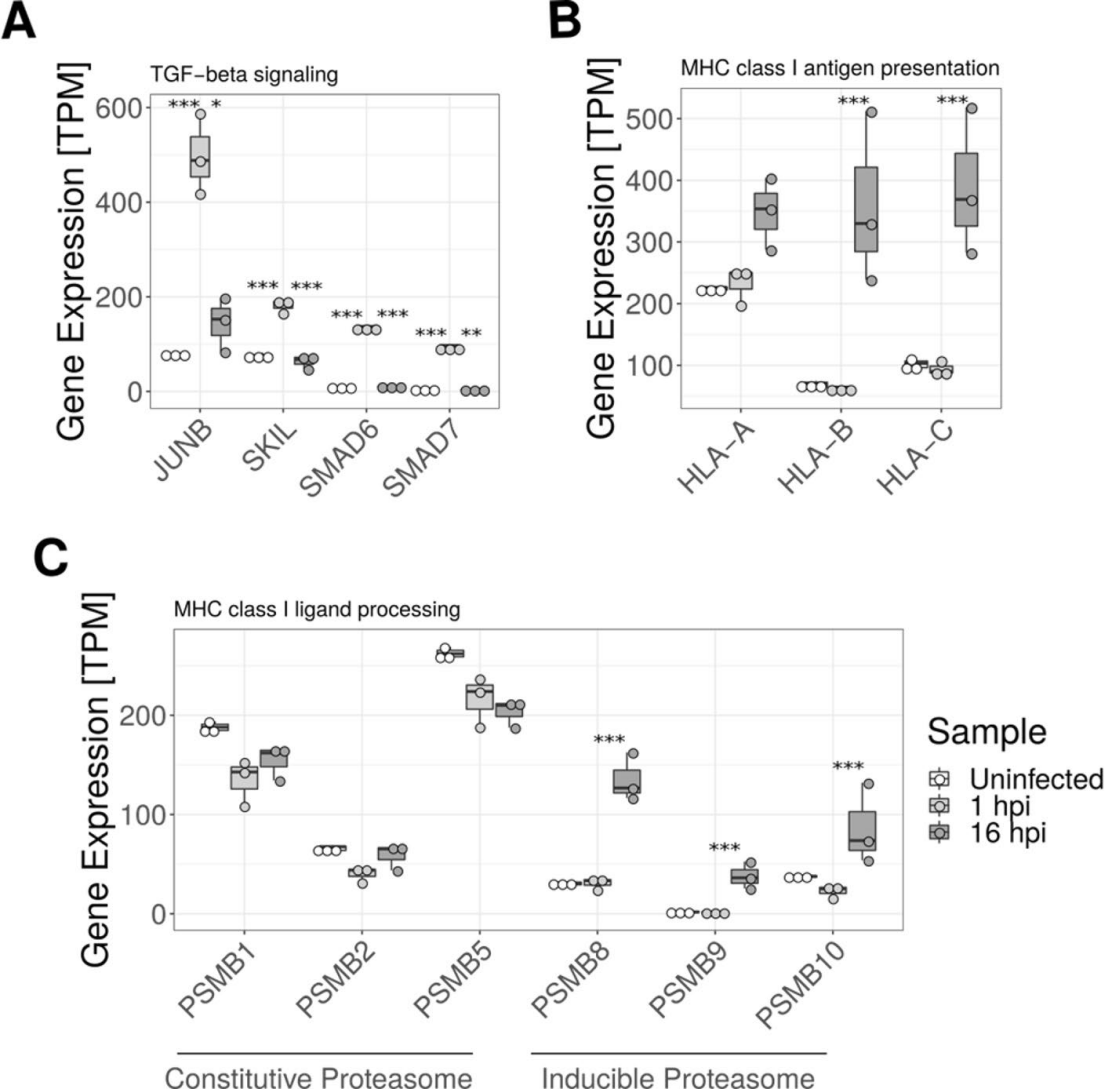
Examples of differentially expressed genes associated with enriched pathways. (**A**) Genes related to TGF-beta signaling (*JUNB*, *SKIL*, *SMAD6*, *SMAD7*). (**B**) Human leukocyte antigen (*HLA*) *A/B/C*. (**C**) Constitutive and inducible proteasomal subunits involved in processing of HLA associated peptides. *p < 0.05, **p < 0.01, ***p < 0.001.

By performing functional enrichment analysis, expression cluster 1 was enriched with 5 reactome pathways, whereas cluster 2 was enriched with 33 reactome pathways (of these, 5 and 10, respectively, of the most significant pathways are displayed in Fig. 5C). While the enriched reactome pathways of cluster 1 can be linked to various cellular signalling pathways, the pathways of cluster 2 are mostly related to intracellular infection such as interferon signalling, MHC Class I presentation, and phagocytosis, which in cluster 2 is upregulated at 16 hpi and thus correlates with the cells harbouring intracellular bacteria. Of interest, the human leukocyte antigen B and C (*HLA-B* and *HLA-C*) were induced by infection at 16 hpi, and *HLA-A* was not found differentially expressed (Fig. 6B). Furthermore, the inducible proteasomal subunits increased expression from 1 to 16hpi, whereas the constitutive subunits remained unchanged (Fig. 6C). The cytokine IL-15 was found to be increased by more than four-fold at 16hpi compared to 1hpi (*p < 0.05, see Supplementary Table S2). In cluster 1, four genes related to TGF-beta signalling—*JUNB*, *SKIL*, *SMAD6*, and *SMAD7* - were transiently upregulated at 1 hpi compared to the two other time-points, indicating the involvement of these genes in the initial host response to bacterial colonization (Fig. 6A).

### Validation of RNA-Seq data using qPCR

Finally, by performing qPCR on independent samples, we quantified 4 genes that in the RNA-Seq data were shown to have significant differential expression between the time points and normalized these to a housekeeping gene followed by comparison to the RNA-Seq data using Pearson correlation. This analysis showed a good correlation (R^2^ = 0.91, *p* = 0.002, Supplementary Fig. S6) between the two methods for the human genes (*JUNB*, *SMAD6*, *PSMB10*, *HLA-B*). It was not possible to retrieve data of high enough quality and with adequate signal from the bacterial gene analysis to enable a meaningful validation of the RNA-Seq results for the expression of the bacterial genes, possibly due to overwhelming amount of human genomic material compared to bacterial in the co-culture samples.

## Discussion

*S. aureus* has inhabited humans for presumably millions of years, and is still found associated with the anterior nares in around 37% of the human population^15^. Consequently, the bacterium has evolved an extensive array of metabolic and defence pathways to survive in close association with humans. There are several examples of specific interaction with human cells and proteins, which have required long evolutionary development^16–18^.

Once *S. aureus* finds itself on the “wrong” side on our bodily surfaces, invasive infections may develop that can be extremely hard to treat^19^. A behaviour believed to contribute significantly to the resilience of *S. aureus* when causing serious infections such as infective endocarditis, is cellular invasion^20^. This invasion is thought to be primarily mediated by the *S. aureus* fibronectin-binding protein A (FnBPA) together with other virulence factors such as clumping factor A (ClfA)^21^. Once inside the human cell, the bacterium may form dormant, resilient colonies^22^ that can persist even during antibiotic treatment. It may also undergo intracellular proliferation and ultimately escape the endothelial cell in large numbers causing local tissue damage. Release of intracellular bacteria into the bloodstream could then lead to metastatic dispersal and infection of other organ sites^23^.

Understanding the invasive and intracellular pathogenesis of *S. aureus* during intravascular infections is therefore key to improving treatment of *S. aureus* bloodstream infections. By identifying specific virulence factors critical for establishment and persistence of infection, it would thus be possible to precisely target these pathogen pathways during treatment. Furthermore, more knowledge on the host response during infection will enable strategies to be devised that can reduce the severity of the symptoms or enhance specific host immune pathways using targeting treatments.

In this study, we conducted controlled in vitro infection and invasion of primary human endothelial cells and employed dual RNA-Seq to examine both the bacterial and host response during colonization and infection.

For both the bacterial and endothelial cell, we saw distinct temporal expression profiles with 521 and 295 differentially expressed genes after logFC shrinkage for *S. aureus* and HUVECs, respectively, when comparing pre-infection, 1 hpi, and 16 hpi. For *S. aureus* in general, we saw an upregulation in the expression of genes related to iron scavenging and a downregulation of genes related to quorum sensing and toxins after infection. Specifically, for the *agr* quorum sensing system, we saw a significant downregulation in the *agrA* and *agrC* at 1 hpi. The quorum sensing agr system is known to be upregulated during high cell densities such as in broth cultures, and would thus be highly expressed in the inoculum, but downregulated after infection possibly due to a reduction in bacterial cell density. Furthermore, in vivo sequestering of the signal molecule (autoinducing peptide, AIP) by the human apolipoprotein B^24^ potentially also contributes to *agr* downregulation during bloodstream infections. Due to cellular invasion at 16 hpi, the upregulation of *agrA* and *agrC* at this timepoint could potentially be explained by a response to intracellular confinement leading to accumulation of AIP^25^. The *agr* quorum sensing system is moreover known to upregulate various toxins such as the γ-hemolysins, which could explain the observed co-expression pattern at 1 hpi^26^, though this did not result in a corresponding shift in γ-hemolysin toxin expression at 16 hpi.

We also observed an upregulation in several factors of the iron-regulated surface determinant (Isd) system at 16 hpi. The iron scavenging genes are primarily important for the bacterium in iron-limited environments, and would thus ideally be downregulated in iron-rich medium such as TSB^27^ and upregulated in iron-limited medium such as serum-based basal medium or during vascular infections. Given that accessible iron is limited in the blood and hence an important limiting factor for *S. aureus* growth and survival in the bloodstream^27,28^, it might be that the bacterium utilizes cellular invasion as a means to gain access to intracellular storages of iron, which accounts for over 90% of the total iron content in humans^28^. Alternatively, IsdB has also been linked to both adherence and invasion of host cells^29^, and thus the transcriptional upregulation of at least *isdB* could alternatively imply an increased dependence on this virulence factor during vascular infection.

In the endothelial cells, an upregulation of a large cluster of genes was seen at 16 hpi compared to the other time-points. Many of these genes are related to responses to invasive infection such as interferon signalling genes and antigen processing genes. Specifically, we saw an upregulation of both *HLA-B*, *HLA-C*, and the inducible proteasome subunits (*PSMB8*, *PSMB9*, *PSMB10*) at 16 hpi, whereas *HLA-A* was not found differentially expressed. Since HLA class I is involved in presentation and thus detection of intracellular pathogens^30^, whereas PSMB8, PSMB9, and PSMB10 are induced upon cellular stress and becomes integral components of the 20S proteasome^31^ that degrades intracellular antigens for presentation^32^, this indicates an activation of intracellular defence systems to combat the invading pathogen employed by the HUVECs or a bacteria-induced response that enables immune evasion. Our results are furthermore comparable to what was reported by Matussek and co-workers, who too saw an upregulation of *HLA-C* and *PSMB*s at 18 hpi using microarray analysis with a clinical *S. aureus* isolate^33^. Furthermore, we observe an increase of IL-15 expression, which promote T-cell proliferation and migration^34^. IL-15 has also been shown to increase angiogenesis^35^ hereby preventing hypoxic damage to tissue^36^, which could be a mechanism elicited to cope with infection-induced ischemia.

In the endothelial cells, we additionally identified a cluster with upregulation at 1 hpi and then downregulation at 16 hpi. This cluster was enriched for TGF-beta signalling, which through a complex regulatory network in a context-dependent manner controls various cell functions related to cell proliferation, survival, and apoptosis^37^. Thus, it may be inferred that the initial response of the endothelial cells to infection is induction of apoptosis followed later at 16 hpi by a response to the intracellular presence of bacteria by upregulation of genes related to antigen presentation to CD8 + T cells. Indeed, others have seen apoptotic changes in endothelial cells at 1 hpi as a result of *S. aureus* cell invasion^38^. However, cluster 2, where the genes were only upregulated after 16 hpi, was also enriched for genes related to apoptosis, and thus apoptosis could conversely be a late response to infection. This is somewhat in line with what Moreilhon *et al.* saw using microarray analysis on infected human airway epithelial cells with another *S. aureus* strain, where an initial anti-apoptotic response was seen at 3 hpi followed by an apoptotic phase at 9 hpi^39^. The same study together with another microarray study on Hep-2 cells furthermore found *JUNB* upregulated at early time points similar to our data^39,40^. In regard to the role of *JUNB*, others have found this gene to be involved in inhibiting colonization of ketanocytes of the skin^41^, and thus could be a host response towards bacterial colonization.

In the present study a focus was placed on infection by one specific reference *S. aureus* strain. It would be interesting to utilize the methodology to investigate the genetic expression in *S. aureus* strains with different virulence profiles during endothelial infection and compare with the host response to these strains. Bielecki *et al.* for example, used single RNA-Seq to investigate both similarities and marked differences in bacterial RNA-Seq profiles during growth in patient urine samples containing different uropathogenic *E. coli* strains from various phylogenetic groups^42^. In this regard, others have observed an upregulation of *PSMB8* in HUVEC cultures at ≈ 8 hpi or 18 hpi using microarray analysis with different *S. aureus* isolates^22,33^ indicating that at least upregulation of some genes could be similar between strains. Thänert *et al.* on the other hand used dual RNA-Seq to investigate the difference in transcriptional response, when infecting two different mouse strains with the same *S. aureus* SH1000 strain, and found differences in both the host and bacterial response^10^. In the current study, we used HUVEC cells that have been pooled from multiple donors to even out these potential differences of each donor, but in future studies it would be interesting to investigate possible individual differences in separate donor samples. Such studies may help explain why certain individuals are more susceptible to severe *S. aureus* BSI and more difficult to treat than others. To study the transcriptional response of individual HUVECs to invasion by single or multiple bacteria, future interesting experiments could use RNA-Seq analysis of sorted infected cells (e.g. by using fluorescence-activated cell sorting (FACS))^43^. Furthermore, it could also be relevant to include the analysis of non-infected cells to look at potential “bystander” effects. Lastly, though HUVECs are often used in modelling of vascular infections with *S. aureus*, the use of other endothelial cells is also possible such as human dermal microvascular endothelial cells (HDMEC) or human pulmonary artery endothelial cells (HPAEC) or immortalized cell lines derived hereof. Infection in different types of cells would likely portray both similarities and differences in the transcriptional response as seen by others^44^.

In conclusion, we present to our knowledge the first global simultaneous genetic expression analysis of host and pathogen during *S. aureus* endothelial intracellular infection. The findings include for *S. aureus* the identification of an upregulation of iron scavenging genes and a downregulation of toxin genes during these infections, which could thus be potential targets for future treatments. Overall, the data material should provide an important basis for interpretation of both previous studies in a broader context, assist the design and data interpretation in future studies on specific pathogenic pathways, and overall assist in creating a more complete picture of the stepwise process of invasion and *S. aureus* intracellular infection and survival. This knowledge is critical to help overcome the current challenge of treating *S. aureus* bloodstream infections.

## Methods

### Bacteria, cells, and culture conditions

The *Staphylococcus aureus* ATCC29213 reference strain and primary human umbilical vein endothelial cells (HUVEC from pooled donors, passage 2–3, PromoCell GmbH, Heidelberg, Germany) were used for all experiments. Confocal laser scanning microscopy was performed with a GFP-producing ATCC29213 strain as previously described^11^. Prior to experiments, the bacteria were grown until exponential growth phase in tryptic soy broth (2½h at 37 °C), and bacterial stock solution could then be prepared by centrifugation and resuspension of pellet in phosphate buffered saline (PBS) followed by adjustment to OD_600_ = 0.1. HUVECs were cultured in basal medium (Endothelial Cell Growth Medium 2, PromoCell) with fetal bovine serum (FBS, PromoCell) and penicillin–streptomycin (PS, Gibco), liberated by trypsination and seeded into microtiter plates. Prior to experiments, the confluent HUVEC 24-well microtiter plate cultures were washed 3 times in PBS and filled with 0.5 ml basal medium + FBS without PS.

### Endothelial invasion assay

Microtiter plates were inoculated with 20 μl of bacterial stock suspension corresponding to a multiplicity of infection (MOI) of 16, followed by centrifugation at 300* g* for 5 min to sediment the bacteria. After 1½ hours post-infection (hpi), wells were supplemented with lysostaphin (10 μg/ml) + gentamicin (100 μg/ml) and were thus present for the rest of the experiment. HUVEC samples were collected by washing 3 times in PBS followed by trypsinization for 10 min, centrifugation, and washing the pellet followed by centrifugation and resuspension of the pellet in 100 μl PBS. Samples were then snap-frozen in liquid nitrogen and kept at -80 °C until RNA isolation.

### RNA isolation, rRNA depletion, and RNA sequencing

Pellets were resuspended in 700 μl acetate buffer (20 mM NaOAc, 1 mM EDTA, 0.5% SDS, pH 4.5) and transferred to FastPrep tubes containing lysing matrix B. Cells were lysed in a Thermo Savant FastPrep FP120 Cell Homogenizer at 6.0 m/sec for 3 × 40 s with 1 min pause on ice in between runs, and then centrifuged for 5 min at 20,000 *g*. Lysate was transferred to tubes containing 500 μl phenol solution (2:1 phenol in water adjusted to pH 4.5 with NaOAc) and 100 μl chloroform followed by incubation at 65 °C at 1100 rpm in a thermomixer for 15 min with vortexing every 2 min. After centrifugation, the aqueous phase was transferred into tubes containing 500 μl chloroform followed by vortexing, centrifugation, and transferring to new tubes. The RNA was precipitated using 96% EtOH and 10vol% 3 M NaOAc on ice for ≥ 1 h. Tubes were centrifuged for 60 min at 4 °C, and pellets were washed in ice cold 70% EtOH followed by centrifugation for 10 min. Supernatant was removed and pellets were then air-dried and dissolved in sterile ddH2O. RNA quantity was measured using Nanodrop spectrophotometer, and RNA quality was then verified by fragment analysis using a Fragment Analyzer (Agilent), followed by Ribo-Zero Gold Kit (MRZE724, Epidemiology/Illumina) treatment on the 3 samples from each group to remove both human and bacterial rRNA. After DNase treatment, library preparation was performed using the NEBNext Ultra Library Prep Kit (NEB) followed by quality assessment by the Fragment Analyzer and sequencing on the NovaSeq 6000 platform (Illumina).

### Accession numbers

The RNA-Seq data has been archived at NCBI Gene Expression Omnibus that can be found at: https://www.ncbi.nlm.nih.gov/geo/ under accession number GSE151135.

### Confocal laser scanning microscopy

Confocal laser scanning microscopy was performed directly into 6-well microtiter plates using an Olympus FV1000MPE microscope and Olympus FV10‐ASW software. Prior to microscopy, cells were stained with either LIVE/DEAD cell viability kit (L7012, Thermofisher) or Acti-stain 670 phalloidin (Cytoskeleton Inc.) according to manufacturer’s protocol.

### Data processing, statistics, and bioinformatic analysis

The raw reads were preprocessed using cutadapt^45^ on default settings and base qualities assessed using FastQC^46^. Samples with HUVEC cells were mapped to the human reference genome GRCh38.84 using STAR^47^. Samples with *S. aureus* cells were mapped to the NC_007795.1 reference genome using bwa mem^48^. Read duplicates were removed using Picard^49^ and gene coverages computed using featureCounts^50^. Differential expression analysis was conducted using the DESeq2 workflow with Benjamini–Hochberg corrected Wald tests coupled to logFC shrinkage^14^. Differential expression was considered significant for genes with adjusted *p*-values below 0.05 and adjusted log fold change magnitudes above 1.0. Gene expression trajectories of differentially expressed genes were generated using complete-linkage hierarchical clustering with Euclidean distance on the log2 fold change transformed expression values relative to the average of the given gene. Genes were not enforced to have a significant differential expression in both comparisons to be included in the analysis.

Enrichment for *S. aureus* virulence factor classes was done with the Fisher’s exact test with contingency matrices of the design (gene cluster vs virulence factor class). Human pathway enrichment analysis was conducted on the gene clusters using the clusterProfiler package^51^ and the Reactome Pathway annotation database^52^. A pathway was considered significantly enriched when its adjusted *p*-value was < 0.05. All data processing and figure generation was done using the R software version 3.6.2^53^.

## Supplementary Information

Supplementary Information.
