# Supplementary material for: Bacteria-host transcriptional response during endothelial invasion by Staphylococcus aureus
Source: Sci Rep. 2021 Mar 16;11:6037. doi: 10.1038/s41598-021-84050-x (PMC7966777; doi:10.1038/s41598-021-84050-x)
Supplement: Supplementary file 1 — Supplementary Information. [file 41598_2021_84050_MOESM1_ESM.pdf]

Supplementary Information

Title: Bacteria-host transcriptional response during endothelial invasion by *Staphylococcus aureus*

Authors: Rasmus Birkholm Grønnemose[1], Christian Garde[2], Claes Søndergaard Wassmann[3], Janne Kudsk Klitgaard[1,3], Ronni Nielsen[4], Susanne Mandrup[4], Andreas Holm Mattsson[2], Thomas Emil Andersen[1*]

Affiliations:

[1]Research unit of Clinical Microbiology, University of Southern Denmark and Odense University Hospital, Odense, Denmark

[2]Evaxion Biotech A/S, Copenhagen, Denmark

[3]Research unit of Molecular Microbiology, Department of Biochemistry and Molecular Biology, University of Southern Denmark, Odense, Denmark

[4]Functional Genomics and Metabolism Research Unit, Department of Biochemistry and Molecular Biology, University of Southern Denmark, Odense, Denmark

Correspondence:
Thomas Emil Andersen, Research unit of Clinical Microbiology, University of Southern Denmark and Odense University Hospital, J.B. Winsløws Vej 21.2, 5000 Odense, Denmark. E-mail: thandersen@health.sdu.dk

Supplementary Information

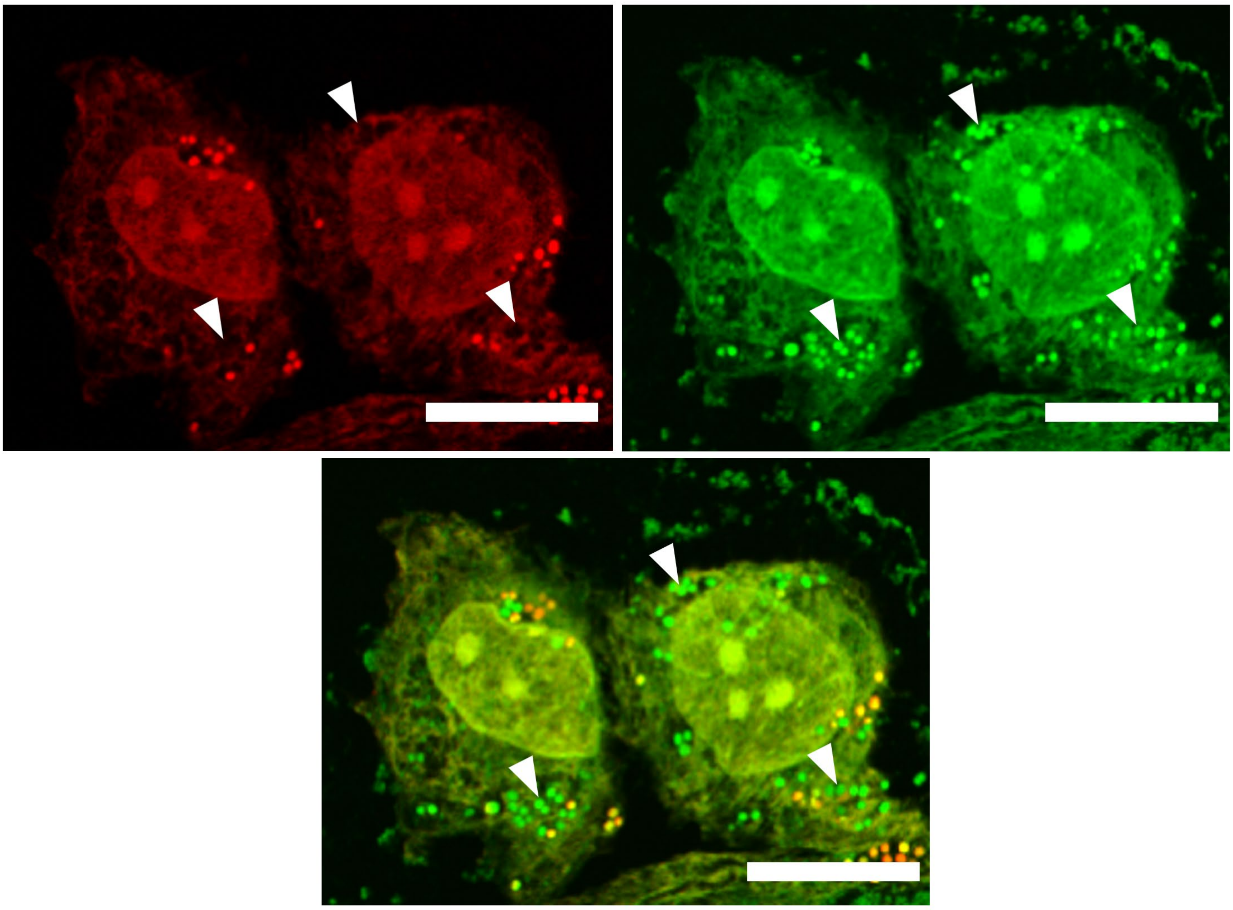

Supplementary Figure S1: Infected HUVECs were visualized by confocal laser scanning microscopy. At 1 hpi, bacteria are localised inside vacuolar compartments (indicated by arrows). Cells were stained with LIVE/DEAD cell viability stain (propidium iodide, red, and SYTO9, green). Scale bars indicate 20 µm. Samples from 16 hpi can be found in Figure 2C.

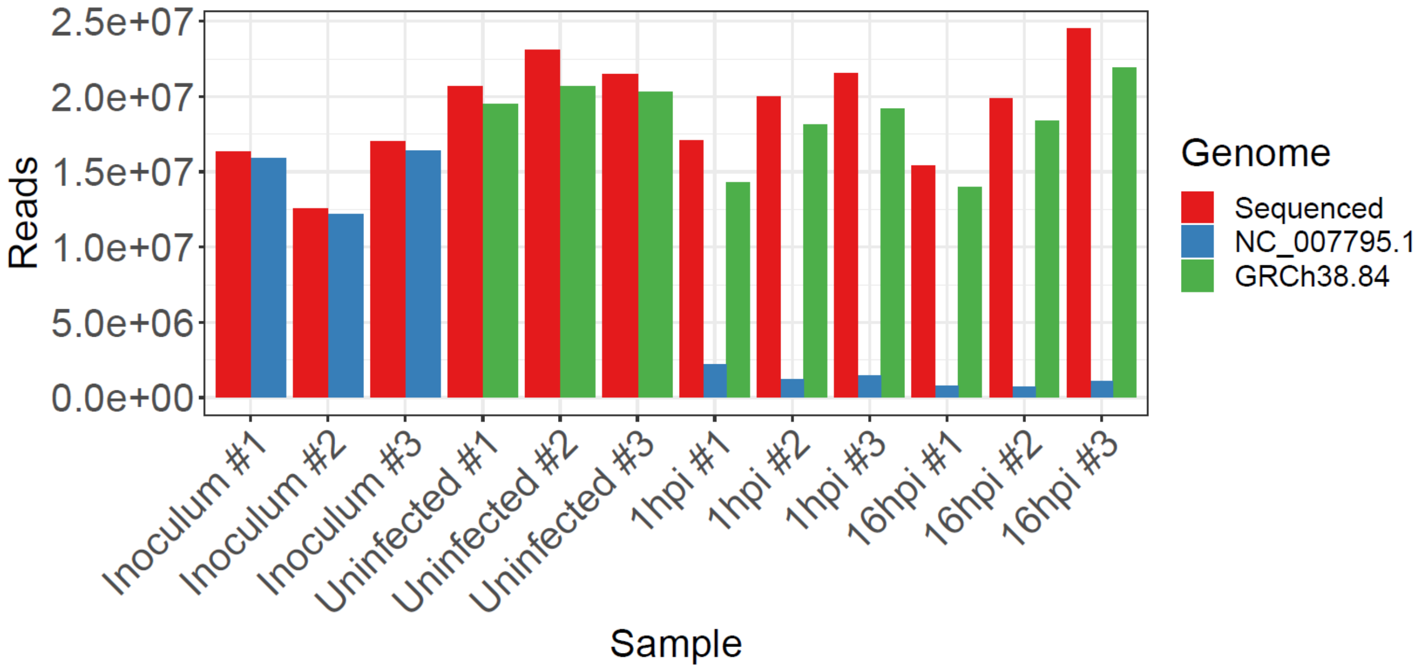

Supplementary Figure S2: Total coverage depth mapped to the triplicates of the bacterial inoculum, the uninfected HUVECs, 1 hpi, and 16 hpi. The reads were mapped to either the bacterial genome (NC_007795.1, in blue) or the human genome (GRCh38.84, in green).

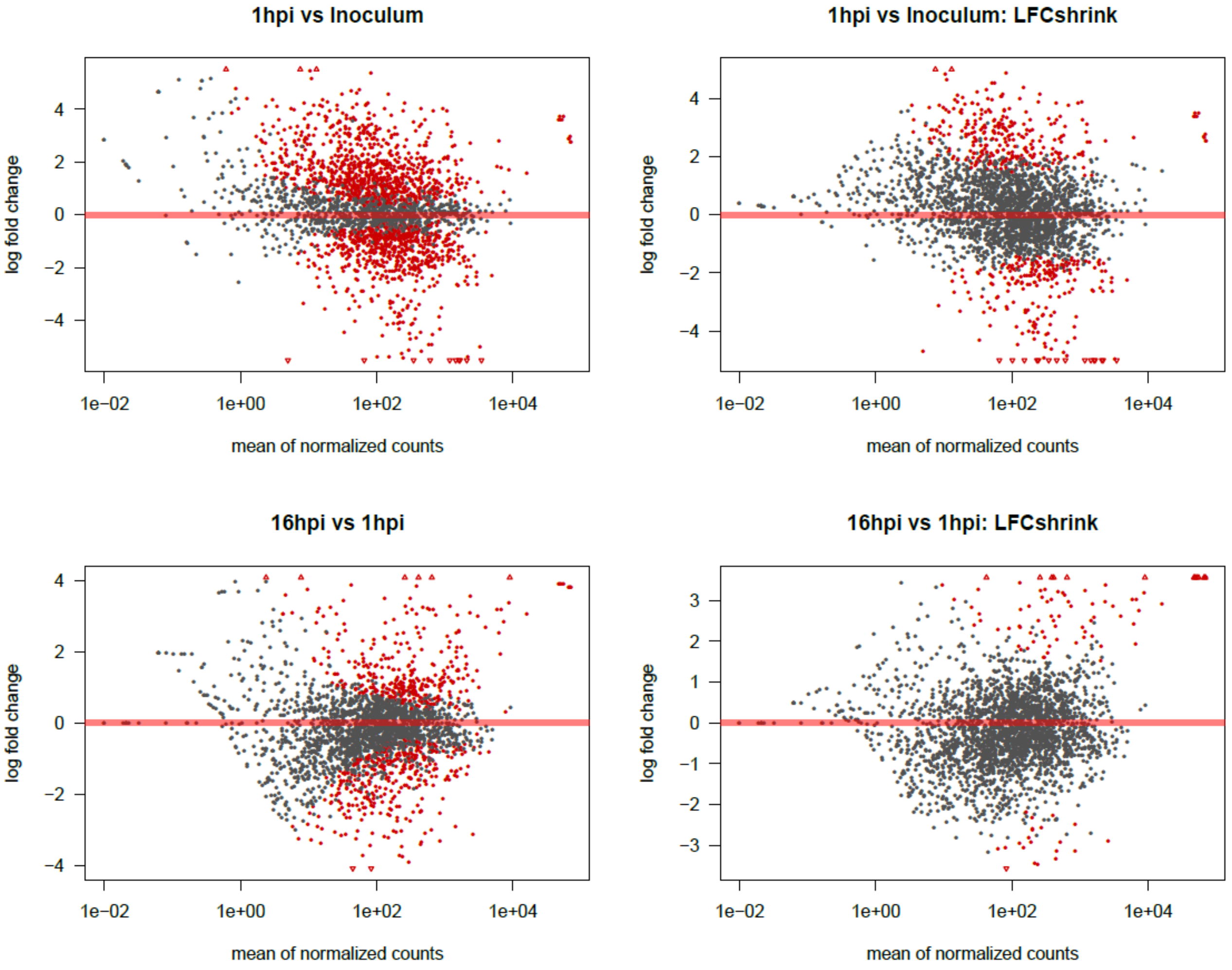

Supplementary Figure S3: Using logFC shrinkage to penalize low coverage for *S. aureus* between the two comparisons (1 hpi vs. inoculum (top panels) and 16 hpi vs 1 hpi (bottom panels)). Differentially expressed genes for *S. aureus* above the threshold before (left panels) and after logFC shrinkage (right panels) are marked in red.

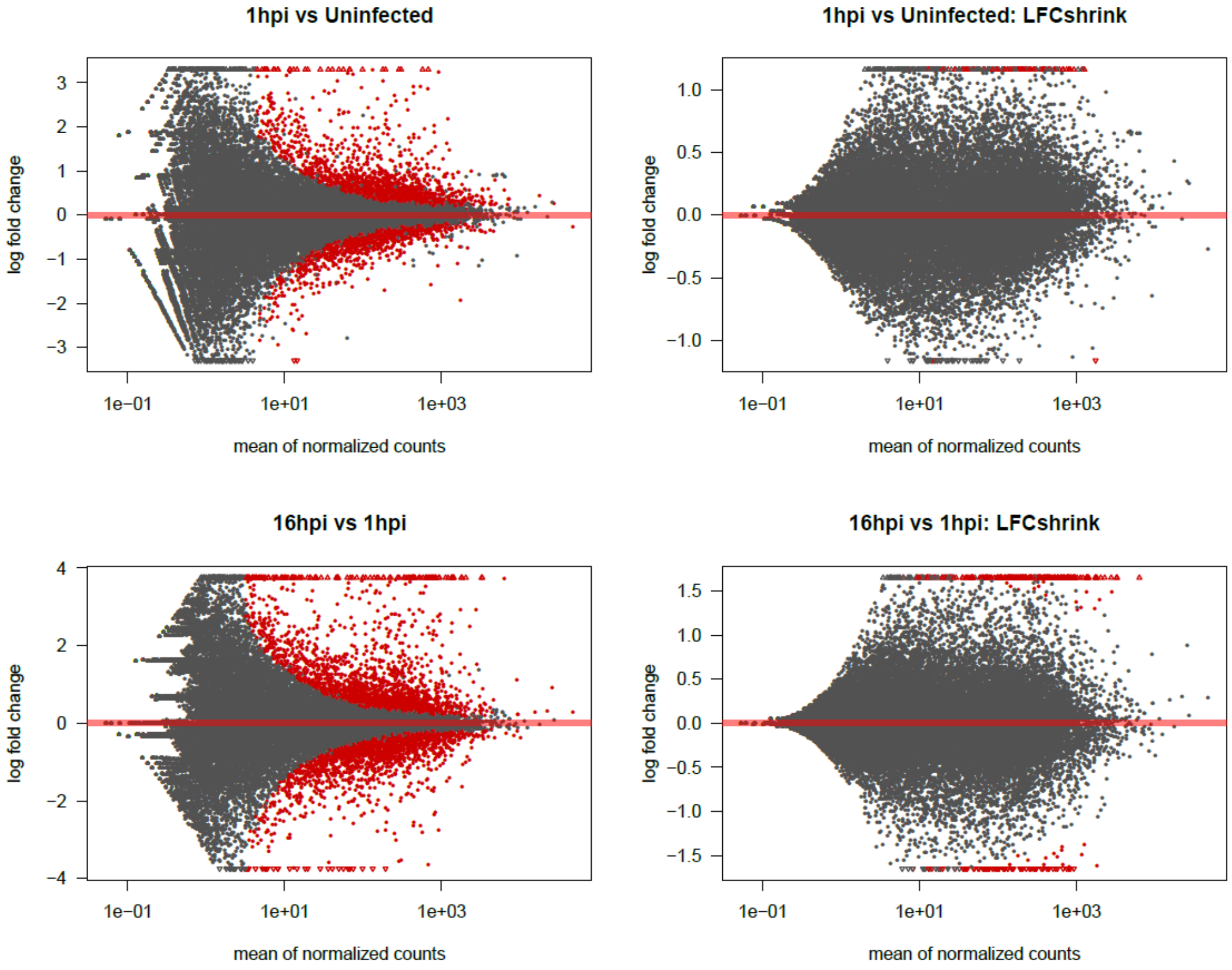

Supplementary Figure S4: Using logFC shrinkage to penalize low coverage for HUVECs between the two comparisons (1 hpi vs. uninfected (top panels) and 16 hpi vs 1 hpi (bottom panels)). Differentially expressed genes for HUVECs above the threshold before (left) and after logFC shrinkage (right) are marked in red.

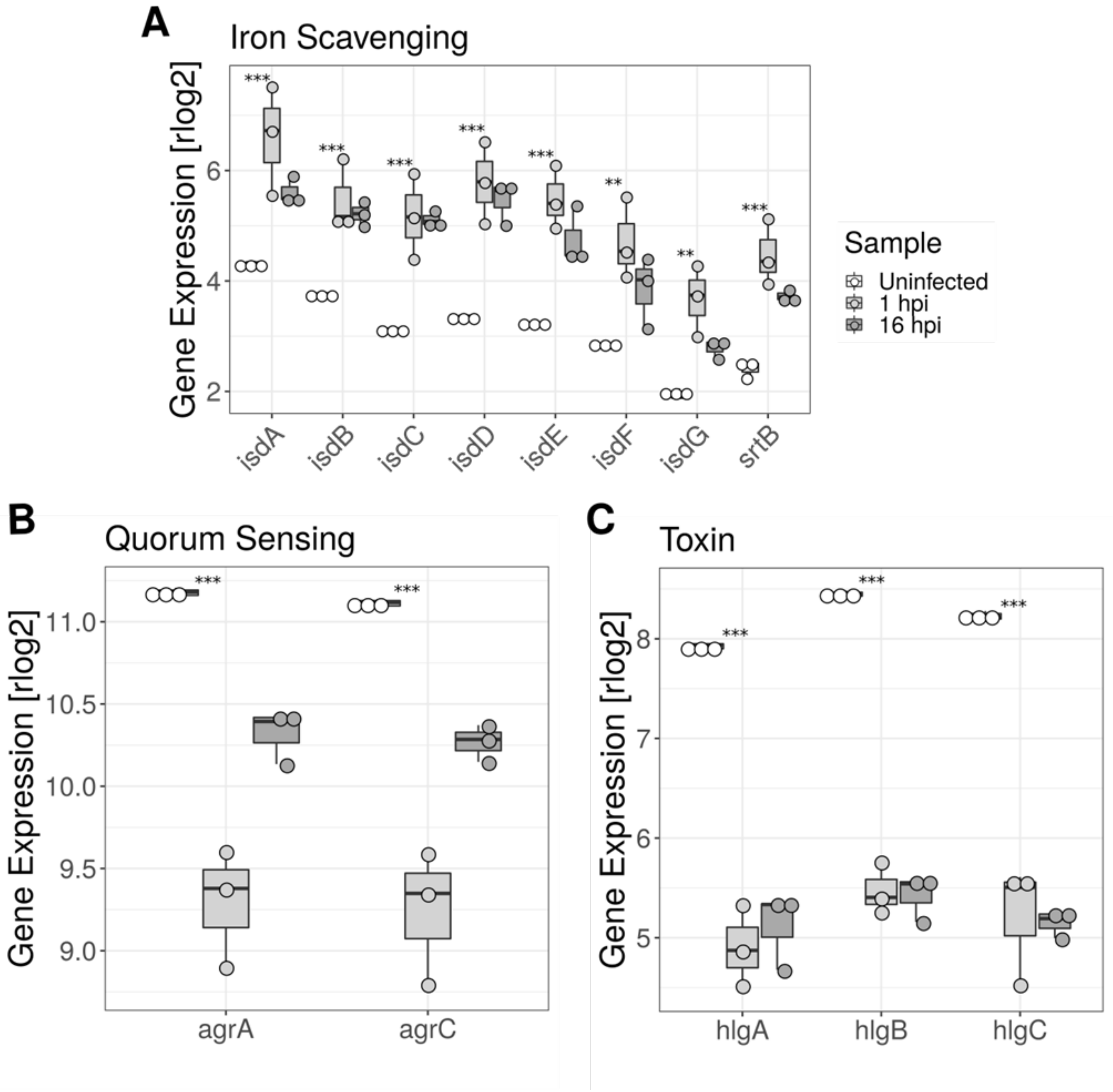

Supplementary Figure S5: Examples of differentially expressed genes related to iron scavenging (Isd), quorum sensing (agr), and toxins (γ-hemolysin, hlg) at different time-points. *p<0.05, **p<0.01,
***p<0.001.

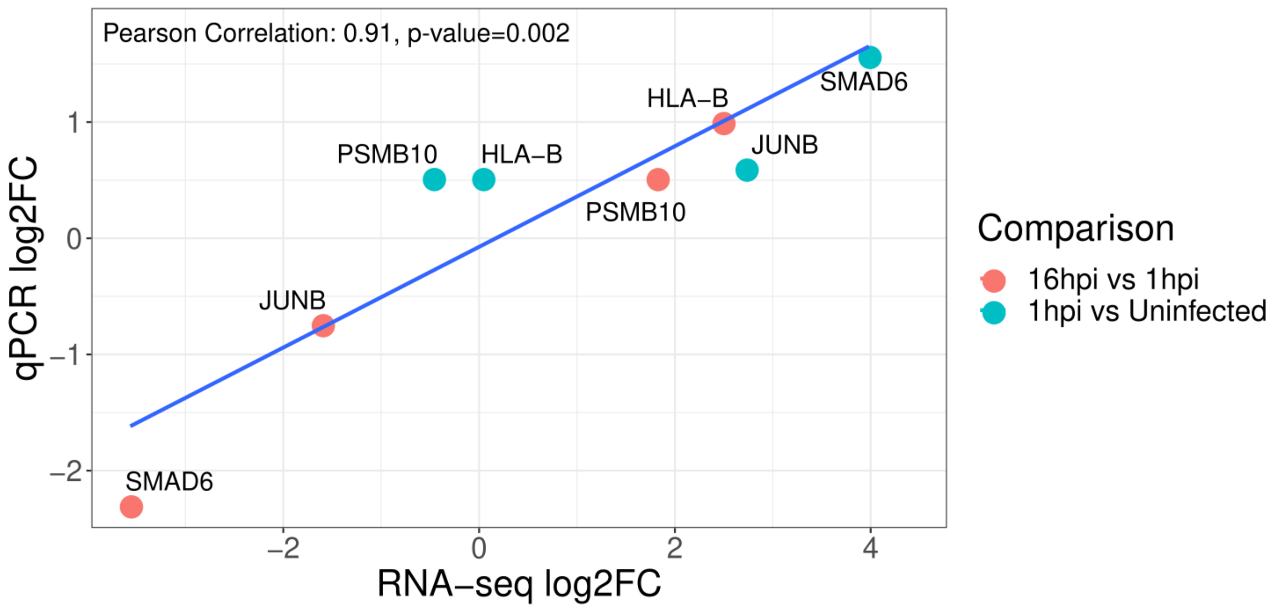

Supplementary Figure S6: Pearson correlation between RNAseq and qPCR in the two comparisons (red and blue for 16 hpi vs. 1 hpi and 1 hpi vs. uninfected, respectively) for 4 selected human genes (HLA-B, JUNB, SMAD6, PSMB10). Due to low signal, it was not possible to perform a meaningful comparison for the bacterial genes.

For qPCR, RNA was purified by first lysing the cells with FastPrep FPI20 followed by hot-phenol treatment. The samples were then treated with NEB DNase I kit (New England Biolabs) to remove DNA. For each 180 ng of RNA, 0.2 units of DNase I was added and incubated for 10 min at 37 °C. The DNase I was inactivated at 75 °C for 10 min. cDNA was synthesized using the High Capacity cDNA Reverse Transcription kit (K1671, Thermo Scientific). The samples were then processed in the PCR machine using 25 °C for 10 min, 37 °C for 60 min, 85 °C for 5 min followed by a hold at 4°C. qPCR was performed in a 384-well plate in duplicates. RealQ Plus 2x Master Mix Green (A323406, Ampliqon) was mixed with approx. 10 ng cDNA, 750 nM forward primer, and 750 nM reverse primer (see Supplemental table 4 for primers). The samples were amplified in a LightCycler480 using: pre-incubation at 95°C for 15 min, 45 cycles of 95°C for 15 s, 60°C for 45 s, and 72°C for 45 s. The cycling was followed by a hold at 95°C for 5 s, hold at 60°C for 20 s and a continuous melt from 60°C to 97°C ramping 0.11°C/s with 5 acquisitions per degree. The expression of the human genes was normalized to the human householding gene B2M, while the expression of the bacterial genes was normalized to the housekeeping gene gyrB.

Gene name	Function 1	Function 2	Function 3	Reference
clfA	Adhesion			1
clfB	Adhesion			1
coa	Immune evasion	Biofilm formation	Adhesion	1
vwb	Immune evasion	Biofilm formation	Adhesion	1
icaA	Biofilm formation			1
icaB	Biofilm formation			1
icaC	Biofilm formation			1
icaD	Biofilm formation			1
icaR	Regulation			1
cna	Adhesion			1
eap	Adhesion	Immune evasion		1
ebpS	Adhesion			1
fnbA	Adhesion	Invasion		1
fnbB	Adhesion	Invasion		1
sdrC	Biofilm formation	Adhesion		1
sdrD	Adhesion			1
sdrE	Immune evasion			1
capA	Immune evasion			1
capB	Immune evasion			1
capC	Immune evasion			1
capD	Immune evasion			1
capE	Immune evasion			1
capF	Immune evasion			1
capG	Immune evasion			1
capH	Immune evasion			1
capI	Immune evasion			1
capJ	Immune evasion			1
capK	Immune evasion			1
capL	Immune evasion			1
capM	Immune evasion			1
capN	Immune evasion			1
capO	Immune evasion			1
capP	Immune evasion			1
efb	Adhesion	Immune evasion		1
aur	Immune evasion	Dispersal		3
hysA	Dispersal			1
sak	Dispersal			1
geh	Immune evasion			1
sspB	Immune evasion			1
sspC	Regulation			1
adsA	Immune evasion			4
chp	Immune evasion			4
sbi	Adhesion			4
scn	Adhesion			1
spa	Adhesion			1
isdA	Adhesion	iron scavenging		5,7

isdB	iron scavenging	5
isdC	iron scavenging	5
isdD	iron scavenging	5
isdE	iron scavenging	5
isdF	iron scavenging	5
isdG	iron scavenging	5
isdH	iron scavenging	5
isdI	iron scavenging	5
srtA	Surface protein sorting	8
srtB	Surface protein sorting	8
agrA	Quorum sensing	2
agrB	Quorum sensing	2
agrC	Quorum sensing	2
adrD	Quorum sensing	2
hla	Toxin	1
hlb	Toxin	1
hld	Toxin	1
eta	Toxin	2
etb	Toxin	2
lukS-PV	Toxin	1
lukF-PV	Toxin	1
sea	Toxin	1
seb	Toxin	1
sec1	Toxin	6
sec3	Toxin	6
sed	Toxin	1
see	Toxin	1
seg	Toxin	1
seh	Toxin	1
sei	Toxin	1
sel	Toxin	1
sem	Toxin	1
sen	Toxin	1
seo	Toxin	1
sep	Toxin	1
selk	Toxin	2
selq	Toxin	2
tsst-1	Toxin	1
fib	Adhesion	1
lukE	Toxin	1
lukD	Toxin	1
lukM	Toxin	1
hlgC	Toxin	1
hlgB	Toxin	1
hlgA	Toxin	1
sexo	Toxin	1
set1	Toxin	1
set2	Toxin	1
set3	Toxin	1
set4	Toxin	1

set5	Toxin		1
set6	Toxin		1
set7	Toxin		1
set8	Toxin		1
set9	Toxin		1
set10	Toxin		1
set11	Toxin		1
set12	Toxin		1
set13	Toxin		1
set14	Toxin		1
set15	Toxin		1
set16	Toxin		1
set17	Toxin		1
set18	Toxin		1
set19	Toxin		1
set20	Toxin		1
set21	Toxin		1
set22	Toxin		1
set23	Toxin		1
set24	Toxin		1
set25	Toxin		1
set26	Toxin		1
set30	Toxin		1
yent1	Toxin		1
yent2	Toxin		1
sent-like	Toxin		1
SEnt	Toxin		1
esxA	Secretion		1
esaA	Secretion		1
essA	Secretion		1
esaB	Secretion		1
essB	Secretion		1
essC	Secretion		1
esaC	Secretion		1
esxB	Secretion		1
isb	Immune evasion		1
nuc	Exoenzyme		1
lip	Immune evasion	Exoenzyme	1
atl	Adhesion		1
ebh	Adhesion		1
psmα1	Toxin		4
psmα2	Toxin		4
psmα3	Toxin		4
psmα4	Toxin		4
psmβ1	Toxin		4
psmβ2	Toxin		4
sarA	Regulation		2
sarT	Regulation		2
sarU	Regulation		2
sarH1	Regulation		2

rot	Regulation	2
mgrA	Regulation	2
sigB	Regulation	2
sigH	Regulation	2
saeR	Regulation	2
saeS	Regulation	2
saeP	Regulation	2
saeQ	Regulation	2
srrA	Regulation	2
srrB	Regulation	2
airSR	Regulation	2

Supplementary Table S1: List of virulence factors used for the gene enrichment analysis in Figure 4C . The list is based on [1-8].

Supplementary Table S2

Six tables containing the differential expressed genes for *S. aureus* and HUVECs between all 6 comparisons (1 hpi vs. inoculum/uninfected, 16hpi vs. inoculum/uninfected 16hpi vs. 1hpi).

S. aureus - 1hpi vs. inoculum

Locus_id	Protein_id	Gene_id	Name	Description	baseMean	log2FoldChange	lfcSE	stat	pvalue	padj	Cluster
SAOUHSC_00187	YP_498784.1	3919501	pflB	Formate acetyltransferase (EC 2.3.1.54) (Pyruvate formate-lyase)	3453.909	-6.83191	0.153241	-38.0571	0	0	Cluster 1
SAOUHSC_00608	YP_499171.1	3918927	adh	Alcohol dehydrogenase (ADH) (EC 1.1.1.1)	1667.49	-7.0422	0.248233	-24.3408	7.3E-131	9.2E-128	Cluster 1
SAOUHSC_01450	YP_499971.1	3920227		Uncharacterized protein	1612.479	-7.77182	0.279946	-24.1897	2.9E-129	2.4E-126	Cluster 1
SAOUHSC_00188	YP_498785.1	3919502	pflA	Pyruvate formate-lyase-activating enzyme (PFL-activating enzyme) (EC 1.97.1.4)	1444.763	-6.32225	0.269448	-19.7524	7.65E-87	4.84E-84	Cluster 1
SAOUHSC_02941	YP_501393.1	3921647		Anaerobic ribonucleoside-triphosphate reductase-activating protein (EC 1.97.1.-)	455.598	-5.15122	0.210942	-19.6795	3.23E-86	1.64E-83	Cluster 1
SAOUHSC_01451	YP_499972.1	3920228	tdcB	L-threonine dehydratase catabolic TdcB (EC 4.3.1.19) (Threonine deaminase)	609.8333	-8.46058	0.397835	-18.7529	1.83E-78	7.74E-76	Cluster 1
SAOUHSC_01452	YP_499973.1	3919897	ald1	Alanine dehydrogenase 1 (EC 1.4.1.1)	347.955	-7.34059	0.37085	-17.0975	1.55E-65	5.61E-63	Cluster 1
SAOUHSC_01788	YP_500293.1	3920430	thrS	Threonine--tRNA ligase (EC 6.1.1.3) (Threonyl-tRNA synthetase) (ThrRS)	3281.621	-4.95399	0.237302	-16.6623	2.46E-62	7.81E-60	Cluster 1
SAOUHSC_02648	YP_501110.1	3921210		L-lactate permease	2186.078	-5.32147	0.260119	-16.6134	5.57E-62	1.57E-59	Cluster 1
SAOUHSC_02582	YP_501044.1	3921578		Putative formate dehydrogenase SAOUHSC_02582 (EC 1.17.1.9)	3000.266	-2.62366	0.103215	-15.7309	9.29E-56	2.35E-53	Cluster 1
SAOUHSC_02680	YP_501142.1	3921242		Nitrate reductase, beta subunit (EC 1.7.99.4)	234.5113	-5.21124	0.271697	-15.4998	3.48E-54	8.02E-52	Cluster 1
SAOUHSC_00569	YP_499138.1	3920611		Uncharacterized protein	1268.479	3.117786	0.13835	15.30749	6.81E-53	1.44E-50	Cluster 5
SAOUHSC_02742	YP_501203.1	3921616		Amino acid transporter, putative	244.4066	-4.03939	0.202368	-15.0191	5.5E-51	1.07E-48	Cluster 1
SAOUHSC_01170	YP_499709.1	3920921	carB	Carbamoyl-phosphate synthase large chain (EC 6.3.5.5) (Carbamoyl-phosphate synthetase ammonia chain)	835.1016	-3.84748	0.191772	-14.8483	7.14E-50	1.29E-47	Cluster 1
SAOUHSC_02781	YP_501241.1	3921436		Uncharacterized protein	244.7367	-5.36699	0.300242	-14.5449	6.29E-48	1.06E-45	Cluster 1
SAOUHSC_01888	YP_500390.1	3920836		Riboflavin synthase, alpha subunit (EC 2.5.1.9)	132.0296	4.005524	0.207677	14.47212	1.82E-47	2.88E-45	Cluster 5
SAOUHSC_02942	YP_501394.1	3921648		Anaerobic ribonucleoside-triphosphate reductase, putative (EC 1.17.4.2)	643.4968	-4.86424	0.267678	-14.4361	3.07E-47	4.57E-45	Cluster 1
SAOUHSC_01448	YP_499970.1	3920226	norB	Quinolone resistance protein NorB	1179.552	-6.83414	0.419881	-13.8947	6.82E-44	9.6E-42	Cluster 1
SAOUHSC_02400	YP_500876.1	3919615		PTS system, mannitol-specific component, putative	382.8435	-4.60841	0.26078	-13.837	1.52E-43	2.03E-41	Cluster 1
SAOUHSC_00206	YP_498803.1	3920362	ldh1	L-lactate dehydrogenase 1 (L-LDH 1) (EC 1.1.1.27)	2101.399	-6.8449	0.424822	-13.7585	4.53E-43	5.74E-41	Cluster 1
SAOUHSC_02684	YP_501146.1	3919703		Assimilatory nitrite reductase [NAD(P)H], large subunit, putative (EC 1.7.1.4)	152.5018	-5.30536	0.317354	-13.5664	6.34E-42	7.64E-40	Cluster 1
SAOUHSC_02581	YP_501043.1	3921577		Uncharacterized protein	707.0486	-2.66148	0.126628	-13.1209	2.5E-39	2.88E-37	Cluster 1
SAOUHSC_02676	YP_501138.1	3921238	nreB	Oxygen sensor histidine kinase NreB (EC 2.7.13.3) (Nitrogen regulation protein B)	422.1198	-3.37404	0.182386	-13.0166	9.85E-39	1.09E-36	Cluster 1
SAOUHSC_01889	YP_500391.1	3920837		Riboflavin biosynthesis protein RibD [Includes: Diaminohydroxyphosphoribosylaminopyrimidine deaminase (DRAP deaminase) (EC 3.5.4.26) (Riboflavin-specific deaminase); 5-amino-6-(5-phosphoribosylamino)uracil reductase (EC 1.1.1.193) (HTP reductase)]	259.2004	4.1706	0.252813	12.54129	4.44E-36	4.68E-34	Cluster 5
SAOUHSC_02678	YP_501140.1	3921240		Respiratory nitrate reductase, gamma subunit (EC 1.7.99.4)	183.9806	-3.65135	0.212294	-12.4891	8.57E-36	8.68E-34	Cluster 1
SAOUHSC_02679	YP_501141.1	3921241		Respiratory nitrate reductase, delta subunit, putative	101.7287	-5.31944	0.356693	-12.1097	9.39E-34	9.15E-32	Cluster 1
SAOUHSC_T00038		3921164			50.99545	4.55411	0.295416	12.03087	2.45E-33	2.3E-31	Cluster 5
SAOUHSC_01168	YP_499707.1	3920919	pyrC	Dihydroorotase (DHOase) (EC 3.5.2.3)	339.0617	-4.08877	0.26513	-11.65	2.29E-31	2.08E-29	Cluster 1
SAOUHSC_02743	YP_501204.1	3921617		Amino acid ABC transporter, permease protein, putative	117.5647	-3.85758	0.247403	-11.5503	7.36E-31	6.43E-29	Cluster 1
SAOUHSC_00330	YP_498919.1	3919595		Uncharacterized protein	69.58169	4.090535	0.269336	11.47464	1.77E-30	1.49E-28	Cluster 5
SAOUHSC_01165	YP_499705.1	3920917		Uracil permease, putative	213.025	-3.695	0.238497	-11.3	1.31E-29	1.07E-27	Cluster 1
SAOUHSC_01887	YP_500389.1	3920835	ribBA	Riboflavin biosynthesis protein RibBA [Includes: 3,4-dihydroxy-2-butanone 4-phosphate synthase (DHBP synthase) (EC 4.1.99.12); GTP cyclohydrolase-2 (EC 3.5.4.25) (GTP cyclohydrolase II)]	245.3414	3.883323	0.25693	11.22219	3.17E-29	2.51E-27	Cluster 5
SAOUHSC_02671	YP_501133.1	3921233		conserved hypothetical protein	157.9785	-3.2679	0.203513	-11.1438	7.68E-29	5.9E-27	Cluster 1
SAOUHSC_T00021		3921340			45.62899	3.625318	0.236163	11.11657	1.04E-28	7.77E-27	Cluster 6
SAOUHSC_02741	YP_501202.1	3921615		Amino acid ABC transporter, permease protein, putative	205.4304	-3.82047	0.2564	-11.0003	3.81E-28	2.76E-26	Cluster 1
SAOUHSC_01171	YP_499710.1	3920922	pyrF	Orotidine 5'-phosphate decarboxylase (EC 4.1.1.23) (OMP decarboxylase) (OMPDCase) (OMPdecase)	224.673	-3.76739	0.255201	-10.844	2.13E-27	1.5E-25	Cluster 1
SAOUHSC_01169	YP_499708.1	3920920	carA	Carbamoyl-phosphate synthase small chain (EC 6.3.5.5) (Carbamoyl-phosphate synthetase glutamine chain)	304.26	-4.42032	0.315796	-10.8308	2.46E-27	1.68E-25	Cluster 1
SAOUHSC_02710	YP_501172.1	3919729	hlgB	Gamma-hemolysin component B (H-gamma-1) (H-gamma-I) (Leukocidin f subunit)	205.5472	-4.05636	0.28232	-10.8259	2.6E-27	1.73E-25	Cluster 1
SAOUHSC_T00042		3921031			78.04391	3.595235	0.239838	10.82078	2.74E-27	1.78E-25	Cluster 5

SAOUHSC_02606	YP_501067.1	3921384	hutI	Imidazolonepropionase (EC 3.5.2.7) (Imidazolone-5-propionate hydrolase)	289.352	-3.42114	0.227298	-10.6519	1.71E-26	1.08E-24	Cluster 1
SAOUHSC_00204	YP_498801.1	3920360		Globin domain protein	158.2295	-3.3466	0.220709	-10.6321	2.11E-26	1.31E-24	Cluster 1
SAOUHSC_01022	YP_499571.1	3920282		Uncharacterized protein	322.3067	-4.11566	0.29636	-10.5131	7.52E-26	4.54E-24	Cluster 1
SAOUHSC_01166	YP_499706.1	3920918	pyrB	Aspartate carbamoyltransferase (EC 2.1.3.2) (Aspartate transcarbamylase) (ATCase)	214.4037	-4.2648	0.312828	-10.4364	1.69E-25	9.96E-24	Cluster 1
SAOUHSC_02388	YP_500865.1	3919605		Uncharacterized protein	282.6876	-3.4913	0.23904	-10.4221	1.97E-25	1.13E-23	Cluster 1
SAOUHSC_01172	YP_499711.1	3920923	pyrE	Orotate phosphoribosyltransferase (OPRT) (OPRTase) (EC 2.4.2.10)	241.2461	-3.57641	0.247738	-10.3997	2.49E-25	1.4E-23	Cluster 1
SAOUHSC_01063	YP_499609.1	3921725		Uncharacterized protein	295.2128	2.329648	0.129532	10.26498	1.01E-24	5.52E-23	Cluster 5
SAOUHSC_01877	YP_500380.1	3921766		Uncharacterized protein	140.2976	2.70296	0.165915	10.26407	1.02E-24	5.52E-23	Cluster 6
SAOUHSC_02675	YP_501137.1	3921237	nreC	Oxygen regulatory protein NreC (Nitrogen regulation protein C)	204.5879	-3.0372	0.204214	-9.97585	1.94E-23	1.03E-21	Cluster 1
SAOUHSC_T00039		3921165			44.51275	4.002961	0.303141	9.906138	3.91E-23	2.02E-21	Cluster 5
SAOUHSC_01902	YP_500403.1	3920849		Uncharacterized protein	71.18166	4.094818	0.314082	9.853528	6.62E-23	3.35E-21	Cluster 6
SAOUHSC_02683	YP_501145.1	3919702		Assimilatory nitrite reductase [NAD(P)H], small subunit, putative (EC 1.7.1.4)	65.4538	-6.09159	0.517147	-9.84553	7.17E-23	3.56E-21	Cluster 1
SAOUHSC_02677	YP_501139.1	3921239		Uncharacterized protein	247.2836	-3.18264	0.222044	-9.82978	8.38E-23	4.08E-21	Cluster 1
SAOUHSC_01041	YP_499590.1	3919889		Pyruvate dehydrogenase complex, E1 component, pyruvate dehydrogenase beta subunit, putative (EC 1.2.4.1)	2175.361	-2.64744	0.168609	-9.77074	1.5E-22	7.19E-21	Cluster 1
SAOUHSC_T00030		3921156			48.53235	3.534514	0.260112	9.743916	1.96E-22	9.19E-21	Cluster 5
SAOUHSC_01021	YP_499570.1	3920281		Uncharacterized protein	846.8312	-4.25643	0.335025	-9.71995	2.48E-22	1.14E-20	Cluster 1
SAOUHSC_00699	YP_499258.1	3921003		Deoxyribodipyrimidine photolyase, putative (EC 4.1.99.3)	387.2192	-2.49824	0.154247	-9.7132	2.65E-22	1.2E-20	Cluster 1
SAOUHSC_T00022		3921148			63.99702	3.155653	0.223883	9.628461	6.06E-22	2.7E-20	Cluster 5
SAOUHSC_02887	YP_501340.1	3921556	isaA	Probable transglycosylase IsaA (EC 3.2.-.-) (Immunodominant staphylococcal antigen A)	1074.424	3.872222	0.300908	9.545195	1.36E-21	5.94E-20	Cluster 5
SAOUHSC_02389	YP_500866.1	3919606		Cation efflux family protein, putative	889.8649	-3.50087	0.264573	-9.45247	3.31E-21	1.42E-19	Cluster 1
SAOUHSC_T00035		3921161			31.81945	3.797655	0.296499	9.435629	3.89E-21	1.64E-19	Cluster 5
SAOUHSC_02687	YP_501149.1	3919706		Formate/nitrite transporter, putative	172.637	-3.28552	0.243035	-9.40407	5.25E-21	2.18E-19	Cluster 1
SAOUHSC_00704	YP_499263.1	3921008		Uncharacterized protein	73.72626	3.105726	0.224298	9.388091	6.11E-21	2.46E-19	Cluster 6
SAOUHSC_01040	YP_499589.1	3919888	pdhA	Pyruvate dehydrogenase E1 component subunit alpha (EC 1.2.4.1)	2002.427	-2.51314	0.161178	-9.38804	6.11E-21	2.46E-19	Cluster 1
SAOUHSC_02830	YP_501289.1	3921269		D-lactate dehydrogenase, putative (EC 1.1.1.28)	665.0548	-4.41531	0.364324	-9.37436	6.96E-21	2.76E-19	Cluster 1
SAOUHSC_T00012		3921331			54.87241	3.232593	0.241159	9.257775	2.09E-20	8.14E-19	Cluster 5
SAOUHSC_02401	YP_500877.1	3919616		Uncharacterized protein	367.9559	-2.82336	0.197497	-9.23234	2.65E-20	1.02E-18	Cluster 1
SAOUHSC_01979	YP_500476.1	3920455		Uncharacterized protein	74.18777	4.156165	0.347575	9.080521	1.08E-19	4.09E-18	Cluster 5
SAOUHSC_00965	YP_499518.1	3920676		Uncharacterized protein	441.4207	2.169834	0.129105	9.061112	1.29E-19	4.81E-18	Cluster 5
SAOUHSC_00229	YP_498824.1	3920304	scdA	Iron-sulfur cluster repair protein ScdA (Cell wall-related protein ScdA)	83.45119	-3.16364	0.239161	-9.0468	1.47E-19	5.41E-18	Cluster 1
SAOUHSC_01173	YP_499712.1	3920924		Uncharacterized protein	86.11309	-3.23184	0.251294	-8.88141	6.6E-19	2.39E-17	Cluster 1
SAOUHSC_00907	YP_499460.1	3921753		UPF0344 protein SAOUHSC_00907	53.6114	2.873757	0.213792	8.764411	1.88E-18	6.7E-17	Cluster 6
SAOUHSC_T00032		3921158			95.21462	3.043755	0.234041	8.732483	2.49E-18	8.77E-17	Cluster 5
SAOUHSC_01878	YP_500381.1	3921767		Uncharacterized protein	81.38882	2.51522	0.17355	8.730715	2.53E-18	8.78E-17	Cluster 6
SAOUHSC_02134	YP_500624.1	3921204		Nitric oxide synthase oxygenase (EC 1.14.14.47)	185.2464	-2.40717	0.16132	-8.72283	2.71E-18	9.29E-17	Cluster 1
SAOUHSC_02682	YP_501144.1	3919701		Uroporphyrin-III C-methyltransferase, putative (EC 2.1.1.107)	121.8177	-4.47132	0.401237	-8.65154	5.08E-18	1.72E-16	Cluster 1
SAOUHSC_02468	YP_500936.1	3919031		Acetolactate synthase, putative (EC 2.2.1.6)	580.4931	-4.75429	0.436114	-8.60851	7.4E-18	2.47E-16	Cluster 1
SAOUHSC_02589	YP_501051.1	3921585		Uncharacterized protein	783.6085	-2.54649	0.181242	-8.53272	1.43E-17	4.7E-16	Cluster 1
SAOUHSC_01670	YP_500181.1	3920082		Cytidine deaminase (EC 3.5.4.5) (Cytidine aminohydrolase)	146.2681	-2.54578	0.181297	-8.52623	1.51E-17	4.91E-16	Cluster 1
SAOUHSC_02681	YP_501143.1	3921243		Nitrate reductase, alpha subunit (EC 1.7.99.4)	313.209	-4.55455	0.419967	-8.46388	2.59E-17	8.3E-16	Cluster 1
SAOUHSC_00540	YP_499112.1	3920820		Uncharacterized protein	34.75235	3.298368	0.274348	8.377572	5.4E-17	1.71E-15	Cluster 5
SAOUHSC_01789	YP_500294.1	3920431		Uncharacterized protein	319.8197	-3.21551	0.266964	-8.29893	1.05E-16	3.29E-15	Cluster 1
SAOUHSC_T0009		3921328			70.85236	3.259777	0.272974	8.278347	1.25E-16	3.86E-15	Cluster 5
SAOUHSC_01019	YP_499569.1	3920280		Uncharacterized protein	445.9257	-4.31597	0.40217	-8.24519	1.65E-16	5.03E-15	Cluster 1
SAOUHSC_T00033		3921159			85.13654	2.870564	0.227011	8.239968	1.72E-16	5.2E-15	Cluster 5
SAOUHSC_01158	YP_499698.1	3920716		Uncharacterized protein	422.666	1.903364	0.111325	8.114623	4.87E-16	1.45E-14	Cluster 5
SAOUHSC_01903	YP_500404.1	3920850	crcB1	Putative fluoride ion transporter CrcB 1	18.27616	3.621373	0.325298	8.05838	7.73E-16	2.28E-14	Cluster 6
SAOUHSC_02467	YP_500935.1	3919030		Alpha-acetolactate decarboxylase (EC 4.1.1.5)	296.6827	-4.93624	0.489254	-8.04539	8.6E-16	2.5E-14	Cluster 1
SAOUHSC_02708	YP_501170.1	3919727		Gamma-hemolysin h-gamma-ii subunit, putative	143.3926	-4.07796	0.384625	-8.00249	1.22E-15	3.51E-14	Cluster 1
SAOUHSC_T00055		3921044			79.92044	2.631763	0.206438	7.904368	2.69E-15	7.67E-14	Cluster 5
SAOUHSC_01042	YP_499591.1	3919890		Dihydrolipoamide acetyltransferase component of pyruvate dehydrogenase complex (EC 2.3.1.-)	2923.992	-2.34198	0.171272	-7.83537	4.67E-15	1.32E-13	Cluster 1
SAOUHSC_01895	YP_500396.1	3920842		Uncharacterized protein	502.7795	2.619933	0.209082	7.747844	9.35E-15	2.6E-13	Cluster 5
SAOUHSC_T00053		3921042			37.64932	3.021271	0.262652	7.695611	1.41E-14	3.88E-13	Cluster 5
SAOUHSC_02546	YP_501009.1	3921135		Molybdenum transport ATP-binding protein ModC, putative	170.0459	-2.42222	0.186365	-7.63136	2.32E-14	6.33E-13	Cluster 1
SAOUHSC_T00051		3921040			92.05049	3.667413	0.350632	7.607446	2.8E-14	7.54E-13	Cluster 5

SAOUHSC_01886	YP_500388.1	3920834	ribH	6,7-dimethyl-8-ribityllumazine synthase (DMRL synthase) (LS) (Lumazine synthase) (EC 2.5.1.78)	92.42023	3.038154	0.269962	7.549788	4.36E-14	1.16E-12	Cluster 6
SAOUHSC_02573	YP_501034.1	3921570		Na+/H+ antiporter NhaC	199.3228	2.257298	0.166905	7.533026	4.96E-14	1.31E-12	Cluster 6
SAOUHSC_00420	YP_499005.1	3920577		Transporter	187.1314	3.253123	0.29935	7.526709	5.2E-14	1.36E-12	Cluster 5
SAOUHSC_02709	YP_501171.1	3919728	hlgC	Gamma-hemolysin component C (Leukocidin s subunit)	177.2058	-4.02565	0.403129	-7.50542	6.12E-14	1.58E-12	Cluster 1
SAOUHSC_02006	YP_500503.1	3921886		Uncharacterized protein	416.1939	-2.05415	0.140788	-7.48749	7.02E-14	1.78E-12	Cluster 1
SAOUHSC_02770	YP_501229.1	3921425		Uncharacterized protein	26.3034	3.862765	0.382309	7.488094	6.99E-14	1.78E-12	Cluster 5
SAOUHSC_02599	YP_501060.1	3921594		Hex regulon repressor, putative	28.54074	3.470416	0.330166	7.482352	7.3E-14	1.83E-12	Cluster 6
SAOUHSC_T00026		3921152			98.20923	3.181562	0.292479	7.458856	8.73E-14	2.17E-12	Cluster 5
SAOUHSC_02541	YP_501004.1	3921130		Molybdopterin-guanine dinucleotide biosynthesis protein MobB	141.2267	-2.31935	0.177754	-7.42234	1.15E-13	2.83E-12	Cluster 1
SAOUHSC_00329	YP_498918.1	3919594	tatA	Sec-independent protein translocase protein TatA	56.98184	3.059576	0.278999	7.382012	1.56E-13	3.8E-12	Cluster 6
SAOUHSC_01222	YP_499758.1	3920250	topA	DNA topoisomerase 1 (EC 5.6.2.2) (DNA topoisomerase I) (Omega-protein) (Relaxing enzyme) (Swivelase) (Untwisting enzyme)	610.9216	3.03806	0.277338	7.348654	2E-13	4.83E-12	Cluster 5
SAOUHSC_03047	YP_501493.1	3921311		Uncharacterized protein	33.60685	-4.31494	0.453864	-7.30381	2.8E-13	6.69E-12	Cluster 1
SAOUHSC_01615	YP_500130.1	3920031		DNA repair protein RecN (Recombination protein N)	450.9667	-1.89527	0.124234	-7.20631	5.75E-13	1.36E-11	Cluster 1
SAOUHSC_00019	YP_498625.1	3919190	purA	Adenylosuccinate synthetase (AMPSase) (AdSS) (EC 6.3.4.4) (IMP--aspartate ligase)	149.2971	2.653474	0.230866	7.162037	7.95E-13	1.86E-11	Cluster 6
SAOUHSC_00367	YP_498956.1	3919786		Uncharacterized protein	345.0306	-2.0267	0.144302	-7.11495	1.12E-12	2.6E-11	Cluster 1
SAOUHSC_00317	YP_498907.1	3919544		Glycerol-3-phosphate transporter	150.8176	3.115128	0.298119	7.094911	1.29E-12	2.98E-11	Cluster 6
SAOUHSC_00975	YP_499528.1	3920120		Uncharacterized protein	41.3498	3.541372	0.35911	7.076865	1.47E-12	3.37E-11	Cluster 6
SAOUHSC_T00045		3921034			84.24058	3.342979	0.333904	7.016934	2.27E-12	5.13E-11	Cluster 5
SAOUHSC_02542	YP_501005.1	3921131		Molybdopterin molybdenumtransferase (EC 2.10.1.1)	219.4669	-2.3306	0.189664	-7.01557	2.29E-12	5.14E-11	Cluster 1
SAOUHSC_02909	YP_501362.1	3921361		Dihydroorotate dehydrogenase (quinone) (EC 1.3.5.2)	123.9519	-2.28343	0.183717	-6.98592	2.83E-12	6.29E-11	Cluster 1
SAOUHSC_02855	YP_501311.1	3921527		LysM domain protein (EC 3.5.1.28)	56.18043	4.527356	0.506451	6.964854	3.29E-12	7.24E-11	Cluster 6
SAOUHSC_T00050		3921039			7.528816	6.173333	0.744765	6.946261	3.75E-12	8.19E-11	Cluster 6
SAOUHSC_02595	YP_501057.1	3921591		Uncharacterized protein	148.3689	4.374359	0.487451	6.922454	4.44E-12	9.61E-11	Cluster 6
SAOUHSC_02744	YP_501205.1	3921618		Amino acid ABC transporter, ATP-binding protein, putative	106.085	-3.02915	0.295863	-6.85841	6.96E-12	1.5E-10	Cluster 1
SAOUHSC_02930	YP_501383.1	3921637		Uncharacterized protein	92.40008	-2.38893	0.203978	-6.80923	9.81E-12	2.09E-10	Cluster 1
SAOUHSC_01043	YP_499592.1	3919891		Dihydrolipoyl dehydrogenase (EC 1.8.1.4)	2962.782	-2.18721	0.174511	-6.80305	1.02E-11	2.16E-10	Cluster 1
SAOUHSC_02877	YP_501332.1	3921548	crtN	4,4'-diapophytoene desaturase (4,4'-diaponeurosporene-forming) (EC 1.3.8.-) (Dehydrosqualene desaturase)	339.0115	-2.24633	0.183255	-6.80108	1.04E-11	2.17E-10	Cluster 1
SAOUHSC_03046	YP_501492.1	3921310		Helix-turn-helix domain protein	48.04547	-3.21144	0.326306	-6.77721	1.23E-11	2.54E-10	Cluster 1
SAOUHSC_00769	YP_499326.1	3919060	secA1	Protein translocase subunit SecA 1	1665.172	-1.84706	0.125064	-6.77298	1.26E-11	2.6E-10	Cluster 1
SAOUHSC_T00060		3921068			14.71875	3.975746	0.441428	6.741178	1.57E-11	3.21E-10	Cluster 5
SAOUHSC_T0007		3921326			55.89781	3.253097	0.338154	6.662942	2.68E-11	5.44E-10	Cluster 5
SAOUHSC_00203	YP_498800.1	3920359		Uncharacterized protein	44.86127	-3.03299	0.30645	-6.63399	3.27E-11	6.57E-10	Cluster 1
SAOUHSC_01058	YP_499605.1	3921721		GTP-binding protein TypA, putative	375.8979	3.117104	0.319427	6.627818	3.41E-11	6.8E-10	Cluster 5
SAOUHSC_00100	YP_498700.1	3919809	deoC	Deoxyribose-phosphate aldolase (DERA) (EC 4.1.2.4) (2-deoxy-D-ribose 5-phosphate aldolase) (Phosphodeoxyriboaldolase) (Deoxyriboaldolase)	381.4704	-2.00024	0.151864	-6.58637	4.51E-11	8.92E-10	Cluster 1
SAOUHSC_T00014		3921333			66.79372	3.158929	0.329494	6.552252	5.67E-11	1.11E-09	Cluster 5
SAOUHSC_T00024		3921150			73.98496	3.021086	0.310128	6.516933	7.18E-11	1.4E-09	Cluster 5
SAOUHSC_T00043		3921032			40.74094	3.015228	0.309601	6.509106	7.56E-11	1.46E-09	Cluster 5
SAOUHSC_00281	YP_498873.1	3918965		Uncharacterized protein	60.643	-2.86349	0.287753	-6.47601	9.42E-11	1.81E-09	Cluster 1
SAOUHSC_02891	YP_501344.1	3921343		Uncharacterized protein	56.30797	2.810062	0.28001	6.464286	1.02E-10	1.94E-09	Cluster 6
SAOUHSC_T00059		3921067			14.12524	3.671278	0.41565	6.426746	1.3E-10	2.47E-09	Cluster 5
SAOUHSC_02611	YP_501072.1	3921389	lyrA	Lysostaphin resistance protein A	203.5449	2.304774	0.204385	6.383915	1.73E-10	3.24E-09	Cluster 6
SAOUHSC_02768	YP_501227.1	3921423		Uncharacterized protein	20.4983	3.807209	0.443002	6.336785	2.35E-10	4.37E-09	Cluster 5
SAOUHSC_00328	YP_498917.1	3919593	tatC	Sec-independent protein translocase protein TatC	80.76953	2.958348	0.309333	6.33087	2.44E-10	4.51E-09	Cluster 6
SAOUHSC_02587	YP_501049.1	3921583		Uncharacterized protein	52.40302	2.631317	0.257724	6.3297	2.46E-10	4.51E-09	Cluster 5
SAOUHSC_01671	YP_500182.1	3920083		Diacylglycerol kinase, putative (EC 2.7.1.107)	119.9807	-2.28921	0.203766	-6.32689	2.5E-10	4.56E-09	Cluster 1
SAOUHSC_02607	YP_501068.1	3921385	hutU	Urocanate hydratase (Urocanase) (EC 4.2.1.49) (Imidazolonepropionate hydrolase)	375.1239	-3.46891	0.391081	-6.31305	2.74E-10	4.95E-09	Cluster 1
SAOUHSC_02879	YP_501333.1	3921549	crtM	4,4'-diapophytoene synthase (DAP synthase) (EC 2.5.1.96) (C30 carotenoid synthase) (Dehydrosqualene synthase)	193.0345	-2.09665	0.175584	-6.24572	4.22E-10	7.58E-09	Cluster 1
SAOUHSC_01978	YP_500475.1	3920454		UPF0754 membrane protein SAOUHSC_01978	111.0364	2.332065	0.218663	6.091863	1.12E-09	1.99E-08	Cluster 6
SAOUHSC_00888	YP_499441.1	3921734	mnhB1	Na(+)/H(+) antiporter subunit B1 (Mnh complex subunit B1)	359.091	-2.00542	0.165944	-6.05879	1.37E-09	2.43E-08	Cluster 1
SAOUHSC_02097	YP_500588.1	3921169		Uncharacterized protein	428.9274	2.578982	0.262942	6.005047	1.91E-09	3.37E-08	Cluster 6
SAOUHSC_00351	YP_498940.1	3921824		Uncharacterized protein	27.31579	2.486454	0.249856	5.949243	2.69E-09	4.68E-08	Cluster 6
SAOUHSC_02812	YP_501270.1	3921251		Uncharacterized protein	393.002	2.099357	0.184758	5.950258	2.68E-09	4.68E-08	Cluster 5

SAOUHSC_00535	YP_499107.1	3920815		Uncharacterized epimerase/dehydratase SAOUHSC_00535	601.6013	-2.70053	0.285962	-5.94672	2.74E-09	4.72E-08	Cluster 3
SAOUHSC_01425	YP_499951.1	3920656		Uncharacterized protein	43.27902	2.388312	0.234704	5.915164	3.32E-09	5.68E-08	Cluster 6
SAOUHSC_01156	YP_499697.1	3920715		Uncharacterized protein	208.6076	1.99874	0.169097	5.906305	3.5E-09	5.95E-08	Cluster 5
SAOUHSC_01084	YP_499629.1	3919245		Uncharacterized protein	51.03169	3.973524	0.507018	5.864736	4.5E-09	7.6E-08	Cluster 5
SAOUHSC_00922	YP_499475.1	3920809		Uncharacterized protein	9.879163	3.675273	0.456747	5.857228	4.71E-09	7.9E-08	Cluster 6
SAOUHSC_T00019		3921338			54.27865	2.876472	0.320697	5.851227	4.88E-09	8.13E-08	Cluster 5
SAOUHSC_00101	YP_498701.1	3919810	deoB	Phosphopentomutase (EC 5.4.2.7) (Phosphodeoxyribomutase)	723.7071	-2.01032	0.173064	-5.83782	5.29E-09	8.76E-08	Cluster 1
SAOUHSC_00964	YP_499517.1	3920675		Uncharacterized protein	187.2078	2.057912	0.181251	5.836735	5.32E-09	8.76E-08	Cluster 5
SAOUHSC_01354	YP_499882.1	3920060		Sodium:alanine symporter family protein, putative	400.0455	1.767362	0.131609	5.830619	5.52E-09	9.03E-08	Cluster 6
SAOUHSC_00995	YP_499547.1	3920395		Putative acetyltransferase SAOUHSC_00995 (EC 2.3.1.-) (GCN5-related N-acetyltransferase) (GNAT)	29.94159	2.583629	0.271834	5.825723	5.69E-09	9.24E-08	Cluster 6
SAOUHSC_01667	YP_500178.1	3920079	recO	DNA repair protein RecO (Recombination protein O)	192.9427	-1.97314	0.167324	-5.81593	6.03E-09	9.73E-08	Cluster 1
SAOUHSC_02889	YP_501342.1	3921341		Uncharacterized protein	16.05835	4.108609	0.534655	5.814236	6.09E-09	9.77E-08	Cluster 6
SAOUHSC_00863	YP_499416.1	3918993		Uncharacterized protein	21.19809	2.758788	0.303306	5.79873	6.68E-09	1.06E-07	Cluster 5
SAOUHSC_00548	YP_499120.1	3920827		Uncharacterized protein	368.1979	-2.10094	0.191557	-5.74732	9.07E-09	1.44E-07	Cluster 1
SAOUHSC_02631	YP_501092.1	3921408		Uncharacterized protein	64.64795	3.019588	0.35166	5.743021	9.3E-09	1.46E-07	Cluster 5
SAOUHSC_01085	YP_499630.1	3919246		conserved hypothetical protein	35.98297	3.632276	0.458505	5.740995	9.41E-09	1.47E-07	Cluster 6
SAOUHSC_02883	YP_501337.1	3921553	ssaA	Staphylococcal secretory antigen SsaA	82.82713	4.890637	0.678773	5.73187	9.93E-09	1.54E-07	Cluster 6
SAOUHSC_01832	YP_500338.1	3921782		Uncharacterized protein	681.6078	3.779674	0.485351	5.727136	1.02E-08	1.58E-07	Cluster 6
SAOUHSC_02982	YP_501431.1	3921464	sasF	Surface protein F	461.228	-1.88351	0.154809	-5.70711	1.15E-08	1.76E-07	Cluster 1
SAOUHSC_00884	YP_499437.1	3919231	mnhF1	Na(+)/H(+) antiporter subunit F1 (Mnh complex subunit F1)	255.9598	-2.06248	0.186472	-5.69782	1.21E-08	1.85E-07	Cluster 1
SAOUHSC_02622	YP_501083.1	3921399		Sodium/glutamate symporter	110.8752	3.15775	0.381634	5.653973	1.57E-08	2.38E-07	Cluster 6
SAOUHSC_01389	YP_499916.1	3920680	pstS	Phosphate-binding protein PstS (PBP)	42.29842	4.309627	0.58979	5.611531	2.01E-08	3.02E-07	Cluster 4
SAOUHSC_01971	YP_500468.1	3920447		Uncharacterized protein	29.86994	2.528679	0.272506	5.609709	2.03E-08	3.04E-07	Cluster 6
SAOUHSC_02689	YP_501151.1	3919708		Uncharacterized protein	18.92693	4.043908	0.543167	5.603997	2.09E-08	3.12E-07	Cluster 6
SAOUHSC_00885	YP_499438.1	3919232	mnhE1	Na(+)/H(+) antiporter subunit E1 (Mnh complex subunit E1)	399.9646	-1.98586	0.176079	-5.59898	2.16E-08	3.17E-07	Cluster 1
SAOUHSC_01985	YP_500482.1	3920461		Uncharacterized protein	21.25439	2.693158	0.302369	5.599648	2.15E-08	3.17E-07	Cluster 6
SAOUHSC_02265	YP_500746.1	3919140		Accessory gene regulator protein A	1575.905	-2.63318	0.291736	-5.59817	2.17E-08	3.17E-07	Cluster 1
SAOUHSC_00949	YP_499502.1	3920660		Uncharacterized protein	451.5949	3.834348	0.507183	5.588413	2.29E-08	3.34E-07	Cluster 6
SAOUHSC_01164	YP_499704.1	3920916	pyrR	Bifunctional protein PyrR [Includes: Pyrimidine operon regulatory protein; Uracil phosphoribosyltransferase (UPRTase) (EC 2.4.2.9)]	139.7772	-2.36338	0.244911	-5.56684	2.59E-08	3.76E-07	Cluster 1
SAOUHSC_01833	YP_500339.1	3921783		D-3-phosphoglycerate dehydrogenase (EC 1.1.1.95)	939.8025	3.839045	0.5103	5.563486	2.64E-08	3.81E-07	Cluster 6
SAOUHSC_00555	YP_499127.1	3920600		Hydrolase, haloacid dehalogenase-like	41.22971	2.387104	0.249764	5.553656	2.8E-08	4.01E-07	Cluster 6
SAOUHSC_00060	YP_498664.1	3919091		Uncharacterized protein	259.1687	2.532827	0.27659	5.541872	2.99E-08	4.26E-07	Cluster 5
SAOUHSC_02769	YP_501228.1	3921424		Uncharacterized protein	15.89639	3.901013	0.52358	5.540729	3.01E-08	4.26E-07	Cluster 5
SAOUHSC_T00036		3921162			59.41984	2.584355	0.287232	5.515947	3.47E-08	4.88E-07	Cluster 5
SAOUHSC_01668	YP_500179.2	3920080	era	GTPase Era	260.2635	-2.24243	0.225456	-5.51077	3.57E-08	5E-07	Cluster 1
SAOUHSC_01634	YP_500147.1	3919971	gcvT	Aminomethyltransferase (EC 2.1.2.10) (Glycine cleavage system T protein)	165.9732	2.923183	0.349566	5.501634	3.76E-08	5.24E-07	Cluster 6
SAOUHSC_02241	YP_500725.1	3919663		Uncharacterized leukocidin-like protein 1	218.8248	-3.88152	0.527399	-5.46364	4.66E-08	6.46E-07	Cluster 1
SAOUHSC_01121	YP_499665.1	3920722		alpha-hemolysin precursor	497.7348	-2.84099	0.337646	-5.45243	4.97E-08	6.84E-07	Cluster 3
SAOUHSC_T00034		3921160			11.079	4.640936	0.669459	5.438622	5.37E-08	7.35E-07	Cluster 5
SAOUHSC_02737	YP_501198.1	3921611		Epimerase/dehydratase, putative	102.4015	1.959593	0.176612	5.433325	5.53E-08	7.54E-07	Cluster 5
SAOUHSC_00698	YP_499257.1	3921002		Uncharacterized protein	206.7331	-1.75742	0.140432	-5.39346	6.91E-08	9.37E-07	Cluster 1
SAOUHSC_00064	YP_498667.1	3919094	norG	HTH-type transcriptional regulator NorG	9.753038	3.214639	0.410994	5.388495	7.11E-08	9.58E-07	Cluster 6
SAOUHSC_01980	YP_500477.1	3920456		DNA-binding response regulator, putative	43.23544	2.170489	0.217309	5.386285	7.19E-08	9.64E-07	Cluster 5
SAOUHSC_02294	YP_500773.1	3919166		Uncharacterized protein	10.55147	4.826092	0.710683	5.383683	7.3E-08	9.73E-07	Cluster 5
SAOUHSC_00113	YP_498713.1	3919822		Aldehyde-alcohol dehydrogenase	366.8791	-3.76883	0.515619	-5.36991	7.88E-08	1.05E-06	Cluster 1
SAOUHSC_02547	YP_501010.1	3921136		Molybdenum transport system permease	232.9604	-2.42873	0.266966	-5.35173	8.71E-08	1.15E-06	Cluster 1
SAOUHSC_02328	YP_500807.1	3920953	thiE	Thiamine-phosphate synthase (TP synthase) (TPS) (EC 2.5.1.3) (Thiamine-phosphate pyrophosphorylase) (TMP pyrophosphorylase) (TMP-PPase)	48.0557	-3.83579	0.534803	-5.3025	1.14E-07	1.5E-06	Cluster 1
SAOUHSC_00202	YP_498799.1	3920358		Uncharacterized protein	24.90117	-2.89012	0.357116	-5.29272	1.21E-07	1.57E-06	Cluster 1
SAOUHSC_02785	YP_501245.1	3921440		Uncharacterized protein	79.11951	-2.17277	0.223168	-5.25511	1.48E-07	1.92E-06	Cluster 1
SAOUHSC_02985	YP_501434.1	3921467	secA2	Protein translocase subunit SecA 2	463.8983	-2.3609	0.260861	-5.21694	1.82E-07	2.35E-06	Cluster 1
SAOUHSC_02424	YP_500895.1	3919634		Uncharacterized protein	228.5306	-1.92031	0.176746	-5.20696	1.92E-07	2.47E-06	Cluster 1
SAOUHSC_00401	YP_498988.1	3919136		Uncharacterized protein	22.33275	3.226175	0.434887	5.118975	3.07E-07	3.93E-06	Cluster 6
SAOUHSC_02264	YP_500745.1	3919139		Accessory gene regulator protein C	1509.631	-2.58855	0.310406	-5.11764	3.09E-07	3.94E-06	Cluster 1

SAOUHSC_02984	YP_501433.1	3921466	gtfA	UDP-N-acetylglucosamine--peptide N-acetylglucosaminyltransferase GtfA subunit (EC 2.4.1.-) (Glycosyltransferase GtfA)	444.1007	-2.10284	0.21623	-5.10033	3.39E-07	4.3E-06	Cluster 1
SAOUHSC_00899	YP_499452.1	3921745	argG	Argininosuccinate synthase (EC 6.3.4.5) (Citrulline--aspartate ligase)	475.9187	2.116959	0.219343	5.092303	3.54E-07	4.46E-06	Cluster 4
SAOUHSC_01424	YP_499950.1	3920655	murG	UDP-N-acetylglucosamine--N-acetylmuramyl-(pentapeptide) pyrophosphoryl-undecaprenol N-acetylglucosamine transferase (EC 2.4.1.227) (Undecaprenyl-PP-MurNAc-pentapeptide-UDPGlcNAc GlcNAc transferase)	140.8253	2.028289	0.202189	5.085771	3.66E-07	4.59E-06	Cluster 6
SAOUHSC_00289	YP_498879.1	3918970		Uncharacterized protein	30.94812	4.221287	0.634643	5.075748	3.86E-07	4.82E-06	Cluster 6
SAOUHSC_01361	YP_499888.1	3920066	msrR	Regulatory protein MsrR	154.6053	2.285079	0.254061	5.058157	4.23E-07	5.26E-06	Cluster 5
SAOUHSC_02069	YP_500562.1	3919756		Phi PV83 orf 20-like protein	13.03755	5.620444	0.920404	5.020018	5.17E-07	6.39E-06	Cluster 5
SAOUHSC_02255	YP_500737.1	3919675	groES	10 kDa chaperonin Cpn10 GroES forms homoheptameric ring binds to one or both ends of the GroEL double barrel in the presence of adenine nucleotides capping it folding of unfolded substrates initiates in a GroEL-substrate bound and capped by GroES release of the folded substrate is dependent on ATP binding and hydrolysis in the trans ring	1099.208	3.277078	0.457839	4.973531	6.57E-07	8.09E-06	Cluster 4
SAOUHSC_T00044		3921033			17.7156	3.551735	0.513529	4.969021	6.73E-07	8.24E-06	Cluster 6
SAOUHSC_01633	YP_500146.1	3919970	gcvPA	Probable glycine dehydrogenase (decarboxylating) subunit 1 (EC 1.4.4.2) (Glycine cleavage system P-protein subunit 1) (Glycine decarboxylase subunit 1) (Glycine dehydrogenase (aminomethyl-transferring) subunit 1)	268.7589	2.103451	0.223898	4.928355	8.29E-07	1.01E-05	Cluster 6
SAOUHSC_00887	YP_499440.1	3919234	mnhC1	Na(+)/H(+) antiporter subunit C1 (Mnh complex subunit C1)	327.4822	-1.9769	0.198959	-4.91008	9.1E-07	1.1E-05	Cluster 1
SAOUHSC_01088	YP_499633.1	3919249	srtB	Sortase B (EC 3.4.22.71)	18.4419	3.506308	0.510505	4.909471	9.13E-07	1.1E-05	Cluster 5
SAOUHSC_02520	YP_500985.1	3921111		Sugar transporter, putative	162.3337	1.718792	0.146565	4.904259	9.38E-07	1.13E-05	Cluster 6
SAOUHSC_01650	YP_500162.1	3920102		5-formyltetrahydrofolate cyclo-ligase (EC 6.3.3.2)	102.2419	-2.21451	0.248251	-4.89228	9.97E-07	1.19E-05	Cluster 1
SAOUHSC_03015	YP_501464.1	3921496	hisZ	ATP phosphoribosyltransferase regulatory subunit	97.88658	3.200668	0.454427	4.842735	1.28E-06	1.52E-05	Cluster 4
SAOUHSC_00434	YP_499017.1	3919106		Transcriptional regulator, lysR family, putative	27.48046	2.38387	0.289953	4.772744	1.82E-06	2.15E-05	Cluster 5
SAOUHSC_01689	YP_500199.1	3921801	rpsT	30S ribosomal protein S20	114.0437	2.021952	0.214563	4.76294	1.91E-06	2.25E-05	Cluster 5
SAOUHSC_01082	YP_499628.1	3919244		conserved hypothetical protein	36.0948	3.456652	0.519682	4.727227	2.28E-06	2.66E-05	Cluster 5
SAOUHSC_T00052		3921041			127.1458	2.781147	0.376813	4.726873	2.28E-06	2.66E-05	Cluster 5
SAOUHSC_T0004		3921323			29.02166	3.118392	0.449407	4.713749	2.43E-06	2.83E-05	Cluster 5
SAOUHSC_01081	YP_499627.1	3919243		conserved hypothetical protein	77.74226	3.604501	0.557556	4.671282	2.99E-06	3.46E-05	Cluster 6
SAOUHSC_R0004		3921504			55386.14	3.515172	0.539545	4.661649	3.14E-06	3.6E-05	Cluster 4
SAOUHSC_02540	YP_501003.1	3921129		Molybdopterin converting factor moa, putative	155.1243	-2.11442	0.239073	-4.66143	3.14E-06	3.6E-05	Cluster 1
SAOUHSC_02011	YP_500508.1	3920465	recX	Regulatory protein RecX	47.59751	1.994785	0.213468	4.66012	3.16E-06	3.61E-05	Cluster 6
SAOUHSC_02243	YP_500726.1	3919664		Uncharacterized leukocidin-like protein 2	161.0216	-3.3662	0.509162	-4.64725	3.36E-06	3.82E-05	Cluster 1
SAOUHSC_00158	YP_498757.1	3919866		PTS system EIIBC component SAOUHSC_00158 [Includes: Phosphotransferase enzyme IIB component (EC 2.7.1.-) (PTS system EIIB component); Permease IIC component (PTS system EIIC component)]	182.4253	-2.10899	0.241509	-4.59194	4.39E-06	4.97E-05	Cluster 1
SAOUHSC_00905	YP_499458.1	3921751	addA	ATP-dependent helicase/nuclease subunit A (EC 3.1.-.-) (EC 3.6.4.12) (ATP-dependent helicase/nuclease AddA)	567.3458	-1.56557	0.123437	-4.58182	4.61E-06	5.19E-05	Cluster 1
SAOUHSC_02135	YP_500625.1	3921832		Uncharacterized protein	135.8131	-2.13514	0.24784	-4.58011	4.65E-06	5.21E-05	Cluster 1
SAOUHSC_01504	YP_500022.1	3919047		Ferredoxin, putative	163.4734	3.52836	0.55377	4.565723	4.98E-06	5.56E-05	Cluster 5
SAOUHSC_01319	YP_499849.1	3920145		Aspartokinase (EC 2.7.2.4)	285.625	1.574545	0.126313	4.548583	5.4E-06	6E-05	Cluster 6
SAOUHSC_01330	YP_499860.1	3920195	guaC	GMP reductase (EC 1.7.1.7) (Guanosine 5'-monophosphate oxidoreductase) (Guanosine monophosphate reductase)	102.6157	3.08843	0.461332	4.52696	5.98E-06	6.62E-05	Cluster 5
SAOUHSC_R0001		3921501			49146.55	3.485672	0.549627	4.522469	6.11E-06	6.73E-05	Cluster 4
SAOUHSC_02574	YP_501035.1	3921571		Uncharacterized protein	162.3062	2.202316	0.266593	4.509932	6.48E-06	7.11E-05	Cluster 6
SAOUHSC_01853	YP_500357.1	3920531		Uncharacterized protein	6.149828	3.708832	0.601297	4.504979	6.64E-06	7.25E-05	Cluster 6
SAOUHSC_T00041		3921030			102.1107	2.766926	0.394111	4.483319	7.35E-06	7.99E-05	Cluster 5
SAOUHSC_02881	YP_501335.1	3921551		conserved hypothetical protein	312.1886	-2.01787	0.227374	-4.47661	7.58E-06	8.21E-05	Cluster 2
SAOUHSC_01861	YP_500365.1	3920539		Uncharacterized protein	301.6388	-1.65367	0.146762	-4.45396	8.43E-06	9.09E-05	Cluster 3
SAOUHSC_02924	YP_501377.1	3921375		Uncharacterized protein	456.9276	3.277536	0.511944	4.448803	8.64E-06	9.27E-05	Cluster 6
SAOUHSC_02465	YP_500933.1	3919028		Uncharacterized hydrolase SAOUHSC_02465 (EC 3.-.-.-)	50.17264	-2.10021	0.247489	-4.44549	8.77E-06	9.38E-05	Cluster 1
SAOUHSC_02888	YP_501341.1	3921557		Uncharacterized protein	516.0139	3.395316	0.53925	4.441936	8.92E-06	9.49E-05	Cluster 6
SAOUHSC_00913	YP_499466.1	3920800		Uncharacterized protein	26.48186	3.827044	0.637285	4.436076	9.16E-06	9.71E-05	Cluster 5
SAOUHSC_01984	YP_500481.1	3920460		Uncharacterized protein	37.27694	2.073608	0.242583	4.425739	9.61E-06	0.000101	Cluster 6
SAOUHSC_01009	YP_499559.1	3920270	purK	N5-carboxyaminoimidazole ribonucleotide synthase (N5-CAIR synthase) (EC 6.3.4.18) (5-(carboxyamino)imidazole ribonucleotide synthetase)	75.80218	2.2514	0.284648	4.396307	1.1E-05	0.000115	Cluster 5

SAOUHSC_01672	YP_500183.1	3920084	ybeY	Endoribonuclease YbeY (EC 3.1.-.-)	167.8368	-2.10563	0.251498	-4.39617	1.1E-05	0.000115	Cluster 1
SAOUHSC_00075	YP_498675.1	3919453	sbnA	N-(2-amino-2-carboxyethyl)-L-glutamate synthase (ACEGA synthase) (EC 2.5.1.140) (Staphyloferrin B biosynthesis protein SbnA)	10.83604	3.605014	0.593004	4.392915	1.12E-05	0.000117	Cluster 6
SAOUHSC_00156	YP_498755.1	3919864		Uncharacterized protein	102.0609	-2.19034	0.271115	-4.39053	1.13E-05	0.000117	Cluster 1
SAOUHSC_00500	YP_499073.1	3920412	pdxT	Pyridoxal 5'-phosphate synthase subunit PdxT (EC 4.3.3.6) (Pdx2) (Pyridoxal 5'-phosphate synthase glutaminase subunit) (EC 3.5.1.2)	370.0935	-1.72131	0.164509	-4.38464	1.16E-05	0.00012	Cluster 1
SAOUHSC_T0002		3921518			105.5966	2.587658	0.362294	4.382234	1.17E-05	0.000121	Cluster 5
SAOUHSC_R0002		3921502			52568.76	3.388974	0.545607	4.378561	1.19E-05	0.000123	Cluster 4
SAOUHSC_01394	YP_499921.1	3920684		Aspartokinase (EC 2.7.2.4)	1244.68	2.866636	0.427042	4.371082	1.24E-05	0.000126	Cluster 4
SAOUHSC_R0003		3921503			49318.75	3.387452	0.54696	4.36495	1.27E-05	0.000129	Cluster 4
SAOUHSC_02432	YP_500901.1	3919640		Uncharacterized protein	3.431409	3.781112	0.637934	4.359559	1.3E-05	0.000132	Cluster 6
SAOUHSC_R0005		3921505			47135.17	3.396704	0.550489	4.35377	1.34E-05	0.000135	Cluster 4
SAOUHSC_01013	YP_499563.1	3920274	purL	Phosphoribosylformylglycinamidine synthase subunit PurL (FGAM synthase) (EC 6.3.5.3) (Formylglycinamide ribonucleotide amidotransferase subunit II) (FGAR amidotransferase II) (FGAR-AT II) (Glutamine amidotransferase PurL) (Phosphoribosylformylglycinamidine synthase subunit II)	148.2964	1.91198	0.209581	4.351441	1.35E-05	0.000136	Cluster 6
SAOUHSC_02464	YP_500932.1	3919027		Uncharacterized protein	48.09426	-2.06332	0.244946	-4.34105	1.42E-05	0.000142	Cluster 1
SAOUHSC_01399	YP_499926.1	3920689		Uncharacterized hydrolase SAOUHSC_01399 (EC 3.-.-.-)	534.9038	2.685814	0.388934	4.334444	1.46E-05	0.000146	Cluster 4
SAOUHSC_02422	YP_500893.1	3919632		Uncharacterized protein	365.4118	-1.63553	0.146957	-4.32457	1.53E-05	0.000152	Cluster 1
SAOUHSC_02536	YP_501000.1	3921126	moaA	GTP 3',8-cyclase (EC 4.1.99.22) (Molybdenum cofactor biosynthesis protein A)	335.0625	-1.74058	0.171845	-4.30957	1.64E-05	0.000162	Cluster 1
SAOUHSC_00209	YP_498805.1	3920364		PTS system, glucose-specific IIBC component, putative (EC 2.7.1.69)	114.7496	-2.37264	0.320495	-4.28288	1.84E-05	0.000182	Cluster 1
SAOUHSC_00868	YP_499421.2	3918998		Uncharacterized protein	75.46246	1.812047	0.18985	4.277303	1.89E-05	0.000186	Cluster 6
SAOUHSC_03014	YP_501463.1	3921495	hisG	ATP phosphoribosyltransferase (ATP-PRT) (ATP-PRTase) (EC 2.4.2.17)	54.6809	2.8581	0.437131	4.250667	2.13E-05	0.000209	Cluster 4
SAOUHSC_00051	YP_498656.1	3919083	plc	1-phosphatidylinositol phosphodiesterase (EC 4.6.1.13) (Phosphatidylinositol diacylglycerol-lyase) (Phosphatidylinositol-specific phospholipase C) (PI-PLC)	23.11514	-2.68748	0.399055	-4.22868	2.35E-05	0.000229	Cluster 1
SAOUHSC_01893	YP_500394.1	3920840		Arsenical pump membrane protein	80.27411	2.928534	0.456622	4.22348	2.41E-05	0.000234	Cluster 5
SAOUHSC_00694	YP_499253.1	3920998		conserved hypothetical protein	1498.578	1.370749	0.087991	4.213463	2.51E-05	0.000242	Cluster 5
SAOUHSC_02266	YP_500747.1	3919141		Uncharacterized protein	49.80378	-2.37814	0.327071	-4.21359	2.51E-05	0.000242	Cluster 1
SAOUHSC_00372	YP_498961.1	3919791	xpt	Xanthine phosphoribosyltransferase (XPRTase) (EC 2.4.2.22)	201.0971	2.928174	0.457997	4.210015	2.55E-05	0.000245	Cluster 6
SAOUHSC_00867	YP_499420.1	3918997		Uncharacterized protein	39.94071	1.859315	0.204197	4.208263	2.57E-05	0.000246	Cluster 6
SAOUHSC_01590	YP_500105.1	3920006		Uncharacterized protein	28.3666	2.847886	0.440351	4.196398	2.71E-05	0.000258	Cluster 5
SAOUHSC_02647	YP_501109.1	3921209	mqo	Probable malate:quinone oxidoreductase (EC 1.1.5.4) (MQO) (Malate dehydrogenase [quinone])	419.9405	-2.1336	0.270156	-4.1961	2.72E-05	0.000258	Cluster 1
SAOUHSC_02784	YP_501244.1	3921439		Uncharacterized protein	68.24512	-1.97833	0.233737	-4.18559	2.84E-05	0.000269	Cluster 1
SAOUHSC_01784	YP_500289.1	3920426	rplT	50S ribosomal protein L20	349.1947	3.321237	0.555012	4.182317	2.89E-05	0.000272	Cluster 5
SAOUHSC_01134	YP_499676.1	3920733		Uncharacterized protein	7.806611	2.907526	0.456261	4.180774	2.91E-05	0.000273	Cluster 6
SAOUHSC_00352	YP_498941.1	3921825		Integrase-like protein	9.008195	2.813269	0.439028	4.130189	3.62E-05	0.000339	Cluster 6
SAOUHSC_01423	YP_499949.1	3920654		Uncharacterized protein	101.7993	1.954335	0.232751	4.100237	4.13E-05	0.000385	Cluster 6
SAOUHSC_T00040		3921029			92.64795	2.597076	0.393292	4.060792	4.89E-05	0.000454	Cluster 5
SAOUHSC_01783	YP_500288.1	3920425		Uncharacterized protein	75.06631	3.394806	0.590311	4.056857	4.97E-05	0.00046	Cluster 5
SAOUHSC_02423	YP_500894.1	3919633		Probable uridylyltransferase SAOUHSC_02423 (EC 2.7.7.-)	699.7148	-1.68039	0.168543	-4.03686	5.42E-05	0.000499	Cluster 1
SAOUHSC_02425	YP_500896.1	3919635		UPF0457 protein SAOUHSC_02425	203.798	2.556863	0.387049	4.022397	5.76E-05	0.000529	Cluster 4
SAOUHSC_02537	YP_501001.1	3921127	mobA	Probable molybdenum cofactor guanylyltransferase (MoCo guanylyltransferase) (EC 2.7.7.77) (GTP:molybdopterin guanylyltransferase) (Mo-MPT guanylyltransferase) (Molybdopterin guanylyltransferase) (Molybdopterin-guanine dinucleotide synthase) (MGD synthase)	238.468	-1.65434	0.162716	-4.02137	5.79E-05	0.000529	Cluster 1
SAOUHSC_00609	YP_499172.1	3918928		Uncharacterized protein	4.964856	-4.72284	0.926405	-4.01859	5.85E-05	0.000534	Cluster 1
SAOUHSC_01405	YP_499932.1	3920695		Uncharacterized protein	61.11614	2.210022	0.302942	3.994235	6.49E-05	0.000589	Cluster 5
SAOUHSC_02544	YP_501007.1	3921133		Molybdenum cofactor biosynthesis protein B	92.24767	-1.76562	0.192281	-3.98177	6.84E-05	0.000619	Cluster 1
SAOUHSC_00146	YP_498745.1	3919854		Uncharacterized protein	89.34202	-2.38024	0.347776	-3.96876	7.22E-05	0.000652	Cluster 1
SAOUHSC_01079	YP_499626.1	3919242		neurofilament protein	39.73008	2.852966	0.467333	3.964976	7.34E-05	0.00066	Cluster 5
SAOUHSC_00541	YP_499113.1	3920821	tadA	tRNA-specific adenosine deaminase (EC 3.5.4.33)	36.96414	2.206929	0.30532	3.953002	7.72E-05	0.000686	Cluster 6
SAOUHSC_00886	YP_499439.1	3919233	mnhD1	Na(+)/H(+) antiporter subunit D1 (Mnh complex subunit D1)	926.1551	-1.87435	0.221165	-3.95338	7.71E-05	0.000686	Cluster 1
SAOUHSC_01008	YP_499558.1	3920269	purE	N5-carboxyaminoimidazole ribonucleotide mutase (N5-CAIR mutase) (EC 5.4.99.18) (5-(carboxyamino)imidazole ribonucleotide mutase)	41.2167	2.605209	0.405909	3.954605	7.67E-05	0.000686	Cluster 5

SAOUHSC_02880	YP_501334.1	3921550	crtQ	4,4'-diaponeurosporenoate glycosyltransferase (EC 2.4.1.-)	299.1857	-2.20515	0.306014	-3.93824	8.21E-05	0.000727	Cluster 2
SAOUHSC_02851	YP_501308.1	3921524	cidA	Holin-like protein CidA	39.11957	-3.35961	0.601594	-3.92226	8.77E-05	0.000775	Cluster 1
SAOUHSC_T00023		3921149			40.00213	3.075785	0.529803	3.918033	8.93E-05	0.000785	Cluster 5
SAOUHSC_01129	YP_499672.1	3920729	arcC1	Carbamate kinase 1 (EC 2.7.2.2)	63.03168	-2.81047	0.463198	-3.90864	9.28E-05	0.000814	Cluster 1
SAOUHSC_02656	YP_501118.1	3921218		Uncharacterized protein	94.65978	-1.94222	0.242107	-3.89178	9.95E-05	0.00087	Cluster 1
SAOUHSC_00201	YP_498798.1	3920357		conserved hypothetical protein	55.83211	-3.27522	0.588976	-3.86302	0.000112	0.000975	Cluster 1
SAOUHSC_01415	YP_499942.1	3920647		Uncharacterized protein	568.5239	-1.81849	0.212316	-3.85507	0.000116	0.001004	Cluster 3
SAOUHSC_00208	YP_498804.1	3920363		Uncharacterized protein	14.25263	-2.67545	0.436018	-3.84261	0.000122	0.001053	Cluster 1
SAOUHSC_T00031		3921157			28.49517	2.661177	0.433374	3.833126	0.000127	0.001091	Cluster 5
SAOUHSC_00022	YP_498628.1	3919193		Uncharacterized protein	536.0863	-1.80552	0.210773	-3.82174	0.000133	0.001138	Cluster 1
SAOUHSC_03048	YP_501494.1	3921312		Uncharacterized protein	17.39251	-3.3398	0.612972	-3.81714	0.000135	0.001156	Cluster 1
SAOUHSC_01003	YP_499555.1	3920403		Uncharacterized protein	8.627678	3.757875	0.72356	3.811535	0.000138	0.001178	Cluster 5
SAOUHSC_01782	YP_500287.1	3919700		Uncharacterized protein	71.67742	2.771577	0.466406	3.798358	0.000146	0.001239	Cluster 6
SAOUHSC_01089	YP_499634.1	3919250	isdG	Heme oxygenase (staphylobilin-producing) 1 (EC 1.14.99.48) (Heme-degrading monooxygenase 1) (Iron-regulated surface determinant 1) (Iron-responsive surface determinant 1)	9.956762	3.178736	0.575168	3.787997	0.000152	0.001287	Cluster 6
SAOUHSC_T00058		3921047			18.51501	2.897774	0.501518	3.784058	0.000154	0.001303	Cluster 5
SAOUHSC_02347	YP_500824.1	3919391	atpF	ATP synthase subunit b (ATP synthase F(0) sector subunit b) (ATPase subunit I) (F-type ATPase subunit b) (F-ATPase subunit b)	937.9554	-1.58695	0.155518	-3.7742	0.000161	0.001351	Cluster 1
SAOUHSC_00373	YP_498962.1	3919792		Xanthine permease, putative	402.4248	2.266333	0.335713	3.772066	0.000162	0.001354	Cluster 6
SAOUHSC_01785	YP_500290.1	3920427	rpmI	50S ribosomal protein L35	429.2114	3.144435	0.56841	3.772691	0.000161	0.001354	Cluster 5
SAOUHSC_00023	YP_498629.1	3919194		Uncharacterized protein	300.6003	-1.92174	0.245931	-3.74796	0.000178	0.001486	Cluster 1
SAOUHSC_02329	YP_500808.1	3920954	thiM	Hydroxyethylthiazole kinase (EC 2.7.1.50) (4-methyl-5-beta-hydroxyethylthiazole kinase) (TH kinase) (Thz kinase)	36.33771	-2.81039	0.488106	-3.70901	0.000208	0.001729	Cluster 1
SAOUHSC_00144	YP_498743.1	3919852		Uncharacterized protein	648.9618	-1.73682	0.198968	-3.7032	0.000213	0.001763	Cluster 1
SAOUHSC_02646	YP_501108.1	3921208		Uncharacterized protein	52.53956	-2.11986	0.302616	-3.70058	0.000215	0.001775	Cluster 1
SAOUHSC_01904	YP_500405.1	3920851	crcB2	Putative fluoride ion transporter CrcB 2	12.1653	2.262352	0.341578	3.695647	0.000219	0.001804	Cluster 6
SAOUHSC_01014	YP_499564.1	3920275	purF	Amidophosphoribosyltransferase (ATase) (EC 2.4.2.14) (Glutamine phosphoribosylpyrophosphate amidotransferase) (GPATase)	138.8499	1.908359	0.246237	3.688958	0.000225	0.001847	Cluster 6
SAOUHSC_01086	YP_499631.1	3919247		iron compound ABC transporter permease protein putative	22.17357	3.129892	0.578967	3.678781	0.000234	0.001916	Cluster 6
SAOUHSC_01811	YP_500316.1	3919281	dnaE	DNA polymerase III subunit alpha (EC 2.7.7.7)	711.8171	-1.91331	0.248474	-3.6757	0.000237	0.001933	Cluster 1
SAOUHSC_00412	YP_498998.1	3920570		NADH dehydrogenase subunit 5, putative (EC 1.6.5.3)	326.4485	1.879441	0.239864	3.666413	0.000246	0.001998	Cluster 6
SAOUHSC_02402	YP_500878.1	3919617		PTS system, mannitol-specific IIa component, putative (EC 2.7.1.69)	157.6249	-1.67526	0.184472	-3.66052	0.000252	0.002038	Cluster 1
SAOUHSC_02956	YP_501407.1	3921659	nsaR	DNA-binding response regulator, putative	99.6463	-1.82735	0.226803	-3.64787	0.000264	0.002134	Cluster 1
SAOUHSC_01606	YP_500121.1	3920022		Peptidase T, putative (EC 3.4.11.14)	214.9255	-2.06288	0.291727	-3.64339	0.000269	0.002164	Cluster 1
SAOUHSC_00999	YP_499551.1	3920399	qoxD	Probable quinol oxidase subunit 4 (EC 1.10.3.-) (Quinol oxidase polypeptide IV)	845.8333	-2.23801	0.342609	-3.61347	0.000302	0.002423	Cluster 1
SAOUHSC_03035	YP_501483.1	3921301		Uncharacterized protein	249.0009	-2.12006	0.31095	-3.60205	0.000316	0.002524	Cluster 1
SAOUHSC_02983	YP_501432.1	3921465	gtfB	UDP-N-acetylglucosamine--peptide N-acetylglucosaminyltransferase stabilizing protein GtfB (Glycosyltransferase stabilizing protein GtfB)	402.7251	-1.74591	0.207868	-3.58836	0.000333	0.002652	Cluster 1
SAOUHSC_02549	YP_501011.1	3921137		Molybdenum ABC transporter, periplasmic molybdate-binding protein	213.7503	-2.332	0.371862	-3.58196	0.000341	0.002709	Cluster 1
SAOUHSC_01617	YP_500131.1	3920032	argR	Arginine repressor	119.6278	-1.68298	0.191086	-3.57423	0.000351	0.002782	Cluster 1
SAOUHSC_01099	YP_499643.1	3920741	mutS2	Endonuclease MutS2 (EC 3.1.-.-)	347.5195	-1.60363	0.169701	-3.55703	0.000375	0.002961	Cluster 1
SAOUHSC_02780	YP_501240.1	3921435		Uncharacterized protein	72.88298	-1.75536	0.212661	-3.55196	0.000382	0.003009	Cluster 1
SAOUHSC_01087	YP_499632.1	3919248		Iron compound ABC transporter, permease protein	12.63885	3.042512	0.575622	3.548355	0.000388	0.003041	Cluster 6
SAOUHSC_T00061		3921069			29.14237	3.249723	0.638841	3.521566	0.000429	0.003355	Cluster 5
SAOUHSC_01679	YP_500189.1	3920090		Uncharacterized protein	658.2267	1.994392	0.285913	3.47796	0.000505	0.003939	Cluster 5
SAOUHSC_02171	YP_500658.1	3921865		Staphylokinase, putative	88.5302	-2.62413	0.467633	-3.47308	0.000515	0.003999	Cluster 1
SAOUHSC_00353	YP_498942.1	3921826		Uncharacterized protein	5.450071	3.074865	0.598462	3.466994	0.000526	0.004075	Cluster 6
SAOUHSC_01395	YP_499922.1	3920685	asd	Aspartate-semialdehyde dehydrogenase (ASA dehydrogenase) (ASADH) (EC 1.2.1.11) (Aspartate-beta-semialdehyde dehydrogenase)	1297.924	2.507969	0.435025	3.466397	0.000527	0.004075	Cluster 4
SAOUHSC_03018	YP_501467.1	3921285		Uncharacterized protein	66.62694	3.083619	0.601271	3.46536	0.00053	0.004078	Cluster 6
SAOUHSC_T0001		3921517			23.02221	3.124374	0.614681	3.456059	0.000548	0.004209	Cluster 6
SAOUHSC_00775	YP_499332.1	3919066		Uncharacterized protein	35.69203	2.172301	0.339457	3.453462	0.000553	0.004237	Cluster 6
SAOUHSC_00297	YP_498887.1	3918978		Uncharacterized protein	120.6503	1.977222	0.283249	3.450041	0.000561	0.004278	Cluster 5
SAOUHSC_02346	YP_500823.1	3919390	atpH	ATP synthase subunit delta (ATP synthase F(1) sector subunit delta) (F-type ATPase subunit delta) (F-ATPase subunit delta)	829.4274	-1.61152	0.177847	-3.43845	0.000585	0.004452	Cluster 1
SAOUHSC_01001	YP_499553.1	3920401	qoxB	Probable quinol oxidase subunit 1 (EC 1.10.3.-) (Quinol oxidase polypeptide I)	4950.874	-2.2561	0.365608	-3.43565	0.000591	0.004485	Cluster 1

SAOUHSC_02350	YP_500826.1	3919393	atpB	ATP synthase subunit a (ATP synthase F0 sector subunit a) (F-ATPase subunit 6)	1232.134	-1.58947	0.171685	-3.43341	0.000596	0.004509	Cluster 1
SAOUHSC_01065	YP_499611.1	3921727	ctaA	Heme A synthase (HAS) (EC 1.3.-.-) (Cytochrome aa3-controlling protein)	174.2664	-1.52281	0.152394	-3.43063	0.000602	0.004542	Cluster 1
SAOUHSC_02254	YP_500736.1	3919674	groEL	60 kDa chaperone family; promotes refolding of misfolded polypeptides especially under stressful conditions; forms two stacked rings of heptamers to form a barrel-shaped 14mer; ends can be capped by GroES; misfolded proteins enter the barrel where they are refolded when GroES binds; many bacteria have multiple copies of the groEL gene which are active under different environmental conditions; the B.japonicum protein in this cluster is expressed constitutively; in Rhodobacter, Corynebacterium and Rhizobium this protein is essential for growth	6220.094	2.666605	0.486465	3.425952	0.000613	0.004607	Cluster 4
SAOUHSC_01012	YP_499562.1	3920273	purQ	Phosphoribosylformylglycinamidine synthase subunit PurQ (FGAM synthase) (EC 6.3.5.3) (Formylglycinamide ribonucleotide amidotransferase subunit I) (FGAR amidotransferase I) (FGAR-AT I) (Glutaminase PurQ) (EC 3.5.1.2) (Phosphoribosylformylglycinamidine synthase subunit I)	46.49895	1.726092	0.212235	3.421171	0.000624	0.004675	Cluster 5
SAOUHSC_01796	YP_500300.1	3920437		Formamidopyrimidine-DNA glycosylase (EC 3.2.2.-) (EC 3.2.2.23) (EC 4.2.99.18)	258.1607	-1.74528	0.218524	-3.41052	0.000648	0.004847	Cluster 1
SAOUHSC_00957	YP_499510.1	3920668		Uncharacterized protein	183.7123	2.881107	0.55588	3.384016	0.000714	0.005324	Cluster 5
SAOUHSC_02538	YP_501002.1	3921128		Molybdopterin converting factor, subunit 1	106.5202	-1.87285	0.258438	-3.37742	0.000732	0.005437	Cluster 1
SAOUHSC_00967	YP_499520.1	3920112		Uncharacterized protein	3.650673	3.273204	0.675503	3.365202	0.000765	0.005667	Cluster 6
SAOUHSC_01233	YP_499766.1	3920258		Uncharacterized protein	275.8199	2.71413	0.513055	3.341023	0.000835	0.006151	Cluster 5
SAOUHSC_03019	YP_501468.1	3921286		ABC transporter, ATP-binding protein, putative	165.4829	2.787867	0.535146	3.340897	0.000835	0.006151	Cluster 6
SAOUHSC_01894	YP_500395.1	3920841	arsC	Arsenate reductase (EC 1.20.4.4)	24.74373	2.182976	0.354981	3.332506	0.000861	0.006322	Cluster 5
SAOUHSC_02645	YP_501107.1	3921207		Uncharacterized HTH-type transcriptional regulator SAOUHSC_02645	52.71219	-2.40726	0.424204	-3.31742	0.000909	0.006654	Cluster 1
SAOUHSC_01223	YP_499759.1	3920251	gid	TrmFO; Gid; glucose-inhibited division protein; similar to GidA; the gene from Bacillus subtilis encodes a tRNA-methyltransferase that utilizes folate as the carbon donor and bound flavin as reductant; modifies tRNA at position 54 (uridine) of the T-psi loop to form a C5-methyluridine	491.5372	2.080805	0.325932	3.316046	0.000913	0.006667	Cluster 6
SAOUHSC_02685	YP_501147.1	3919704		Uncharacterized protein	8.381982	-3.12755	0.642262	-3.31259	0.000924	0.006731	Cluster 1
SAOUHSC_00883	YP_499436.1	3919230	mnhG1	Na(+)/H(+) antiporter subunit G1 (Mnh complex subunit G1)	247.0121	-1.79243	0.239941	-3.30259	0.000958	0.006955	Cluster 1
SAOUHSC_02379	YP_500856.1	3919422	deoC	Deoxyribose-phosphate aldolase (DERA) (EC 4.1.2.4) (2-deoxy-D-ribose 5-phosphate aldolase) (Phosphodeoxyriboaldolase) (Deoxyriboaldolase)	467.4846	-1.75312	0.230562	-3.26645	0.001089	0.007885	Cluster 1
SAOUHSC_00910	YP_499463.1	3920797		Uncharacterized protein	225.743	1.750966	0.230817	3.253516	0.00114	0.008229	Cluster 5
SAOUHSC_00776	YP_499333.1	3919067		Excinuclease ABC, B subunit	199.0672	-1.85455	0.26312	-3.24777	0.001163	0.008373	Cluster 1
SAOUHSC_00132	YP_498732.1	3919841	aldA	Putative aldehyde dehydrogenase AldA (EC 1.2.1.3)	842.6101	1.953716	0.29411	3.242717	0.001184	0.008499	Cluster 4
SAOUHSC_01000	YP_499552.1	3920400	qoxC	Probable quinol oxidase subunit 3 (EC 1.10.3.-) (Quinol oxidase polypeptide III)	2134.193	-2.27441	0.393681	-3.23716	0.001207	0.008642	Cluster 1
SAOUHSC_02972	YP_501423.1	3921673	isaB	Immunodominant staphylococcal antigen B	245.7556	-2.13486	0.352181	-3.22238	0.001271	0.009075	Cluster 1
SAOUHSC_00355	YP_498944.1	3921828		Uncharacterized protein	6.582603	2.909186	0.595155	3.207878	0.001337	0.009518	Cluster 5
SAOUHSC_01411	YP_499938.1	3920643		Putative branched-chain amino acid carrier protein SAOUHSC_01411	102.7792	2.173482	0.36599	3.206326	0.001344	0.009543	Cluster 5
SAOUHSC_02753	YP_501212.1	3921625		Membrane protein, putative	238.4299	-1.6712	0.209562	-3.20289	0.001361	0.00963	Cluster 1
SAOUHSC_01326	YP_499856.1	3920191		Uncharacterized protein	534.3122	2.52231	0.477181	3.190218	0.001422	0.010035	Cluster 6
SAOUHSC_00779	YP_499336.1	3919069		UvrABC system protein B	225.7169	-1.92747	0.292433	-3.17155	0.001516	0.010673	Cluster 1
SAOUHSC_00898	YP_499451.1	3921744	argH	Argininosuccinate lyase (ASAL) (EC 4.3.2.1) (Arginosuccinase)	560.8832	1.601213	0.189833	3.167056	0.00154	0.010809	Cluster 4
SAOUHSC_01981	YP_500478.1	3920457		Sensor histidine kinase, putative	82.57372	1.635103	0.201603	3.150271	0.001631	0.011418	Cluster 6
SAOUHSC_01010	YP_499560.1	3920271	purC	Phosphoribosylaminoimidazole-succinocarboxamide synthase (EC 6.3.2.6) (SAICAR synthetase)	33.98492	1.869536	0.276308	3.14698	0.00165	0.01149	Cluster 6
SAOUHSC_02351	YP_500827.1	3919394		Uncharacterized protein	753.3143	-1.56966	0.181027	-3.14684	0.00165	0.01149	Cluster 1
SAOUHSC_01673	YP_500184.1	3920085		Uncharacterized protein	200.8996	-1.86843	0.276176	-3.14448	0.001664	0.011551	Cluster 1
SAOUHSC_01992	YP_500489.1	3921875		Phosphotransferase system, EIIC domain protein	102.5711	2.040953	0.331219	3.142792	0.001673	0.011586	Cluster 6
SAOUHSC_02403	YP_500879.1	3919618	mtlD	Mannitol-1-phosphate 5-dehydrogenase (EC 1.1.1.17)	266.1138	-1.49784	0.158633	-3.13833	0.001699	0.011732	Cluster 1
SAOUHSC_00806	YP_499362.1	3919369		Uncharacterized protein	34.90498	-1.92023	0.294602	-3.12364	0.001786	0.0123	Cluster 1
SAOUHSC_00266	YP_498859.1	3919207		Uncharacterized protein	18.89888	-2.14519	0.366791	-3.12219	0.001795	0.012327	Cluster 1
SAOUHSC_00133	YP_498733.1	3919842		Uncharacterized protein	102.5129	-1.75545	0.242362	-3.11704	0.001827	0.012511	Cluster 1
SAOUHSC_02447	YP_500914.1	3919010		Uncharacterized protein	579.5431	-1.76037	0.244339	-3.11195	0.001859	0.012694	Cluster 3
SAOUHSC_01193	YP_499732.1	3919326		Uncharacterized protein SAOUHSC_01193	488.1639	1.789466	0.254109	3.106793	0.001891	0.012883	Cluster 5
SAOUHSC_02444	YP_500912.1	3919008		Osmoprotectant transporter, BCCT family, opuD-like protein, putative	539.054	1.907301	0.293898	3.087131	0.002021	0.01373	Cluster 6

SAOUHSC_02955	YP_501406.1	3921658	nsaS	Sensor histidine kinase, putative	88.27115	-1.81506	0.264504	-3.08146	0.00206	0.013956	Cluster 1
SAOUHSC_00160	YP_498758.1	3919867		Uncharacterized protein	113.0992	-1.91718	0.298375	-3.07392	0.002113	0.014276	Cluster 1
SAOUHSC_00808	YP_499364.1	3919371		Uncharacterized protein	53.01123	-1.79603	0.259342	-3.06941	0.002145	0.014455	Cluster 1
SAOUHSC_00683	YP_499242.1	3919446		Uncharacterized protein	32.08712	1.94408	0.307735	3.067829	0.002156	0.014493	Cluster 6
SAOUHSC_00533	YP_499105.1	3920386	hchA	Protein/nucleic acid deglycase HchA (EC 3.1.2.-) (EC 3.5.1.-) (EC 3.5.1.124) (Maillard deglycase)	1116.474	2.167147	0.380834	3.064711	0.002179	0.014606	Cluster 5
SAOUHSC_00647	YP_499207.1	3919939		Uncharacterized protein	923.817	-1.71674	0.234518	-3.0562	0.002242	0.014987	Cluster 3
SAOUHSC_T00013		3921332			35.01336	2.385438	0.455202	3.043566	0.002338	0.01559	Cluster 5
SAOUHSC_00807	YP_499363.1	3919370		Uncharacterized protein	31.25636	-1.97555	0.321404	-3.03527	0.002403	0.015963	Cluster 1
SAOUHSC_01015	YP_499565.1	3920276	purM	Phosphoribosylformylglycinamidine cyclo-ligase (EC 6.3.3.1) (AIR synthase) (AIRS) (Phosphoribosyl-aminoimidazole synthetase)	121.1789	1.910278	0.29994	3.034865	0.002406	0.015963	Cluster 6
SAOUHSC_00559	YP_499131.1	3920604		Protein VraC	62.45921	3.081252	0.68694	3.029744	0.002448	0.016194	Cluster 5
SAOUHSC_02923	YP_501376.1	3921374		Uncharacterized protein	1402.674	2.496547	0.494757	3.024811	0.002488	0.016417	Cluster 6
SAOUHSC_00198	YP_498795.1	3920354		Uncharacterized protein	118.2726	-1.87714	0.290218	-3.02234	0.002508	0.016509	Cluster 1
SAOUHSC_02727	YP_501190.1	3921603		Uncharacterized protein	141.8918	-1.76243	0.252636	-3.01788	0.002545	0.01671	Cluster 1
SAOUHSC_00914	YP_499467.1	3920801		2-isopropylmalate synthase, putative (EC 2.3.3.13)	12.01014	3.525065	0.837663	3.014417	0.002575	0.016859	Cluster 5
SAOUHSC_02588	YP_501050.2	3921584		Uncharacterized protein	6.201684	2.613745	0.536619	3.007245	0.002636	0.017217	Cluster 6
SAOUHSC_00572	YP_499140.1	3920613		Uncharacterized protein	73.74128	1.913551	0.304231	3.002818	0.002675	0.017425	Cluster 6
SAOUHSC_02760	YP_501219.1	3921632		Glutamate synthase alpha subunit, putative (EC 1.4.1.13)	154.3401	-1.55018	0.184136	-2.98788	0.002809	0.018253	Cluster 1
SAOUHSC_02331	YP_500810.1	3920956	tenA	Aminopyrimidine aminohydrolase (EC 3.5.99.2) (Thiaminase II)	13.4151	-2.30632	0.437433	-2.98634	0.002823	0.018298	Cluster 1
SAOUHSC_00915	YP_499468.1	3920802		Uncharacterized protein	8.02473	2.881141	0.630099	2.985467	0.002831	0.018303	Cluster 5
SAOUHSC_00916	YP_499469.1	3920803		Uncharacterized protein	12.13568	2.209733	0.406894	2.973091	0.002948	0.019009	Cluster 6
SAOUHSC_01574	YP_500089.1	3920168		Helix-turn-helix domain protein	20.94457	3.009569	0.676156	2.972048	0.002958	0.019026	Cluster 4
SAOUHSC_00010	YP_498618.1	3919183		Uncharacterized protein	192.6292	2.437167	0.484841	2.964202	0.003035	0.019468	Cluster 6
SAOUHSC_R0008		3921508			69620.65	2.755252	0.594892	2.95054	0.003172	0.020299	Cluster 4
SAOUHSC_00798	YP_499354.1	3919361	gpmI	2,3-bisphosphoglycerate-independent phosphoglycerate mutase (BPG-independent PGAM) (Phosphoglyceromutase) (iPGM) (EC 5.4.2.12)	1917.87	-1.7294	0.247862	-2.94276	0.003253	0.020763	Cluster 1
SAOUHSC_01316	YP_499846.1	3920142		Uncharacterized protein	11.63611	2.141954	0.389041	2.935305	0.003332	0.021216	Cluster 6
SAOUHSC_01208	YP_499745.1	3919474	rpsP	30S ribosomal protein S16	161.3786	2.317022	0.450466	2.923687	0.003459	0.021968	Cluster 5
SAOUHSC_02349	YP_500825.1	3919392	atpE	ATP synthase subunit c (ATP synthase F(0) sector subunit c) (F-type ATPase subunit c) (F-ATPase subunit c) (Lipid-binding protein)	632.8089	-1.62444	0.213883	-2.91952	0.003506	0.022209	Cluster 1
SAOUHSC_01786	YP_500291.1	3920428	infC	Translation initiation factor IF-3	575.2045	2.729848	0.592842	2.91789	0.003524	0.022269	Cluster 5
SAOUHSC_01205	YP_499742.1	3919471	ftsY	Signal recognition particle receptor FtsY (SRP receptor)	199.5592	-1.7319	0.251875	-2.9058	0.003663	0.023091	Cluster 1
SAOUHSC_02124	YP_500615.1	3921195	pcrB	Heptaprenylglyceryl phosphate synthase (HepGP synthase) (EC 2.5.1.n9) (Glycerol-1-phosphate heptaprenyltransferase)	29.24298	1.705495	0.243086	2.902244	0.003705	0.023296	Cluster 6
SAOUHSC_02093	YP_500585.1	3921166		UPF0435 protein SAOUHSC_02093	28.05067	2.129303	0.389528	2.899155	0.003742	0.023469	Cluster 6
SAOUHSC_01336	YP_499865.1	3920200		UPF0291 protein SAOUHSC_01336	81.18322	1.858224	0.296789	2.891695	0.003832	0.023953	Cluster 4
SAOUHSC_01372	YP_499899.1	3920781	trpA	Tryptophan synthase alpha chain (EC 4.2.1.20)	25.71409	1.785546	0.271703	2.891196	0.003838	0.023953	Cluster 6
SAOUHSC_03021	YP_501470.1	3921288		Uncharacterized protein	92.6622	2.729718	0.600437	2.880766	0.003967	0.024699	Cluster 6
SAOUHSC_00009	YP_498617.1	3919182	serS	Serine--tRNA ligase (EC 6.1.1.11) (Seryl-tRNA synthetase) (SerRS) (Seryl-tRNA(Ser/Sec) synthetase)	594.9093	-1.93725	0.326232	-2.87296	0.004067	0.025256	Cluster 2
SAOUHSC_01632	YP_500145.1	3919969	gcvPB	Probable glycine dehydrogenase (decarboxylating) subunit 2 (EC 1.4.4.2) (Glycine cleavage system P-protein subunit 2) (Glycine decarboxylase subunit 2) (Glycine dehydrogenase (aminomethyl-transferring) subunit 2)	355.3151	1.575909	0.201283	2.861193	0.00422	0.026149	Cluster 6
SAOUHSC_00833	YP_499387.1	3918945		Uncharacterized protein	201.4714	1.96434	0.337455	2.857687	0.004267	0.026375	Cluster 4
SAOUHSC_02281	YP_500763.1	3919156	ilvD	Dihydroxy-acid dehydratase (DAD) (EC 4.2.1.9)	514.4671	2.104743	0.386828	2.855902	0.004291	0.026459	Cluster 5
SAOUHSC_00582	YP_499149.1	3920506		Uncharacterized protein	22.3863	1.958155	0.335798	2.85337	0.004326	0.026598	Cluster 5
SAOUHSC_02345	YP_500822.1	3920968	atpA	ATP synthase subunit alpha (EC 7.1.2.2) (ATP synthase F1 sector subunit alpha) (F-ATPase subunit alpha)	1960.728	-1.57048	0.199978	-2.8527	0.004335	0.026598	Cluster 1
SAOUHSC_00686	YP_499245.1	3920990		Uncharacterized protein	116.6681	1.594262	0.208446	2.850913	0.004359	0.026683	Cluster 6
SAOUHSC_01891	YP_500393.1	3920839		Arsenate operon regulator	23.3786	2.778805	0.625495	2.843836	0.004457	0.027217	Cluster 6
SAOUHSC_02010	YP_500507.1	3920464		Uncharacterized protein	57.27234	1.672731	0.237919	2.827558	0.00469	0.028571	Cluster 6
SAOUHSC_T00015		3921334			62.23177	2.195856	0.426437	2.804296	0.005043	0.030624	Cluster 5
SAOUHSC_T00016		3921335			123.02	1.989367	0.352876	2.803726	0.005052	0.030624	Cluster 5
SAOUHSC_00603	YP_499166.1	3918922		Uncharacterized protein	132.9141	-1.46917	0.167543	-2.80029	0.005106	0.030731	Cluster 1
SAOUHSC_01653	YP_500165.1	3920105	sodA	Superoxide dismutase [Mn] 1 (EC 1.15.1.1)	1132.688	1.626875	0.223762	2.801521	0.005086	0.030731	Cluster 5
SAOUHSC_01655	YP_500166.1	3920106	fur	Ferric uptake regulation protein (Ferric uptake regulator)	40.63946	2.270729	0.453681	2.800934	0.005095	0.030731	Cluster 6
SAOUHSC_R0007		3921507			66576.62	2.678523	0.601072	2.792549	0.005229	0.03139	Cluster 4

SAOUHSC_01799	YP_500303.1	3920440		Histidine kinase-, DNA gyrase B-, and HSP90-like ATPase domain protein	241.254	-1.3901	0.139727	-2.7919	0.00524	0.03139	Cluster 1
SAOUHSC_02378	YP_500855.1	3919421		Uncharacterized protein	126.3061	-1.79004	0.284888	-2.77317	0.005551	0.033077	Cluster 1
SAOUHSC_02545	YP_501008.1	3921134		Uncharacterized protein	127.9596	-1.54664	0.197151	-2.7727	0.005559	0.033077	Cluster 1
SAOUHSC_02890	YP_501343.1	3921342		Uncharacterized protein	7.205414	2.648327	0.594501	2.772621	0.005561	0.033077	Cluster 6
SAOUHSC_01370	YP_499897.1	3920779	trpF	N-(5'-phosphoribosyl)anthranilate isomerase (PRAI) (EC 5.3.1.24)	14.13797	1.990376	0.357457	2.770617	0.005595	0.033203	Cluster 6
SAOUHSC_01647	YP_500159.1	3920099		Uncharacterized protein	127.9358	-1.46023	0.166637	-2.76189	0.005747	0.033866	Cluster 1
SAOUHSC_01676	YP_500186.1	3920087		UPF0365 protein SAOUHSC_01676	1874.009	-1.62704	0.226975	-2.7626	0.005734	0.033866	Cluster 3
SAOUHSC_03013	YP_501462.1	3921494	hisD	Histidinol dehydrogenase (HDH) (EC 1.1.1.23)	91.14511	2.136278	0.411324	2.762491	0.005736	0.033866	Cluster 4
SAOUHSC_03020	YP_501469.1	3921287		UPF0397 protein SAOUHSC_03020	89.57056	2.680687	0.608838	2.760482	0.005772	0.033933	Cluster 6
SAOUHSC_00174	YP_498771.1	3919488		M23/M37 peptidase domain protein	83.66858	1.931861	0.337784	2.758752	0.005802	0.034035	Cluster 4
SAOUHSC_R0006		3921506			67433.66	2.665009	0.604203	2.75571	0.005856	0.034194	Cluster 4
SAOUHSC_03017	YP_501466.1	3921284		Uncharacterized protein	38.74323	2.82025	0.660404	2.756266	0.005847	0.034194	Cluster 6
SAOUHSC_00217	YP_498812.1	3920293		Sorbitol dehydrogenase, putative (EC 1.1.1.14)	73.69942	1.813161	0.29522	2.754421	0.00588	0.03425	Cluster 6
SAOUHSC_01387	YP_499914.1	3920678		Phosphate transport system permease protein	11.89852	2.771672	0.643655	2.752519	0.005914	0.034371	Cluster 5
SAOUHSC_01443	YP_499968.1	3920224		Uncharacterized protein	3.598695	2.836538	0.669607	2.742711	0.006093	0.035333	Cluster 6
SAOUHSC_01054	YP_499602.1	3921718		UPF0637 protein SAOUHSC_01054	88.60246	2.133882	0.413631	2.74129	0.00612	0.035406	Cluster 5
SAOUHSC_02584	YP_501046.1	3921580		Uncharacterized protein	115.7779	-1.44219	0.161527	-2.73754	0.00619	0.03573	Cluster 1
SAOUHSC_T00017		3921336			25.9067	2.815778	0.663588	2.736302	0.006213	0.035784	Cluster 5
SAOUHSC_02995	YP_501444.1	3921477		Uncharacterized protein	71.5096	1.753331	0.275972	2.729736	0.006339	0.036421	Cluster 6
SAOUHSC_02932	YP_501385.1	3921639	betA	Oxygen-dependent choline dehydrogenase (CDH) (CHD) (EC 1.1.99.1) (Betaine aldehyde dehydrogenase) (BADH) (EC 1.2.1.8)	35.21533	2.938868	0.713565	2.717157	0.006585	0.037749	Cluster 6
SAOUHSC_01795	YP_500299.1	3920436	coaE	Dephospho-CoA kinase (EC 2.7.1.24) (Dephosphocoenzyme A kinase)	201.9352	-1.62819	0.231447	-2.71418	0.006644	0.038004	Cluster 1
SAOUHSC_01646	YP_500158.1	3920098		Glucokinase, putative	426.3916	-1.40677	0.150262	-2.70705	0.006788	0.038743	Cluster 1
SAOUHSC_02381	YP_500858.1	3919424		Uncharacterized protein	2375.388	2.538359	0.568816	2.704492	0.006841	0.038955	Cluster 4
SAOUHSC_03016	YP_501465.1	3921283		Uncharacterized protein	66.6626	2.384658	0.514048	2.693637	0.007068	0.040156	Cluster 6
SAOUHSC_02922	YP_501374.1	3921373	ldh2	L-lactate dehydrogenase 2 (L-LDH 2) (EC 1.1.1.27)	389.7726	-2.22131	0.454035	-2.68991	0.007147	0.040517	Cluster 2
SAOUHSC_00812	YP_499368.1	3919375	clfA	Clumping factor A (Fibrinogen receptor A) (Fibrinogen-binding protein A)	2093.811	-1.93469	0.3489	-2.67896	0.007385	0.041772	Cluster 1
SAOUHSC_01371	YP_499898.1	3920780	trpB	Tryptophan synthase beta chain (EC 4.2.1.20)	30.25294	1.796774	0.298768	2.666863	0.007656	0.043209	Cluster 6
SAOUHSC_01276	YP_499809.1	3919929	glpK	Glycerol kinase (EC 2.7.1.30) (ATP:glycerol 3-phosphotransferase) (Glycerokinase) (GK)	145.5873	1.806639	0.302652	2.665237	0.007693	0.043322	Cluster 6
SAOUHSC_02910	YP_501363.1	3921362		Uncharacterized protein	75.43386	-1.72498	0.272798	-2.65758	0.00787	0.04422	Cluster 1
SAOUHSC_02521	YP_500986.1	3921112		Uncharacterized protein	42.80229	1.591139	0.222501	2.656791	0.007889	0.044226	Cluster 6
SAOUHSC_02937	YP_501390.2	3921644		Choline transporter, putative	76.98837	2.99705	0.752142	2.655151	0.007927	0.044344	Cluster 5
SAOUHSC_02666	YP_501128.1	3921228		Uncharacterized protein	27.74228	2.134048	0.42987	2.638116	0.008337	0.046532	Cluster 6
SAOUHSC_01320	YP_499850.1	3920146		Homoserine dehydrogenase (EC 1.1.1.3)	1185.118	1.373212	0.141884	2.630396	0.008529	0.047497	Cluster 6
SAOUHSC_00356	YP_498945.1	3921829		Uncharacterized protein	1085.723	2.108866	0.422277	2.62592	0.008642	0.048021	Cluster 6
SAOUHSC_02690	YP_501152.1	3919709		Uncharacterized protein	54.42703	1.785093	0.29934	2.622748	0.008722	0.048364	Cluster 6
SAOUHSC_01002	YP_499554.1	3920402	qoxA	Probable quinol oxidase subunit 2 (EC 1.10.3.-) (Quinol oxidase polypeptide II)	2398.562	-1.92863	0.355191	-2.61446	0.008937	0.049446	Cluster 1

S. aureus – 16 hpi vs. 1 hpi

Locus_id	Protein_id	Gene_id	Name	Description	baseMean	log2FoldChange	lfcSE	stat	pvalue	padj	Cluster
SAOUHSC_00036	YP_498642.1	3919304		Uncharacterized protein	645.3304841	4.209884625	0.240098733	13.36901945	9.17423E-41	2.32475E-37	Cluster 4
SAOUHSC_00898	YP_499451.1	3921744	argH	Argininosuccinate lyase (ASAL) (EC 4.3.2.1) (Arginosuccinase)	560.8832092	3.188092393	0.198580721	11.01865468	3.10667E-28	3.93616E-25	Cluster 4
SAOUHSC_00173	YP_498770.1	3919487	azoR	FMN-dependent NADH-azoreductase (EC 1.7.1.17) (Azo-dye reductase) (FMN-dependent NADH-azo compound oxidoreductase)	258.5358615	4.548861011	0.345973924	10.25759677	1.09395E-24	9.24026E-22	Cluster 4
SAOUHSC_00659	YP_499217.1	3919949		Uncharacterized protein	1617.640665	2.58667356	0.161406165	9.830315724	8.3357E-23	5.28067E-20	Cluster 4
SAOUHSC_00035	YP_498641.1	3919303		Uncharacterized protein	411.0784292	5.25361497	0.43811182	9.708971044	2.7611E-22	1.16611E-19	Cluster 4
SAOUHSC_00899	YP_499452.1	3921745	argG	Argininosuccinate synthase (EC 6.3.4.5) (Citrulline--aspartate ligase)	475.9186851	3.207543832	0.227019484	9.724028057	2.38169E-22	1.16611E-19	Cluster 4
SAOUHSC_00033	YP_498639.1	3919301		Probable membrane transporter protein	280.0107598	3.062243063	0.243725369	8.461339395	2.64324E-17	9.56853E-15	Cluster 4
SAOUHSC_01363	YP_499890.1	3920068		Uncharacterized protein	385.186297	3.774935769	0.3410145	8.137295533	4.04205E-16	1.28032E-13	Cluster 3
SAOUHSC_03022	YP_501471.1	3921289		UPF0312 protein SAOUHSC_03022	265.3923101	3.22754281	0.285207121	7.810263642	5.70685E-15	1.60679E-12	Cluster 4
SAOUHSC_00034	YP_498640.1	3919302		Uncharacterized protein	131.1252476	3.434234962	0.317728517	7.661367585	1.83963E-14	4.66163E-12	Cluster 4
SAOUHSC_00437	YP_499020.1	3919109		Uncharacterized protein	1198.814839	3.232721934	0.295076527	7.566586046	3.8316E-14	8.82662E-12	Cluster 4
SAOUHSC_02258	YP_500740.1	3919678		Uncharacterized protein	383.6338158	3.010517417	0.270220452	7.440285884	1.00468E-13	2.12154E-11	Cluster 4
SAOUHSC_00561	YP_499133.1	3920606		Uncharacterized protein	9001.938896	4.505946442	0.472611058	7.418248857	1.18679E-13	2.31333E-11	Cluster 4
SAOUHSC_00535	YP_499107.1	3920815		Uncharacterized epimerase/dehydratase SAOUHSC_00535	601.6012902	3.130280903	0.297788304	7.15367552	8.44846E-13	1.52917E-10	Cluster 3
SAOUHSC_01262	YP_499795.2	3919993	recA	Protein RecA (Recombinase A)	2059.139015	1.87351611	0.124073817	7.040293673	1.91835E-12	3.24073E-10	Cluster 4
SAOUHSC_02692	YP_501154.1	3919711		Antitoxin	134.3801745	2.783807421	0.276969913	6.44043752	1.1913E-10	1.88671E-08	Cluster 4
SAOUHSC_02691	YP_501153.1	3919710		Uncharacterized protein	179.1619839	2.866734665	0.296404685	6.297925639	3.01655E-10	4.49644E-08	Cluster 4
SAOUHSC_02658	YP_501120.1	3921220		Membrane protein, putative	444.844508	-3.337245841	0.377582898	-6.190020391	6.01564E-10	8.46869E-08	Cluster 2
SAOUHSC_01997	YP_500494.1	3921877	perR	Peroxide-responsive repressor PerR	1514.389332	2.110533128	0.181515323	6.118123319	9.46838E-10	1.26278E-07	Cluster 3
SAOUHSC_00037	YP_498643.1	3919305		Uncharacterized protein	459.301566	2.034175826	0.17118985	6.041104806	1.53063E-09	1.9393E-07	Cluster 4
SAOUHSC_01365	YP_499892.1	3920070		Uncharacterized protein	305.5337718	2.087020792	0.184666122	5.886411557	3.94671E-09	4.76236E-07	Cluster 3
SAOUHSC_01333	YP_499863.1	3920198	lexA	LexA repressor (EC 3.4.21.88)	1149.072462	2.105087705	0.196231508	5.631550793	1.78596E-08	2.05711E-06	Cluster 3
SAOUHSC_00880	YP_499433.1	3919227		Uncharacterized protein	359.4019252	1.874776792	0.157328823	5.560181376	2.69494E-08	2.96913E-06	Cluster 4
SAOUHSC_00319	YP_498909.1	3919585		Uncharacterized protein	247.2955235	2.48692426	0.271550159	5.475689146	4.35812E-08	4.60145E-06	Cluster 3
SAOUHSC_02757	YP_501216.1	3921629		Antitoxin	411.1625427	2.488592404	0.279430754	5.327231809	9.97209E-08	1.01077E-05	Cluster 4
SAOUHSC_01389	YP_499916.1	3920680	pstS	Phosphate-binding protein PstS (PBP)	42.29841808	3.705151356	0.51848825	5.217382177	1.8147E-07	1.76863E-05	Cluster 4
SAOUHSC_00703	YP_499262.1	3921007		Quinolone resistance norA protein, putative	201.3623861	-2.600771125	0.313169269	-5.11152045	3.19576E-07	2.99928E-05	Cluster 2
SAOUHSC_02661	YP_501123.1	3921223		PTS system sucrose-specific IIBC component, putative (EC 2.7.1.69)	209.4228215	1.972312996	0.193831524	5.016278975	5.26818E-07	4.7677E-05	Cluster 3
SAOUHSC_R0004		3921504			55386.13645	3.695922996	0.539536094	4.996742617	5.83068E-07	5.09481E-05	Cluster 4
SAOUHSC_00365	YP_498954.1	3919784	ahpC	Alkyl hydroperoxide reductase C (EC 1.11.1.15) (Peroxiredoxin) (Thioredoxin peroxidase)	1471.220977	2.487885057	0.300891201	4.944927117	7.61724E-07	6.39536E-05	Cluster 4
SAOUHSC_R0002		3921502			52568.75687	3.69511552	0.545601849	4.939711116	7.82384E-07	6.39536E-05	Cluster 4
SAOUHSC_R0003		3921503			49318.74763	3.688095435	0.546954472	4.914660309	8.89365E-07	6.87406E-05	Cluster 4
SAOUHSC_R0005		3921505			47135.17388	3.704730245	0.550482763	4.913378633	8.95202E-07	6.87406E-05	Cluster 4
SAOUHSC_R0001		3921501			49146.54944	3.696992376	0.549617985	4.907030793	9.24655E-07	6.8914E-05	Cluster 4
SAOUHSC_00364	YP_498953.1	3919783	ahpF	Alkyl hydroperoxide reductase subunit F (EC 1.8.1.-)	3968.768593	2.617324359	0.330147907	4.898787259	9.643E-07	6.98153E-05	Cluster 4
SAOUHSC_02756	YP_501215.1	3921628		Uncharacterized protein	437.5758361	2.358322707	0.278027375	4.885571807	1.03129E-06	7.25914E-05	Cluster 4
SAOUHSC_01005	YP_499556.1	3920404		Uncharacterized protein	383.7309431	2.61600221	0.333134594	4.850898816	1.22903E-06	8.41721E-05	Cluster 3
SAOUHSC_01121	YP_499665.1	3920722		alpha-hemolysin precursor	497.734849	2.605274199	0.352416735	4.555045322	5.23743E-06	0.000349254	Cluster 3
SAOUHSC_02573	YP_501034.1	3921570		Na+/H+ antiporter NhaC	199.3227813	-2.333487394	0.295822898	-4.507722031	6.55273E-06	0.00042576	Cluster 6
SAOUHSC_00318	YP_498908.1	3919584		Uncharacterized protein	133.2303003	2.331486442	0.306853114	4.339165483	1.43025E-05	0.000906062	Cluster 3
SAOUHSC_02969	YP_501419.1	3921670	arcA	Arginine deiminase (ADI) (EC 3.5.3.6) (Arginine dihydrolase) (AD)	32.40497061	3.255508491	0.520527575	4.33312009	1.47011E-05	0.000908599	Cluster 3
SAOUHSC_00320	YP_498910.1	3919586		NADPH-dependent FMN reductase, putative	129.8521784	2.356841039	0.315153064	4.30533983	1.6673E-05	0.001002586	Cluster 3
SAOUHSC_R0008		3921508			69620.64689	3.558593307	0.594901723	4.300867196	1.70131E-05	0.001002586	Cluster 4
SAOUHSC_02388	YP_500865.1	3919605		Uncharacterized protein	282.6875585	2.223808179	0.285076901	4.292905435	1.7635E-05	0.001015616	Cluster 1
SAOUHSC_00502	YP_499075.1	3920414	ctsR	Transcriptional regulator CtsR	1530.909897	2.921469134	0.449984888	4.270074806	1.95407E-05	0.001100361	Cluster 4
SAOUHSC_R0007		3921507			66776.61741	3.561888683	0.601083155	4.262120242	2.02496E-05	0.001115491	Cluster 4
SAOUHSC_R0006		3921506			67433.66198	3.57185231	0.604214401	4.256522696	2.07631E-05	0.00111944	Cluster 4
SAOUHSC_R0009		3921509			71300.89265	3.556547631	0.602510963	4.243155378	2.20399E-05	0.001163522	Cluster 4
SAOUHSC_R00010		3921510			70828.02431	3.554002778	0.604272668	4.226573387	2.37277E-05	0.001204727	Cluster 4
SAOUHSC_02662	YP_501124.1	3921224		PTS system sucrose-specific IIBC component (EC 2.7.1.69)	62.40374969	2.279170132	0.302679032	4.226160377	2.37713E-05	0.001204727	Cluster 3
SAOUHSC_00503	YP_499076.1	3920415	mcsA	Protein-arginine kinase activator protein	2357.781334	2.964605982	0.473659222	4.147720322	3.35802E-05	0.001638505	Cluster 4
SAOUHSC_02381	YP_500858.1	3919424		Uncharacterized protein	2375.387884	3.361033358	0.569276992	4.147424524	3.36236E-05	0.001638505	Cluster 4
SAOUHSC_00313	YP_498903.1	3919540		Uncharacterized protein	240.6852603	-3.454628714	0.601789685	-4.078881333	4.52529E-05	0.002163602	Cluster 2
SAOUHSC_02828	YP_501287.1	3921267		Uncharacterized protein	90.8723175	2.296317197	0.32531102	3.984854849	6.75214E-05	0.003168504	Cluster 3

SAOUHSC_00310	YP_498900.1	3919537	ulaA	Uncharacterized protein	216.9817801	-3.42591041	0.611448233	- 3.967482903	7.26357E-05	0.003346526	Cluster 2
SAOUHSC_00505	YP_499078.1	3920417	clpC	ATP-dependent Clp protease ATP-binding subunit ClpC	7349.920777	2.742751214	0.442656402	3.937029275	8.24965E-05	0.003732969	Cluster 4
SAOUHSC_00504	YP_499077.1	3920416	mcsB	Protein-arginine kinase (EC 2.7.14.1)	3077.381024	2.866923099	0.475601829	3.925390914	8.6589E-05	0.003849413	Cluster 4
SAOUHSC_02872	YP_501327.1	3921543		Uncharacterized protein	310.2962172	3.269290676	0.588128452	3.85849497	0.000114087	0.00498444	Cluster 4
SAOUHSC_00927	YP_499480.1	3920756		Oligopeptide ABC transporter, substrate-binding protein, putative	854.6636938	-3.135553995	0.556847101	- 3.835081467	0.000125523	0.005391093	Cluster 2
SAOUHSC_01972	YP_500469.1	3920448	prsA	Foldase protein PrsA (EC 5.2.1.8)	1193.009807	2.231521803	0.32369605	3.804562346	0.000142055	0.005999462	Cluster 4
SAOUHSC_00912	YP_499465.1	3920799	clpB	Chaperone protein ClpB	8776.310893	3.168265251	0.57416431	3.776384585	0.000159121	0.006610053	Cluster 4
SAOUHSC_00032	YP_498638.1	3919300		Uncharacterized protein	33.6156308	2.67299354	0.448320604	3.731690057	0.000190199	0.007773635	Cluster 4
SAOUHSC_00738	YP_499298.1	3920983		Uncharacterized protein	856.1955357	-2.470331352	0.3948774	- 3.723513556	0.000196469	0.007778957	Cluster 2
SAOUHSC_00926	YP_499479.1	3920813		Oligopeptide ABC transporter, ATP-binding protein, putative	456.0750262	-3.124445348	0.570392495	- 3.724532433	0.000195678	0.007778957	Cluster 2
SAOUHSC_00934	YP_499487.1	3920763	spxA	Regulatory protein Spx	6561.84081	1.916051639	0.247130141	3.706758055	0.000209929	0.008184014	Cluster 4
SAOUHSC_00141	YP_498740.1	3919849		Uncharacterized protein	189.1047772	2.204731853	0.325489933	3.701287602	0.000214508	0.008235814	Cluster 3
SAOUHSC_01684	YP_500194.1	3921796	grpE	Protein GrpE (HSP-70 cofactor)	5875.842759	3.011706604	0.546048292	3.684118485	0.000229495	0.008679724	Cluster 4
SAOUHSC_00881	YP_499434.1	3919228		Uncharacterized protein	170.6608336	1.825222703	0.225379213	3.661485426	0.000250757	0.009344391	Cluster 3
SAOUHSC_01683	YP_500193.1	3921795	dnaK	Chaperone protein DnaK (HSP70) (Heat shock 70 kDa protein) (Heat shock protein 70)	16075.21404	2.896963212	0.521412948	3.638120649	0.000274635	0.009941779	Cluster 4
SAOUHSC_01685	YP_500195.1	3921797	hrcA	Heat-inducible transcription repressor HrcA	6738.49447	3.00465431	0.550735197	3.639960401	0.00027268	0.009941779	Cluster 4
SAOUHSC_02452	YP_500919.1	3919015	lacD	Tagatose 1,6-diphosphate aldolase (EC 4.1.2.40) (D-tagatose-1,6-bisphosphate aldolase) (Tagatose-bisphosphate aldolase)	34.63938198	2.508733157	0.424625386	3.553092226	0.000380731	0.013588342	Cluster 4
SAOUHSC_02595	YP_501057.1	3921591		Uncharacterized protein	148.3689377	-3.053861602	0.587280409	- 3.497241812	0.000470096	0.01654475	Cluster 6
SAOUHSC_00935	YP_499488.1	3920764	mecA	Adapter protein MecA	1596.810727	1.46345548	0.13411367	3.455691585	0.000548883	0.019053006	Cluster 3
SAOUHSC_02883	YP_501337.1	3921553	ssaA	Staphylococcal secretory antigen SsaA	82.82713299	-3.850146866	0.835060131	- 3.413103751	0.000642275	0.021993574	Cluster 6
SAOUHSC_02454	YP_500921.1	3919017	lacB	Galactose-6-phosphate isomerase subunit LacB (EC 5.3.1.26)	14.21593732	3.011823725	0.59223314	3.397013083	0.000681257	0.023017406	Cluster 4
SAOUHSC_02389	YP_500866.1	3919606		Cation efflux family protein, putative	889.8649451	1.954826896	0.283088366	3.372893452	0.000743827	0.024800768	Cluster 1
SAOUHSC_00010	YP_498618.1	3919183		Uncharacterized protein	192.6291625	-2.907576561	0.566898175	- 3.364936851	0.000765612	0.024872568	Cluster 6
SAOUHSC_02888	YP_501341.1	3921557		Uncharacterized protein	516.0139484	-2.918863315	0.569970524	- 3.366600965	0.000761007	0.024872568	Cluster 6
SAOUHSC_02108	YP_500600.1	3921180	ftnA	Bacterial non-heme ferritin (EC 1.16.3.2)	744.5017834	2.86338007	0.554521161	3.360340779	0.000778464	0.024969968	Cluster 4
SAOUHSC_00233	YP_498828.1	3920308	lrgB	Antiholin-like protein LrgB	177.2970696	-2.816749617	0.542373442	- 3.349628646	0.0008092	0.0256314	Cluster 2
SAOUHSC_00373	YP_498962.1	3919792		Xanthine permease, putative	402.4248002	-2.264762019	0.381370522	- 3.316360198	0.000911982	0.028530401	Cluster 6
SAOUHSC_01364	YP_499891.1	3920069	tyrA	Prephenate dehydrogenase (PDH) (EC 1.3.1.12)	293.8611357	1.591248984	0.179230535	3.298818385	0.000970927	0.030004014	Cluster 3
SAOUHSC_01769	YP_500274.1	3919687		Uncharacterized protein	337.9245051	1.706651546	0.218216444	3.238305657	0.001202419	0.03671	Cluster 3
SAOUHSC_01761	YP_500266.1	3920559		Uncharacterized protein	682.2229466	2.955539182	0.612777567	3.19127084	0.001416484	0.042730609	Cluster 3
SAOUHSC_01760	YP_500265.1	3920558		Uncharacterized protein	330.7768048	2.952654097	0.615014702	3.17497141	0.001498513	0.044673306	Cluster 3

S. aureus – 16 hpi vs. inoculum

Locus_id	Protein_id	Gene_id	Name	Description	baseMean	log2FoldChange	lfcSE	stat	pvalue	padj	Cluster
SAOUHSC_00187	YP_498784.1	3919501	pflB	Formate acetyltransferase (EC 2.3.1.54) (Pyruvate formate-lyase)	3453.909064	-7.291625507	0.30079243	-20.91683458	3.76275E-97	9.92991E-94	Cluster 1
SAOUHSC_00898	YP_499451.1	3921744	argH	Argininosuccinate lyase (ASAL) (EC 4.3.2.1) (Arginosuccinase)	560.8832089	4.789305211	0.186482824	20.31986184	8.58175E-92	1.13236E-88	Cluster 4
SAOUHSC_00899	YP_499452.1	3921745	argG	Argininosuccinate synthase (EC 6.3.4.5) (Citrulline--aspartate ligase)	475.9186851	5.324502745	0.215743109	20.04468538	2.24597E-89	1.97571E-86	Cluster 4
SAOUHSC_01788	YP_500293.1	3920430	thrS	Threonine--tRNA ligase (EC 6.1.1.3) (Threonyl-tRNA synthetase) (ThrRS)	3281.621039	-5.772818739	0.293892543	-16.24001306	2.62868E-59	1.73427E-56	Cluster 1
SAOUHSC_00036	YP_498642.1	3919304		Uncharacterized protein	645.3304841	4.542524684	0.220571393	16.06067149	4.81312E-58	2.54037E-55	Cluster 4
SAOUHSC_02582	YP_501044.1	3921578		Putative formate dehydrogenase SAOUHSC_02582 (EC 1.17.1.9)	3000.265637	-2.953491274	0.127524133	-15.31860065	5.74409E-53	2.52644E-50	Cluster 1
SAOUHSC_00608	YP_499171.1	3918927	adh	Alcohol dehydrogenase (ADH) (EC 1.1.1.1)	1667.490482	-7.22113045	0.429679359	-14.47854155	1.65573E-47	6.24211E-45	Cluster 1
SAOUHSC_00173	YP_498770.1	3919487	azoR	FMN-dependent NADH-azoreductase (EC 1.7.1.17) (Azo-dye reductase) (FMN-dependent NADH-azo compound oxidoreductase)	258.5358615	5.414821471	0.307261849	14.36827086	8.18494E-47	2.70001E-44	Cluster 4
SAOUHSC_00965	YP_499518.1	3920676		Uncharacterized protein	441.4206821	3.089961932	0.147739452	14.14626836	1.96934E-45	5.77453E-43	Cluster 5
SAOUHSC_00033	YP_498639.1	3919301		Probable membrane transporter protein	280.0107598	3.956182168	0.222680866	13.27542063	3.21461E-40	8.48335E-38	Cluster 4
SAOUHSC_00188	YP_498785.1	3919502	pflA	Pyruvate formate-lyase-activating enzyme (PFL-activating enzyme) (EC 1.97.1.4)	1444.762665	-7.456708203	0.499129187	-12.93594598	2.82129E-38	6.76853E-36	Cluster 1
SAOUHSC_01450	YP_499971.1	3920227		Uncharacterized protein	1612.478806	-8.19068736	0.557982551	-12.88693947	5.33181E-38	1.17255E-35	Cluster 1
SAOUHSC_01389	YP_499916.1	3920680	pstS	Phosphate-binding protein PstS (PBP)	42.29841808	8.014778491	0.556312302	12.60942544	1.87354E-36	3.80328E-34	Cluster 4
SAOUHSC_00034	YP_498640.1	3919302		Uncharacterized protein	131.1252476	4.393238469	0.276422913	12.27553257	1.22583E-34	2.31068E-32	Cluster 4
SAOUHSC_01170	YP_499709.1	3920921	carB	Carbamoyl-phosphate synthase large chain (EC 6.3.5.5) (Carbamoyl-phosphate synthetase ammonia chain)	835.1016322	-4.332511245	0.279312146	-11.93113617	8.14541E-33	1.43305E-30	Cluster 1
SAOUHSC_R0004		3921504			55386.13645	7.211094616	0.539513585	11.51239707	1.14258E-30	1.88454E-28	Cluster 4
SAOUHSC_R0001		3921501			49146.54944	7.182664294	0.549592044	11.24955202	2.32771E-29	3.61343E-27	Cluster 4
SAOUHSC_R0002		3921502			52568.75687	7.084089414	0.545574052	11.15172063	7.02349E-29	1.02972E-26	Cluster 4
SAOUHSC_R0003		3921503			49318.74763	7.075547334	0.54692502	11.10855623	1.13988E-28	1.58323E-26	Cluster 4
SAOUHSC_R0005		3921505			47135.17388	7.101433817	0.550452197	11.0844027	1.49344E-28	1.97059E-26	Cluster 4
SAOUHSC_00035	YP_498641.1	3919303		Uncharacterized protein	411.0784292	5.495095379	0.406079213	11.06950376	1.76375E-28	2.21644E-26	Cluster 4
SAOUHSC_02581	YP_501043.1	3921577		Uncharacterized protein	707.0486018	-3.241264979	0.205215069	-10.92154191	9.09387E-28	1.09085E-25	Cluster 1
SAOUHSC_02648	YP_501110.1	3921210		L-lactate permease	2186.078447	-3.991687305	0.278932076	-10.72550473	7.72641E-27	8.86521E-25	Cluster 1
SAOUHSC_02942	YP_501394.1	3921648		Anaerobic ribonucleoside-triphosphate reductase, putative (EC 1.17.4.2)	643.4967752	-6.159314161	0.488586269	-10.55967899	4.58226E-26	5.03858E-24	Cluster 1
SAOUHSC_01448	YP_499970.1	3920226	norB	Quinolone resistance protein NorB	1179.551836	-7.128835583	0.582529474	-10.5210738	6.90817E-26	7.29227E-24	Cluster 1
SAOUHSC_00964	YP_499517.1	3920675		Uncharacterized protein	187.2078154	3.14608899	0.208493676	10.29330498	7.55382E-25	7.66713E-23	Cluster 5

SAOUHSC_02255	YP_500737.1	3919675	groES	10 kDa chaperonin Cpn10 GroES forms homoheptameric ring binds to one or both ends of the GroEL double barrel in the presence of adenine nucleotides capping it folding of unfolded substrates initiates in a GroEL-substrate bound and capped by GroES release of the folded substrate is dependent on ATP binding and hydrolysis in the trans ring	1099.207829	5.621224666	0.458679614	10.07506007	7.12173E-24	6.96083E-22	Cluster 4
SAOUHSC_02941	YP_501393.1	3921647		Anaerobic ribonucleoside-triphosphate reductase-activating protein (EC 1.97.1.-)	455.5980357	-6.190400092	0.520143093	-9.978792683	1.88749E-23	1.77896E-21	Cluster 1
SAOUHSC_01158	YP_499698.1	3920716		Uncharacterized protein	422.6660128	2.363099291	0.137920148	9.883249886	4.92105E-23	4.47816E-21	Cluster 5
SAOUHSC_01889	YP_500391.1	3920837		Riboflavin biosynthesis protein RibD [Includes: Diaminohydroxyphosphoribosylaminopyrimidine deaminase (DRAP deaminase) (EC 3.5.4.26) (Riboflavin-specific deaminase); 5-amino-6-(5-phosphoribosylamino)uracil reductase (EC 1.1.1.193) (HTP reductase)]	259.200404	3.788335484	0.283511711	9.834992264	7.95737E-23	6.99984E-21	Cluster 5
SAOUHSC_T00042		3921031			78.04391206	3.979507657	0.303411232	9.820030834	9.23153E-23	7.85871E-21	Cluster 5
SAOUHSC_01451	YP_499972.1	3920228	tdcB	L-threonine dehydratase catabolic TdcB (EC 4.3.1.19) (Threonine deaminase)	609.8332947	-7.821619972	0.697966249	-9.773567109	1.46212E-22	1.20579E-20	Cluster 1
SAOUHSC_02887	YP_501340.1	3921556	isaA	Probable transglycosylase IsaA (EC 3.2.-.-) (Immunodominant staphylococcal antigen A)	1074.42361	3.976249852	0.306877479	9.698495519	3.05977E-22	2.44689E-20	Cluster 5
SAOUHSC_00206	YP_498803.1	3920362	ldh1	L-lactate dehydrogenase 1 (L-LDH 1) (EC 1.1.1.27)	2101.398789	-5.250718553	0.448471444	-9.478236823	2.58616E-21	2.00731E-19	Cluster 1
SAOUHSC_02258	YP_500740.1	3919678		Uncharacterized protein	383.6338158	3.34977833	0.255781654	9.186657026	4.05239E-20	3.0555E-18	Cluster 4
SAOUHSC_01262	YP_499795.2	3919993	recA	Protein RecA (Recombinase A)	2059.139015	2.102605239	0.120682739	9.136395581	6.45685E-20	4.73323E-18	Cluster 4
SAOUHSC_02400	YP_500876.1	3919615		PTS system, mannitol-specific component, putative	382.8435125	-5.02970666	0.441353795	-9.130331961	6.82896E-20	4.84745E-18	Cluster 1
SAOUHSC_02757	YP_501216.1	3921629		Antitoxin	411.1625427	3.468137075	0.270392983	9.127962745	6.98004E-20	4.84745E-18	Cluster 4
SAOUHSC_00659	YP_499217.1	3919949		Uncharacterized protein	1617.640665	2.411204277	0.156432694	9.021159476	1.86109E-19	1.25934E-17	Cluster 4
SAOUHSC_R0008		3921508			69620.64689	6.313845126	0.594874078	8.932722601	4.15651E-19	2.74226E-17	Cluster 4
SAOUHSC_00437	YP_499020.1	3919109		Uncharacterized protein	1198.814839	3.5623794	0.290262328	8.827805599	1.06749E-18	6.87102E-17	Cluster 4
SAOUHSC_02756	YP_501215.1	3921628		Uncharacterized protein	437.5758361	3.356980188	0.270087167	8.726738917	2.62121E-18	1.647E-16	Cluster 4
SAOUHSC_R0007		3921507			66576.61741	6.240411567	0.601053574	8.718709597	2.8139E-18	1.72695E-16	Cluster 4
SAOUHSC_03022	YP_501471.1	3921289		UPF0312 protein SAOUHSC_03022	265.3923101	3.27864183	0.262533694	8.679426243	3.9777E-18	2.38572E-16	Cluster 4
SAOUHSC_R0006		3921506			67433.66198	6.236861081	0.604185038	8.667644427	4.41152E-18	2.58711E-16	Cluster 4
SAOUHSC_02381	YP_500858.1	3919424		Uncharacterized protein	2375.387884	5.899392433	0.568452343	8.618827057	6.76434E-18	3.88067E-16	Cluster 4
SAOUHSC_01327	YP_499857.1	3920192	katA	Catalase (EC 1.11.1.6)	2026.604821	2.543174752	0.179457184	8.599124967	8.03262E-18	4.51023E-16	#N/A
SAOUHSC_01021	YP_499570.1	3920281		Uncharacterized protein	846.8312136	-4.360210915	0.392949293	-8.551258325	1.21754E-17	6.69392E-16	Cluster 1
SAOUHSC_00561	YP_499133.1	3920606		Uncharacterized protein	9001.938896	5.024771333	0.471778883	8.531054435	1.4502E-17	7.81035E-16	Cluster 4
SAOUHSC_02401	YP_500877.1	3919616		Uncharacterized protein	367.9558937	-3.774319079	0.327493486	-8.471371782	2.4252E-17	1.28002E-15	Cluster 1
SAOUHSC_R0009		3921509			71300.89265	6.099594007	0.602481995	8.464309395	2.57675E-17	1.33334E-15	Cluster 4
SAOUHSC_R00010		3921510			70828.02431	6.112700779	0.604243842	8.461320454	2.64367E-17	1.34166E-15	Cluster 4

SAOUHSC_02676	YP_501138.1	3921238	nreB	Oxygen sensor histidine kinase NreB (EC 2.7.13.3) (Nitrogen regulation protein B)	422.1198357	-3.258671078	0.267178347	-8.453795392	2.81983E-17	1.40406E-15	Cluster 1
SAOUHSC_00699	YP_499258.1	3921003		Deoxyribodipyrimidine photolyase, putative (EC 4.1.99.3)	387.2192165	-3.267764604	0.268359459	-8.450473901	2.90123E-17	1.41784E-15	Cluster 1
SAOUHSC_00694	YP_499253.1	3920998		conserved hypothetical protein	1498.578012	1.83718122	0.099436061	8.419291858	3.78767E-17	1.78494E-15	Cluster 5
SAOUHSC_02254	YP_500736.1	3919674	groEL	60 kDa chaperone family promotes refolding of misfolded polypeptides especially under stressful conditions forms two stacked rings of heptamers to form a barrel-shaped 14mer ends can be capped by GroES misfolded proteins enter the barrel where they are refolded when GroES binds many bacteria have multiple copies of the groEL gene which are active under different environmental conditions the B.japonicum protein in this cluster is expressed constitutively in Rhodobacter Corynebacterium and Rhizobium this protein is essential for growth	6220.093592	5.097591386	0.486585347	8.421115448	3.72917E-17	1.78494E-15	Cluster 4
SAOUHSC_01452	YP_499973.1	3919897	ald1	Alanine dehydrogenase 1 (EC 1.4.1.1)	347.9550163	-7.987005738	0.834116112	-8.376538512	5.45071E-17	2.52358E-15	Cluster 1
SAOUHSC_00174	YP_498771.1	3919488		M23/M37 peptidase domain protein	83.6685762	3.952420646	0.354789482	8.321612652	8.67745E-17	3.94824E-15	Cluster 4
SAOUHSC_01063	YP_499609.1	3921725		Uncharacterized protein	295.2127897	2.371455248	0.166976153	8.213479727	2.14869E-16	9.61084E-15	Cluster 5
SAOUHSC_01394	YP_499921.1	3920684		Aspartokinase (EC 2.7.2.4)	1244.680427	4.516394268	0.42869965	8.202465913	2.35506E-16	1.03583E-14	Cluster 4
SAOUHSC_02606	YP_501067.1	3921384	hutI	Imidazolonepropionase (EC 3.5.2.7) (Imidazolone-5-propionate hydrolase)	289.3519664	-4.295815297	0.404875444	-8.140319064	3.94238E-16	1.70556E-14	Cluster 1
SAOUHSC_00330	YP_498919.1	3919595		Uncharacterized protein	69.58169408	3.842046935	0.35105286	8.095780614	5.68983E-16	2.42185E-14	Cluster 5
SAOUHSC_T00055		3921044			79.92043684	3.180413173	0.270644867	8.056362551	7.85983E-16	3.29239E-14	Cluster 5
SAOUHSC_01022	YP_499571.1	3920282		Uncharacterized protein	322.3066625	-4.599259915	0.451033211	-7.980033021	1.46294E-15	6.03235E-14	Cluster 1
SAOUHSC_T00038		3921164			50.99544624	4.171380271	0.3987422	7.953460336	1.81373E-15	7.36374E-14	Cluster 5
SAOUHSC_02781	YP_501241.1	3921436		Uncharacterized protein	244.7367433	-5.16831203	0.52452276	-7.946865887	1.91289E-15	7.64867E-14	Cluster 1
SAOUHSC_T00032		3921158			95.21462325	3.281115876	0.291062971	7.837190229	4.6074E-15	1.81476E-13	Cluster 5
SAOUHSC_02742	YP_501203.1	3921616		Amino acid transporter, putative	244.4065731	-3.617460573	0.336926465	-7.768640467	7.9333E-15	3.07882E-13	Cluster 1
SAOUHSC_01888	YP_500390.1	3920836		Riboflavin synthase, alpha subunit (EC 2.5.1.9)	132.0296318	3.177397255	0.281837882	7.725708276	1.11233E-14	4.19349E-13	Cluster 5
SAOUHSC_02877	YP_501332.1	3921548	crtN	4,4'-diapophytoene desaturase (4,4'-diaponeurosporene-forming) (EC 1.3.8.-) (Dehydrosqualene desaturase)	339.0115394	-3.388920744	0.309168834	-7.726913192	1.10186E-14	4.19349E-13	Cluster 1
SAOUHSC_00880	YP_499433.1	3919227		Uncharacterized protein	359.4019252	2.093408365	0.142973334	7.647638427	2.04704E-14	7.52785E-13	Cluster 4
SAOUHSC_02680	YP_501142.1	3921242		Nitrate reductase, beta subunit (EC 1.7.99.4)	234.5113005	-7.29274308	0.822880629	-7.64721256	2.05383E-14	7.52785E-13	Cluster 1
SAOUHSC_02069	YP_500562.1	3919756		Phi PV83 orf 20-like protein	13.03754976	8.095650293	0.930546414	7.625251342	2.43559E-14	8.80484E-13	Cluster 5
SAOUHSC_01041	YP_499590.1	3919889		Pyruvate dehydrogenase complex, E1 component, pyruvate dehydrogenase beta subunit, putative (EC 1.2.4.1)	2175.360617	-2.406939894	0.18474511	-7.615573134	2.62524E-14	9.36217E-13	Cluster 1
SAOUHSC_00502	YP_499075.1	3920414	ctsR	Transcriptional regulator CtsR	1530.909897	4.398841261	0.448302352	7.581582481	3.41365E-14	1.20115E-12	Cluster 4

SAOUHSC_00500	YP_499073.1	3920412	pdxT	Pyridoxal 5'-phosphate synthase subunit PdxT (EC 4.3.3.6) (Pdx2) (Pyridoxal 5'-phosphate synthase glutaminase subunit) (EC 3.5.1.2)	370.0934595	-3.132665424	0.284227015	-7.503387482	6.21894E-14	2.15944E-12	Cluster 1
SAOUHSC_01172	YP_499711.1	3920923	pyrE	Orotate phosphoribosyltransferase (OPRT) (OPRTase) (EC 2.4.2.10)	241.2461376	-4.224719412	0.42999293	-7.499470784	6.4076E-14	2.19606E-12	Cluster 1
SAOUHSC_00037	YP_498643.1	3919305		Uncharacterized protein	459.301566	2.192228614	0.159617891	7.469266824	8.06429E-14	2.69388E-12	Cluster 4
SAOUHSC_01040	YP_499589.1	3919888	pdhA	Pyruvate dehydrogenase E1 component subunit alpha (EC 1.2.4.1)	2002.427025	-2.334982378	0.178722144	-7.469596946	8.04408E-14	2.69388E-12	Cluster 1
SAOUHSC_01574	YP_500089.1	3920168		Helix-turn-helix domain protein	20.94457146	5.982350798	0.667638215	7.462650711	8.47989E-14	2.78845E-12	Cluster 4
SAOUHSC_02681	YP_501143.1	3921243		Nitrate reductase, alpha subunit (EC 1.7.99.4)	313.2090376	-6.505539583	0.737866336	-7.461432128	8.5587E-14	2.78845E-12	Cluster 1
SAOUHSC_00569	YP_499138.1	3920611		Uncharacterized protein	1268.478725	2.149267684	0.155097335	7.409976991	1.26321E-13	4.06539E-12	Cluster 5
SAOUHSC_T00022		3921148			63.99701988	3.283087252	0.309533706	7.375892223	1.63248E-13	5.1905E-12	Cluster 5
SAOUHSC_01171	YP_499710.1	3920922	pyrF	Orotidine 5'-phosphate decarboxylase (EC 4.1.1.23) (OMP decarboxylase) (OMPDCase) (OMPdecase)	224.6730257	-4.241265113	0.441570121	-7.340318018	2.13087E-13	6.69448E-12	Cluster 1
SAOUHSC_01019	YP_499569.1	3920280		Uncharacterized protein	445.9257373	-4.686556939	0.50350145	-7.321839761	2.44594E-13	7.50563E-12	Cluster 1
SAOUHSC_02467	YP_500935.1	3919030		Alpha-acetolactate decarboxylase (EC 4.1.1.5)	296.6826607	-7.320920875	0.863264255	-7.322115842	2.44091E-13	7.50563E-12	Cluster 1
SAOUHSC_T00026		3921152			98.20922998	3.468423737	0.338746756	7.286929528	3.17099E-13	9.61866E-12	Cluster 5
SAOUHSC_02468	YP_500936.1	3919031		Acetolactate synthase, putative (EC 2.2.1.6)	580.4930772	-4.698095401	0.510545621	-7.243418113	4.37515E-13	1.31205E-11	Cluster 1
SAOUHSC_T0009		3921328			70.8523646	3.455273155	0.340842015	7.203551929	5.86639E-13	1.73948E-11	Cluster 5
SAOUHSC_02770	YP_501229.1	3921425		Uncharacterized protein	26.30339593	4.427204982	0.476802308	7.187895123	6.57978E-13	1.92934E-11	Cluster 5
SAOUHSC_03015	YP_501464.1	3921496	hisZ	ATP phosphoribosyltransferase regulatory subunit	97.88657742	4.415338122	0.476854988	7.162215361	7.93836E-13	2.30212E-11	Cluster 4
SAOUHSC_02452	YP_500919.1	3919015	lacD	Tagatose 1,6-diphosphate aldolase (EC 4.1.2.40) (D-tagatose-1,6-bisphosphate aldolase) (Tagatose-bisphosphate aldolase)	34.63938198	3.629220436	0.368811791	7.12889473	1.01178E-12	2.90227E-11	Cluster 4
SAOUHSC_T00033		3921159			85.13653836	3.080540147	0.292056409	7.123761307	1.05021E-12	2.98011E-11	Cluster 5
SAOUHSC_02607	YP_501068.1	3921385	hutU	Urocanate hydratase (Urocanase) (EC 4.2.1.49) (Imidazolonepropionate hydrolase)	375.1239097	-4.712296365	0.521280653	-7.121492698	1.06764E-12	2.99735E-11	Cluster 1
SAOUHSC_T00045		3921034			84.24058298	3.689135405	0.379450007	7.086929389	1.3712E-12	3.80906E-11	Cluster 5
SAOUHSC_00420	YP_499005.1	3920577		Transporter	187.1314183	3.335174925	0.329658048	7.083627828	1.40429E-12	3.86033E-11	Cluster 5
SAOUHSC_02589	YP_501051.1	3921585		Uncharacterized protein	783.60849	-2.55967295	0.221158885	-7.052273521	1.76018E-12	4.78877E-11	Cluster 1
SAOUHSC_02710	YP_501172.1	3919729	hlgB	Gamma-hemolysin component B (H-gamma-1) (H-gamma-I) (Leukocidin f subunit)	205.5472279	-4.285544494	0.466776158	-7.03880101	1.93901E-12	5.22148E-11	Cluster 1
SAOUHSC_00183	YP_498780.1	3919497	uhpT	Membrane protein, putative	136.6012116	2.731453416	0.248735482	6.961023013	3.37811E-12	8.91482E-11	#N/A
SAOUHSC_01887	YP_500389.1	3920835	ribBA	Riboflavin biosynthesis protein RibBA [Includes: 3,4-dihydroxy-2-butanone 4-phosphate synthase (DHBP synthase) (EC 4.1.99.12); GTP cyclohydrolase-2 (EC 3.5.4.25) (GTP cyclohydrolase II)]	245.3414198	3.05671427	0.295448739	6.961323557	3.37091E-12	8.91482E-11	Cluster 5
SAOUHSC_02881	YP_501335.1	3921551		conserved hypothetical protein	312.188641	-3.485575906	0.357232675	-6.957862706	3.45474E-12	9.02678E-11	Cluster 2
SAOUHSC_T00051		3921040			92.05049266	3.747092623	0.39544436	6.946849933	3.73533E-12	9.57041E-11	Cluster 5
SAOUHSC_02425	YP_500896.1	3919635		UPF0457 protein SAOUHSC_02425	203.7979786	3.781059055	0.400327555	6.94695886	3.73244E-12	9.57041E-11	Cluster 4

SAOUHSC_01395	YP_499922.1	3920685	asd	Aspartate-semialdehyde dehydrogenase (ASA dehydrogenase) (ASADH) (EC 1.2.1.11) (Aspartate-beta-semialdehyde dehydrogenase)	1297.923696	4.032385806	0.436726003	6.943451474	3.82634E-12	9.70934E-11	Cluster 4
SAOUHSC_00503	YP_499076.1	3920415	mcsA	Protein-arginine kinase activator protein	2357.781334	4.262381777	0.472575868	6.903403244	5.07712E-12	1.27605E-10	Cluster 4
SAOUHSC_01399	YP_499926.1	3920689		Uncharacterized hydrolase SAOUHSC_01399 (EC 3.-.-.-)	534.9037572	3.71301494	0.394918547	6.86980887	6.4288E-12	1.60053E-10	Cluster 4
SAOUHSC_01573	YP_500088.1	3920167		Conserved hypothetical phage protein	9.605507353	5.818702296	0.702727004	6.857146903	7.02494E-12	1.7326E-10	#N/A
SAOUHSC_01684	YP_500194.1	3921796	grpE	Protein GrpE (HSP-70 cofactor)	5875.842759	4.73956796	0.545700048	6.85279024	7.24232E-12	1.76968E-10	Cluster 4
SAOUHSC_00790	YP_499347.1	3919354	clpP	ATP-dependent Clp protease proteolytic subunit (EC 3.4.21.92) (Endopeptidase Clp)	1494.497821	3.034729031	0.297651103	6.835953273	8.14615E-12	1.97226E-10	#N/A
SAOUHSC_02692	YP_501154.1	3919711		Antitoxin	134.3801745	2.655261961	0.242512254	6.825477607	8.76333E-12	2.1024E-10	Cluster 4
SAOUHSC_02684	YP_501146.1	3919703		Assimilatory nitrite reductase [NAD(P)H], large subunit, putative (EC 1.7.1.4)	152.5017622	-4.94194969	0.578701082	-6.811719923	9.64387E-12	2.29281E-10	Cluster 1
SAOUHSC_02687	YP_501149.1	3919706		Formate/nitrite transporter, putative	172.6370433	-4.328804093	0.494926327	-6.725857799	1.74561E-11	4.11309E-10	Cluster 1
SAOUHSC_02691	YP_501153.1	3919710		Uncharacterized protein	179.1619839	2.812267374	0.2698416	6.71604145	1.86728E-11	4.36084E-10	Cluster 4
SAOUHSC_01685	YP_500195.1	3921797	hrcA	Heat-inducible transcription repressor HrcA	6738.49447	4.685513689	0.550433761	6.695653412	2.1471E-11	4.97035E-10	Cluster 4
SAOUHSC_02678	YP_501140.1	3921240		Respiratory nitrate reductase, gamma subunit (EC 1.7.99.4)	183.9806435	-3.47980819	0.370745627	-6.688705158	2.25154E-11	5.16679E-10	Cluster 1
SAOUHSC_01042	YP_499591.1	3919890		Dihydrolipoamide acetyltransferase component of pyruvate dehydrogenase complex (EC 2.3.1.-)	2923.992131	-2.219015805	0.18297232	-6.662296276	2.69582E-11	6.13299E-10	Cluster 1
SAOUHSC_01168	YP_499707.1	3920919	pyrC	Dihydroorotase (DHOase) (EC 3.5.2.3)	339.0616685	-3.186488177	0.32906982	-6.644450648	3.0435E-11	6.86478E-10	Cluster 1
SAOUHSC_00132	YP_498732.1	3919841	aldA	Putative aldehyde dehydrogenase AldA (EC 1.2.1.3)	842.6101151	2.983648628	0.29944068	6.624512826	3.48395E-11	7.79165E-10	Cluster 4
SAOUHSC_01169	YP_499708.1	3920920	carA	Carbamoyl-phosphate synthase small chain (EC 6.3.5.5) (Carbamoyl-phosphate synthetase glutamine chain)	304.2600492	-3.590403215	0.391667359	-6.613783752	3.74619E-11	8.30772E-10	Cluster 1
SAOUHSC_02880	YP_501334.1	3921550	crtQ	4,4'-diaponeurosporenoate glycosyltransferase (EC 2.4.1.-)	299.1857395	-3.912102271	0.441122343	-6.601575084	4.06812E-11	8.94648E-10	Cluster 2
SAOUHSC_02677	YP_501139.1	3921239		Uncharacterized protein	247.2836012	-3.209115932	0.335243311	-6.58958989	4.41043E-11	9.61911E-10	Cluster 1
SAOUHSC_03014	YP_501463.1	3921495	hisG	ATP phosphoribosyltransferase (ATP-PRT) (ATP-PRTase) (EC 2.4.2.17)	54.68089611	4.128573884	0.475112138	6.584916775	4.5514E-11	9.84521E-10	Cluster 4
SAOUHSC_00204	YP_498801.1	3920360		Globin domain protein	158.2295452	-4.175111588	0.482729482	-6.577413866	4.78701E-11	1.02707E-09	Cluster 1
SAOUHSC_02984	YP_501433.1	3921466	gtfA	UDP-N-acetylglucosamine--peptide N-acetylglucosaminyltransferase GtfA subunit (EC 2.4.1.-) (Glycosyltransferase GtfA)	444.1006827	-2.893987534	0.288946774	-6.554797307	5.57175E-11	1.18579E-09	Cluster 1
SAOUHSC_02675	YP_501137.1	3921237	nreC	Oxygen regulatory protein NreC (Nitrogen regulation protein C)	204.587909	-3.307630752	0.352620675	-6.54422987	5.98028E-11	1.26256E-09	Cluster 1
SAOUHSC_00912	YP_499465.1	3920799	clpB	Chaperone protein ClpB	8776.310893	4.744540429	0.573917469	6.524527704	6.82161E-11	1.42875E-09	Cluster 4
SAOUHSC_01683	YP_500193.1	3921795	dnaK	Chaperone protein DnaK (HSP70) (Heat shock 70 kDa protein) (Heat shock protein 70)	16075.21404	4.399621412	0.521281959	6.521655606	6.95355E-11	1.44492E-09	Cluster 4

SAOUHSC_02671	YP_501133.1	3921233		conserved hypothetical protein	157.9785011	-3.778660943	0.427932741	-6.493218853	8.40215E-11	1.73229E-09	Cluster 1
SAOUHSC_02741	YP_501202.1	3921615		Amino acid ABC transporter, permease protein, putative	205.4304399	-3.486428086	0.383048157	-6.491163163	8.51762E-11	1.74248E-09	Cluster 1
SAOUHSC_T00039		3921165			44.51275062	3.654851265	0.410445499	6.468218739	9.91649E-11	2.01305E-09	Cluster 5
SAOUHSC_01361	YP_499888.1	3920066	msrR	Regulatory protein MsrR	154.6052696	2.851397956	0.286778272	6.455851566	1.07612E-10	2.16785E-09	Cluster 5
SAOUHSC_02542	YP_501005.1	3921131		Molybdopterin molybdenumtransferase (EC 2.10.1.1)	219.4669035	-3.191628722	0.34046698	-6.437125632	1.21757E-10	2.43422E-09	Cluster 1
SAOUHSC_00504	YP_499077.1	3920416	mcsB	Protein-arginine kinase (EC 2.7.14.1)	3077.381024	4.040776614	0.474809915	6.404197807	1.51162E-10	2.99937E-09	Cluster 4
SAOUHSC_T00043		3921032			40.74094163	3.46659701	0.391987582	6.292538648	3.12316E-10	6.15075E-09	Cluster 5
SAOUHSC_00365	YP_498954.1	3919784	ahpC	Alkyl hydroperoxide reductase C (EC 1.11.1.15) (Peroxiredoxin) (Thioredoxin peroxidase)	1471.220977	2.871843582	0.298377675	6.273403601	3.5324E-10	6.81374E-09	Cluster 4
SAOUHSC_02679	YP_501141.1	3921241		Respiratory nitrate reductase, delta subunit, putative	101.7287111	-6.349921557	0.852686189	-6.274197503	3.51442E-10	6.81374E-09	Cluster 1
SAOUHSC_02879	YP_501333.1	3921549	crtM	4,4'-diapophytoene synthase (DAP synthase) (EC 2.5.1.96) (C30 carotenoid synthase) (Dehydrosqualene synthase)	193.0345321	-3.24835903	0.358407628	-6.273189672	3.53726E-10	6.81374E-09	Cluster 1
SAOUHSC_01043	YP_499592.1	3919891		Dihydrolipoyl dehydrogenase (EC 1.8.1.4)	2962.782069	-2.160197471	0.185857182	-6.242413976	4.30869E-10	8.23958E-09	Cluster 1
SAOUHSC_01972	YP_500469.1	3920448	prsA	Foldase protein PrsA (EC 5.2.1.8)	1193.009807	2.970808576	0.321312945	6.133610888	8.59065E-10	1.63099E-08	Cluster 4
SAOUHSC_00060	YP_498664.1	3919091		Uncharacterized protein	259.1687166	2.830610197	0.298890126	6.124692788	9.08587E-10	1.71269E-08	Cluster 5
SAOUHSC_02134	YP_500624.1	3921204		Nitric oxide synthase oxygenase (EC 1.14.14.47)	185.2463604	-2.978854269	0.324625984	-6.095797526	1.08893E-09	2.03808E-08	Cluster 1
SAOUHSC_00367	YP_498956.1	3919786		Uncharacterized protein	345.0305849	-2.38058359	0.230585436	-5.987297448	2.13356E-09	3.96512E-08	Cluster 1
SAOUHSC_01156	YP_499697.1	3920715		Uncharacterized protein	208.6075903	2.24162267	0.208430467	5.957011396	2.56892E-09	4.74083E-08	Cluster 5
SAOUHSC_01396	YP_499923.1	3920686	dapA	4-hydroxy-tetrahydrodipicolinate synthase (HTPA synthase) (EC 4.3.3.7)	1112.066085	3.566992508	0.431488281	5.949159269	2.69523E-09	4.93939E-08	#N/A
SAOUHSC_02682	YP_501144.1	3919701		Uroporphyrin-III C-methyltransferase, putative (EC 2.1.1.107)	121.8177407	-5.416148455	0.743706652	-5.938024677	2.88477E-09	5.25027E-08	Cluster 1
SAOUHSC_00009	YP_498617.1	3919182	serS	Serine--tRNA ligase (EC 6.1.1.11) (Seryl-tRNA synthetase) (SerRS) (Seryl-tRNA(Ser/Sec) synthetase)	594.9093392	-3.262910703	0.381571212	-5.930506896	3.02001E-09	5.45877E-08	Cluster 2
SAOUHSC_02709	YP_501171.1	3919728	hlgC	Gamma-hemolysin component C (Leukocidin s subunit)	177.2058191	-4.434385439	0.580276102	-5.918536761	3.24818E-09	5.83126E-08	Cluster 1
SAOUHSC_T0007		3921326			55.89780537	3.405415049	0.406846532	5.912340052	3.37281E-09	6.01409E-08	Cluster 5
SAOUHSC_01979	YP_500476.1	3920455		Uncharacterized protein	74.187767	3.482982974	0.420575043	5.903781064	3.55264E-09	6.29222E-08	Cluster 5
SAOUHSC_02453	YP_500920.1	3919016	lacC	Tagatose-6-phosphate kinase (EC 2.7.1.144) (Phosphotagatokinase)	28.10047883	3.249595269	0.383189433	5.87071322	4.33924E-09	7.63418E-08	#N/A
SAOUHSC_00113	YP_498713.1	3919822		Aldehyde-alcohol dehydrogenase	366.8790855	-4.564006365	0.610243754	-5.840299622	5.2107E-09	9.10665E-08	Cluster 1
SAOUHSC_00505	YP_499078.1	3920417	clpC	ATP-dependent Clp protease ATP-binding subunit ClpC	7349.920777	3.579394007	0.442304411	5.831716673	5.486E-09	9.52471E-08	Cluster 4
SAOUHSC_02743	YP_501204.1	3921617		Amino acid ABC transporter, permease protein, putative	117.5646525	-3.708500941	0.467262261	-5.796532626	6.77E-09	1.16771E-07	Cluster 1

SAOUHSC_00698	YP_499257.1	3921002		Uncharacterized protein	206.7331379	-2.730021293	0.298715019	-5.791544389	6.97421E-09	1.19513E-07	Cluster 1
SAOUHSC_T00019		3921338			54.27864824	3.266304965	0.391660826	5.786396842	7.19122E-09	1.22436E-07	Cluster 5
SAOUHSC_T00034		3921160			11.07899762	5.470713339	0.776547737	5.757164854	8.55384E-09	1.44702E-07	Cluster 5
SAOUHSC_02753	YP_501212.1	3921625		Membrane protein, putative	238.4299286	-2.942470576	0.33980283	-5.716463796	1.08764E-08	1.8282E-07	Cluster 1
SAOUHSC_T00024		3921150			73.98496278	3.106299106	0.36855378	5.715038674	1.09679E-08	1.83192E-07	Cluster 5
SAOUHSC_01973	YP_500470.1	3920449		Uncharacterized protein	521.6832242	-3.377977444	0.416638976	-5.707525169	1.14631E-08	1.90258E-07	#N/A
SAOUHSC_01058	YP_499605.1	3921721		GTP-binding protein TypA, putative	375.897851	2.921049392	0.336651967	5.706336449	1.15434E-08	1.90393E-07	Cluster 5
SAOUHSC_01877	YP_500380.1	3921766		Uncharacterized protein	140.297614	2.323821439	0.232143197	5.702607076	1.17989E-08	1.93399E-07	Cluster 6
SAOUHSC_T0002		3921518			105.5966373	3.242670348	0.394703397	5.681912959	1.33196E-08	2.16979E-07	Cluster 5
SAOUHSC_02683	YP_501145.1	3919702		Assimilatory nitrite reductase [NAD(P)H], small subunit, putative (EC 1.7.1.4)	65.45380496	-5.95160733	0.872323306	-5.676344191	1.37604E-08	2.22783E-07	Cluster 1
SAOUHSC_02449	YP_500916.1	3919012	lacG	6-phospho-beta-galactosidase (EC 3.2.1.85) (Beta-D-phosphogalactoside galactohydrolase) (PGALase) (P-beta-Gal) (PBG)	59.7230505	2.602858292	0.284507406	5.633801649	1.7628E-08	2.8366E-07	#N/A
SAOUHSC_03013	YP_501462.1	3921494	hisD	Histidinol dehydrogenase (HDH) (EC 1.1.1.23)	91.14510917	3.458168434	0.436698341	5.628985058	1.81273E-08	2.89927E-07	Cluster 4
SAOUHSC_01397	YP_499924.1	3920687	dapB	4-hydroxy-tetrahydrodipicolinate reductase (HTPA reductase) (EC 1.17.1.8)	959.5317292	3.468235119	0.439907523	5.610804525	2.01388E-08	3.20159E-07	#N/A
SAOUHSC_01789	YP_500294.1	3920431		Uncharacterized protein	319.8197229	-2.854999438	0.3333908	-5.564039078	2.63601E-08	4.16553E-07	Cluster 1
SAOUHSC_02985	YP_501434.1	3921467	secA2	Protein translocase subunit SecA 2	463.8983183	-2.756051071	0.315885957	-5.559129917	2.71123E-08	4.25889E-07	Cluster 1
SAOUHSC_01689	YP_500199.1	3921801	rpsT	30S ribosomal protein S20	114.04371	2.468993439	0.264866033	5.546175263	2.91986E-08	4.55948E-07	Cluster 5
SAOUHSC_T00030		3921156			48.53235204	3.107699178	0.381201371	5.529096532	3.21884E-08	4.99678E-07	Cluster 5
SAOUHSC_01679	YP_500189.1	3920090		Uncharacterized protein	658.2266933	2.621594573	0.294243217	5.511068669	3.56662E-08	5.50427E-07	Cluster 5
SAOUHSC_01398	YP_499925.1	3920688	dapH	2,3,4,5-tetrahydropyridine-2,6-dicarboxylate N-acetyltransferase (EC 2.3.1.89) (Tetrahydrodipicolinate N-acetyltransferase) (THP acetyltransferase) (Tetrahydropicolinate acetylase)	999.2915039	3.408032376	0.43721483	5.507663994	3.63627E-08	5.56204E-07	#N/A
SAOUHSC_02294	YP_500773.1	3919166		Uncharacterized protein	10.55146907	5.530907557	0.822726843	5.507183336	3.6462E-08	5.56204E-07	Cluster 5
SAOUHSC_00499	YP_499072.1	3920411	pdxS	Pyridoxal 5'-phosphate synthase subunit PdxS (PLP synthase subunit PdxS) (EC 4.3.3.6) (Pdx1)	446.2946157	-2.579258024	0.287198451	-5.498838936	3.823E-08	5.79822E-07	#N/A
SAOUHSC_T00041		3921030			102.110728	3.328030834	0.425133953	5.475993666	4.35063E-08	6.56075E-07	Cluster 5
SAOUHSC_02708	YP_501170.1	3919727		Gamma-hemolysin h-gamma-ii subunit, putative	143.3926004	-4.009216216	0.550099129	-5.470316275	4.49233E-08	6.73595E-07	Cluster 1
SAOUHSC_01009	YP_499559.1	3920270	purK	N5-carboxyaminoimidazole ribonucleotide synthase (N5-CAIR synthase) (EC 6.3.4.18) (5-(carboxyamino)imidazole ribonucleotide synthetase)	75.80217562	2.854844083	0.339263142	5.46727261	4.57013E-08	6.81388E-07	Cluster 5
SAOUHSC_01400	YP_499927.1	3920690		alanine racemase putative	494.6976392	3.148469352	0.394091326	5.45170423	4.98894E-08	7.39652E-07	#N/A
SAOUHSC_00913	YP_499466.1	3920800		Uncharacterized protein	26.48185687	4.734360862	0.68659929	5.438923277	5.36035E-08	7.85917E-07	Cluster 5
SAOUHSC_02241	YP_500725.1	3919663		Uncharacterized leukocidin-like protein 1	218.8247911	-4.641661384	0.669556392	-5.438916612	5.36055E-08	7.85917E-07	Cluster 1
SAOUHSC_T00052		3921041			127.145798	3.202655869	0.405430221	5.432885255	5.54501E-08	8.08468E-07	Cluster 5

SAOUHSC_T00012		3921331			54.87241468	2.887881221	0.349796345	5.39708676	6.77316E-08	9.82109E-07	Cluster 5
SAOUHSC_02345	YP_500822.1	3920968	atpA	ATP synthase subunit alpha (EC 7.1.2.2) (ATP synthase F1 sector subunit alpha) (F-ATPase subunit alpha)	1960.727829	-2.162413041	0.216563166	-5.367547314	7.98146E-08	1.15099E-06	Cluster 1
SAOUHSC_00364	YP_498953.1	3919783	ahpF	Alkyl hydroperoxide reductase subunit F (EC 1.8.1.-)	3968.768593	2.749287566	0.329215118	5.313509227	1.07534E-07	1.54229E-06	Cluster 4
SAOUHSC_00934	YP_499487.1	3920763	spxA	Regulatory protein Spx	6561.84081	2.292406936	0.246603846	5.2408223	1.59863E-07	2.28042E-06	Cluster 4
SAOUHSC_01165	YP_499705.1	3920917		Uracil permease, putative	213.0250013	-2.615308497	0.310351261	-5.204775048	1.94232E-07	2.75579E-06	Cluster 1
SAOUHSC_T00014		3921333			66.79371769	3.05604585	0.395884291	5.193552504	2.06319E-07	2.91163E-06	Cluster 5
SAOUHSC_T00035		3921161			31.8194526	3.323397922	0.451317604	5.148033011	2.63232E-07	3.69505E-06	Cluster 5
SAOUHSC_02983	YP_501432.1	3921465	gtfB	UDP-N-acetylglucosamine--peptide N-acetylglucosaminyltransferase stabilizing protein GtfB (Glycosyltransferase stabilizing protein GtfB)	402.7251366	-2.401273516	0.272565215	-5.141057768	2.73196E-07	3.81463E-06	Cluster 1
SAOUHSC_01761a	YP_008530242.1	16830702			90.79571801	3.642917041	0.515563692	5.126266808	2.95544E-07	4.10495E-06	#N/A
SAOUHSC_01895	YP_500396.1	3920842		Uncharacterized protein	502.7794547	2.18134947	0.230602797	5.12287572	3.00911E-07	4.15761E-06	Cluster 5
SAOUHSC_01878	YP_500381.1	3921767		Uncharacterized protein	81.38882141	2.335460599	0.263243389	5.073102139	3.91382E-07	5.37947E-06	Cluster 6
SAOUHSC_01008	YP_499558.1	3920269	purE	N5-carboxyaminoimidazole ribonucleotide mutase (N5-CAIR mutase) (EC 5.4.99.18) (5-(carboxyamino)imidazole ribonucleotide mutase)	41.21670331	3.377723124	0.468967626	5.070122095	3.97561E-07	5.43607E-06	Cluster 5
SAOUHSC_02341	YP_500820.1	3920966	atpD	ATP synthase subunit beta (EC 7.1.2.2) (ATP synthase F1 sector subunit beta) (F-ATPase subunit beta)	2685.650776	-2.218275776	0.241116897	-5.052635428	4.35755E-07	5.92762E-06	#N/A
SAOUHSC_02403	YP_500879.1	3919618	mtlD	Mannitol-1-phosphate 5-dehydrogenase (EC 1.1.1.17)	266.1137757	-2.343032026	0.265939761	-5.050136255	4.41495E-07	5.9749E-06	Cluster 1
SAOUHSC_01681	YP_500191.1	3921793	prmA	Ribosomal protein L11 methyltransferase (L11 Mtase) (EC 2.1.1.-)	1475.951007	3.715040502	0.537933816	5.047164578	4.48415E-07	6.03759E-06	#N/A
SAOUHSC_02930	YP_501383.1	3921637		Uncharacterized protein	92.40007936	-3.552090383	0.507617018	-5.027590271	4.96682E-07	6.65352E-06	Cluster 1
SAOUHSC_00884	YP_499437.1	3919231	mnhF1	Na(+)/H(+) antiporter subunit F1 (Mnh complex subunit F1)	255.9597946	-2.411512171	0.281091685	-5.021536558	5.12597E-07	6.83204E-06	Cluster 1
SAOUHSC_01682	YP_500192.1	3921794	dnaJ	Chaperone protein DnaJ	1770.809594	3.925350033	0.586385753	4.988780879	6.07615E-07	8.05777E-06	#N/A
SAOUHSC_01173	YP_499712.1	3920924		Uncharacterized protein	86.11309049	-3.5819521	0.518220976	-4.982338073	6.28206E-07	8.24793E-06	Cluster 1
SAOUHSC_01405	YP_499932.1	3920695		Uncharacterized protein	61.11613997	2.799112033	0.361093128	4.982404516	6.2799E-07	8.24793E-06	Cluster 5
SAOUHSC_00032	YP_498638.1	3919300		Uncharacterized protein	33.6156308	2.864743468	0.377905651	4.93441541	8.03912E-07	1.05026E-05	Cluster 4
SAOUHSC_02547	YP_501010.1	3921136		Molybdenum transport system permease	232.9604388	-2.755237219	0.359292832	-4.885255317	1.03295E-06	1.34283E-05	Cluster 1
SAOUHSC_02546	YP_501009.1	3921135		Molybdenum transport ATP-binding protein ModC, putative	170.0459139	-2.557310867	0.318895849	-4.883446651	1.04247E-06	1.34857E-05	Cluster 1
SAOUHSC_02402	YP_500878.1	3919617		PTS system, mannitol-specific IIa component, putative (EC 2.7.1.69)	157.6249423	-2.704210104	0.350516426	-4.86199784	1.16207E-06	1.49595E-05	Cluster 1

SAOUHSC_01166	YP_499706.1	3920918	pyrB	Aspartate carbamoyltransferase (EC 2.1.3.2) (Aspartate transcarbamylase) (ATCase)	214.403713	-2.78307376	0.367087933	-4.857347795	1.18969E-06	1.52407E-05	Cluster 1
SAOUHSC_02631	YP_501092.1	3921408		Uncharacterized protein	64.64794635	3.017116455	0.419456453	4.808881681	1.51777E-06	1.93497E-05	Cluster 5
SAOUHSC_01387	YP_499914.1	3920678		Phosphate transport system permease protein	11.89851832	4.368552912	0.700843343	4.806427776	1.53651E-06	1.94945E-05	Cluster 5
SAOUHSC_02346	YP_500823.1	3919390	atpH	ATP synthase subunit delta (ATP synthase F(1) sector subunit delta) (F-type ATPase subunit delta) (F-ATPase subunit delta)	829.4273747	-2.013862223	0.211441312	-4.795005346	1.6267E-06	2.05401E-05	Cluster 1
SAOUHSC_00235	YP_498830.1	3919255		Uncharacterized protein	31.88210622	2.738657303	0.363241876	4.786500168	1.69715E-06	2.13275E-05	#N/A
SAOUHSC_00229	YP_498824.1	3920304	scdA	Iron-sulfur cluster repair protein ScdA (Cell wall-related protein ScdA)	83.45118999	-3.341824723	0.490054033	-4.778707175	1.76426E-06	2.20658E-05	Cluster 1
SAOUHSC_02243	YP_500726.1	3919664		Uncharacterized leukocidin-like protein 2	161.0215552	-4.092712045	0.649257834	-4.763457421	1.90304E-06	2.36892E-05	Cluster 1
SAOUHSC_00886	YP_499439.1	3919233	mnhD1	Na(+)/H(+) antiporter subunit D1 (Mnh complex subunit D1)	926.1551008	-2.181360546	0.248641244	-4.751265431	2.02148E-06	2.50454E-05	Cluster 1
SAOUHSC_00999	YP_499551.1	3920399	qoxD	Probable quinol oxidase subunit 4 (EC 1.10.3.-) (Quinol oxidase polypeptide IV)	845.8332576	-2.753680489	0.370415989	-4.734354186	2.19754E-06	2.70995E-05	Cluster 1
SAOUHSC_00674	YP_499233.1	3919437	sarX	HTH-type transcriptional regulator SarX (Staphylococcal accessory regulator X)	114.0004937	-4.054049098	0.645806726	-4.729045048	2.25578E-06	2.76884E-05	#N/A
SAOUHSC_T00061		3921069			29.14236516	4.236513857	0.688245836	4.702554938	2.56926E-06	3.12455E-05	Cluster 5
SAOUHSC_02454	YP_500921.1	3919017	lacB	Galactose-6-phosphate isomerase subunit LacB (EC 5.3.1.26)	14.21593732	3.203039732	0.468397998	4.703350016	2.55927E-06	3.12455E-05	Cluster 4
SAOUHSC_02830	YP_501289.1	3921269		D-lactate dehydrogenase, putative (EC 1.1.1.28)	665.054766	-2.795314966	0.38264243	-4.691886797	2.70697E-06	3.27692E-05	Cluster 1
SAOUHSC_02661	YP_501123.1	3921223		PTS system sucrose-specific IIBC component, putative (EC 2.7.1.69)	209.4228215	1.809922335	0.17268127	4.690273222	2.7284E-06	3.28779E-05	Cluster 3
SAOUHSC_00582	YP_499149.1	3920506		Uncharacterized protein	22.38629512	3.007950244	0.428350837	4.687630023	2.76387E-06	3.31539E-05	Cluster 5
SAOUHSC_01001	YP_499553.1	3920401	qoxB	Probable quinol oxidase subunit 1 (EC 1.10.3.-) (Quinol oxidase polypeptide I)	4950.874274	-2.723343697	0.370663451	-4.649348874	3.32985E-06	3.97623E-05	Cluster 1
SAOUHSC_01974	YP_500471.1	3920450		Uncharacterized protein	1125.038226	-2.643940873	0.354345768	-4.639369281	3.49474E-06	4.15433E-05	#N/A
SAOUHSC_T00040		3921029			92.64794637	2.993281566	0.430365887	4.631597498	3.62855E-06	4.29406E-05	Cluster 5
SAOUHSC_03035	YP_501483.1	3921301		Uncharacterized protein	249.0009223	-2.83559169	0.397253526	-4.620705848	3.82437E-06	4.50558E-05	Cluster 1
SAOUHSC_02541	YP_501004.1	3921130		Molybdopterin-guanine dinucleotide biosynthesis protein MobB	141.2266976	-2.506820402	0.328470319	-4.587386796	4.48829E-06	5.26426E-05	Cluster 1
SAOUHSC_02909	YP_501362.1	3921361		Dihydroorotate dehydrogenase (quinone) (EC 1.3.5.2)	123.9518811	-2.624403282	0.355750532	-4.5661303	4.9681E-06	5.80125E-05	Cluster 1
SAOUHSC_02768	YP_501227.1	3921423		Uncharacterized protein	20.49829685	3.607486445	0.578280721	4.509032292	6.5124E-06	7.57102E-05	Cluster 5
SAOUHSC_00663	YP_499222.1	3919953		Uncharacterized protein	179.6169253	2.243720527	0.275949016	4.507066367	6.57301E-06	7.60797E-05	#N/A

SAOUHSC_02343	YP_500821.2	3920967	atpG	ATP synthase gamma chain (ATP synthase F1 sector gamma subunit) (F-ATPase gamma subunit)	1369.384702	-2.277444488	0.284739878	-4.48635609	7.24516E-06	8.34934E-05	#N/A
SAOUHSC_00560	YP_499132.1	3920605		Uncharacterized protein	202.0176328	3.611533532	0.584873098	4.465128486	8.00207E-06	9.18151E-05	#N/A
SAOUHSC_02922	YP_501374.1	3921373	ldh2	L-lactate dehydrogenase 2 (L-LDH 2) (EC 1.1.1.27)	389.772626	-3.279968027	0.51097354	-4.462008007	8.11952E-06	9.27594E-05	Cluster 2
SAOUHSC_00233	YP_498828.1	3920308	lrgB	Antiholin-like protein LrgB	177.2970696	-3.401592963	0.538582922	-4.459096019	8.2306E-06	9.36231E-05	Cluster 2
SAOUHSC_01401	YP_499928.1	3920691	lysA	Diaminopimelate decarboxylase (DAP decarboxylase) (DAPDC) (EC 4.1.1.20)	665.1050606	2.773642199	0.398241401	4.45368612	8.44085E-06	9.56026E-05	#N/A
SAOUHSC_02610	YP_501071.1	3921388	hutG	Formimidoylglutamase (EC 3.5.3.8) (Formiminoglutamase) (Formiminoglutamate hydrolase)	499.0092962	-2.403858879	0.31592438	-4.443654775	8.84435E-06	9.97446E-05	#N/A
SAOUHSC_02347	YP_500824.1	3919391	atpF	ATP synthase subunit b (ATP synthase F(0) sector subunit b) (ATPase subunit I) (F-type ATPase subunit b) (F-ATPase subunit b)	937.9553611	-1.824835013	0.185681866	-4.442194761	8.90459E-06	9.99967E-05	Cluster 1
SAOUHSC_03008	YP_501457.1	3921489		imidazole glycerol phosphate synthase subunit hisFutative	125.9605007	2.20933041	0.275851079	4.383997387	1.16521E-05	0.000130296	#N/A
SAOUHSC_02289	YP_500771.1	3919164	ilvA	L-threonine dehydratase biosynthetic IlvA (EC 4.3.1.19) (Threonine deaminase)	346.9752442	2.802045208	0.411411951	4.380147941	1.18599E-05	0.00013206	#N/A
SAOUHSC_02872	YP_501327.1	3921543		Uncharacterized protein	310.2962172	3.526905249	0.579785268	4.358346772	1.31049E-05	0.00014531	Cluster 4
SAOUHSC_02785	YP_501245.1	3921440		Uncharacterized protein	79.11950817	-3.242609501	0.514728875	-4.356875256	1.31932E-05	0.000145678	Cluster 1
SAOUHSC_02135	YP_500625.1	3921832		Uncharacterized protein	135.8130508	-2.723321709	0.396239366	-4.349193587	1.36639E-05	0.000150246	Cluster 1
SAOUHSC_01991	YP_500488.1	3921874		ABC transporter, permease protein, putative	151.3246684	2.539167601	0.35480352	4.338084357	1.4373E-05	0.000157387	#N/A
SAOUHSC_00888	YP_499441.1	3921734	mnhB1	Na(+)/H(+) antiporter subunit B1 (Mnh complex subunit B1)	359.0910078	-1.987062002	0.228211728	-4.325202778	1.52392E-05	0.000166182	Cluster 1
SAOUHSC_01680	YP_500190.1	3921792		Ribosomal RNA small subunit methyltransferase E (EC 2.1.1.193)	355.2488498	2.716066583	0.401262411	4.276669176	1.8971E-05	0.000206027	#N/A
SAOUHSC_01000	YP_499552.1	3920400	qoxC	Probable quinol oxidase subunit 3 (EC 1.10.3.-) (Quinol oxidase polypeptide III)	2134.192898	-2.723488532	0.404009569	-4.265959684	1.99045E-05	0.000215278	Cluster 1
SAOUHSC_02350	YP_500826.1	3919393	atpB	ATP synthase subunit a (ATP synthase F0 sector subunit a) (F-ATPase subunit 6)	1232.133976	-1.828049254	0.194305016	-4.261594849	2.02973E-05	0.000218631	Cluster 1
SAOUHSC_03012	YP_501461.1	3921493		Histidinol-phosphate aminotransferase, putative (EC 2.6.1.9)	75.61769661	2.893633148	0.444799089	4.257277484	2.06931E-05	0.00022109	#N/A
SAOUHSC_03046	YP_501492.1	3921310		Helix-turn-helix domain protein	48.04546643	-4.121099881	0.733099244	-4.257404311	2.06814E-05	0.00022109	Cluster 1
SAOUHSC_T00036		3921162			59.41984388	2.563790106	0.367616249	4.253865571	2.10111E-05	0.000223582	Cluster 5
SAOUHSC_00075	YP_498675.1	3919453	sbnA	N-(2-amino-2-carboxyethyl)-L-glutamate synthase (ACEGA synthase) (EC 2.5.1.140) (Staphyloferrin B biosynthesis protein SbnA)	10.83603717	4.053301439	0.72176904	4.230302592	2.33377E-05	0.000247342	Cluster 6
SAOUHSC_01604	YP_500119.1	3920020		Uncharacterized protein	162.4692659	2.465787721	0.348134729	4.21040362	2.54915E-05	0.000269088	#N/A
SAOUHSC_02450	YP_500917.1	3919013		Pts system, lactose-specific IIBC component, putative	54.57906502	2.470056757	0.350381284	4.19559156	2.7216E-05	0.000286148	#N/A
SAOUHSC_00885	YP_499438.1	3919232	mnhE1	Na(+)/H(+) antiporter subunit E1 (Mnh complex subunit E1)	399.9646394	-1.966869988	0.230934701	-4.186767881	2.82955E-05	0.000296317	Cluster 1

SAOUHSC_03011	YP_501460.1	3921492	hisB	Imidazoleglycerol-phosphate dehydratase (IGPD) (EC 4.2.1.19)	47.81266568	2.554227202	0.371833776	4.179897849	2.9164E-05	0.000304205	#N/A
SAOUHSC_01364	YP_499891.1	3920069	tyrA	Prephenate dehydrogenase (PDH) (EC 1.3.1.12)	293.8611357	1.692738139	0.165803216	4.178074198	2.93988E-05	0.000305446	Cluster 3
SAOUHSC_01634	YP_500147.1	3919971	gcvT	Aminomethyltransferase (EC 2.1.2.10) (Glycine cleavage system T protein)	165.9731676	2.591225471	0.383304835	4.151331595	3.30546E-05	0.000342083	Cluster 6
SAOUHSC_00262	YP_498856.1	3919204	essC	ESAT-6 secretion machinery protein EssC	82.01041201	-3.345147456	0.566122244	-4.142475379	3.43577E-05	0.00035418	#N/A
SAOUHSC_00887	YP_499440.1	3919234	mnhC1	Na(+)/H(+) antiporter subunit C1 (Mnh complex subunit C1)	327.4822028	-2.085643033	0.262310508	-4.138770654	3.49172E-05	0.000358411	Cluster 1
SAOUHSC_01504	YP_500022.1	3919047		Ferredoxin, putative	163.4734181	3.374818674	0.573909598	4.137966475	3.50398E-05	0.000358411	Cluster 5
SAOUHSC_01012	YP_499562.1	3920273	purQ	Phosphoribosylformylglycinamidine synthase subunit PurQ (FGAM synthase) (EC 6.3.5.3) (Formylglycinamide ribonucleotide amidotransferase subunit I) (FGAR amidotransferase I) (FGAR-AT I) (Glutaminase PurQ) (EC 3.5.1.2) (Phosphoribosylformylglycinamidine synthase subunit I)	46.49895118	2.271101069	0.308414845	4.121400406	3.76576E-05	0.000383701	Cluster 5
SAOUHSC_01264	YP_499797.1	3919917		Uncharacterized protein	21.20716259	2.898522026	0.461240924	4.116117903	3.85307E-05	0.000388847	#N/A
SAOUHSC_01646	YP_500158.1	3920098		Glucokinase, putative	426.3916147	-1.874479077	0.212441134	-4.116335949	3.84943E-05	0.000388847	Cluster 1
SAOUHSC_02828	YP_501287.1	3921267		Uncharacterized protein	90.8723175	2.19821636	0.291134808	4.115675376	3.86047E-05	0.000388847	Cluster 3
SAOUHSC_02455	YP_500922.1	3919018	lacA	Galactose-6-phosphate isomerase subunit LacA (EC 5.3.1.26)	13.53199368	3.512580792	0.611134723	4.111336986	3.93375E-05	0.000394721	#N/A
SAOUHSC_01084	YP_499629.1	3919245		Uncharacterized protein	51.03168876	3.379696619	0.580715244	4.097871796	4.16966E-05	0.000416808	Cluster 5
SAOUHSC_01794	YP_500298.1	3920435		Glyceraldehyde-3-phosphate dehydrogenase (EC 1.2.1.-)	694.4026241	2.342588109	0.328541986	4.0865039	4.37922E-05	0.000436104	#N/A
SAOUHSC_T0003		3921322			16.46648325	4.142042015	0.773464559	4.062296041	4.85924E-05	0.000482088	#N/A
SAOUHSC_03047	YP_501493.1	3921311		Uncharacterized protein	33.6068488	-4.327068414	0.820474063	-4.055056172	5.01223E-05	0.000495403	Cluster 1
SAOUHSC_00798	YP_499354.1	3919361	gpmI	2,3-bisphosphoglycerate-independent phosphoglycerate mutase (BPG-independent PGAM) (Phosphoglyceromutase) (iPGM) (EC 5.4.2.12)	1917.869573	-2.052801294	0.260735363	-4.037815508	5.39513E-05	0.000531259	Cluster 1
SAOUHSC_00715	YP_499274.1	3919342	saeR	Response regulator SaeR (Staphylococcus exoprotein expression protein R)	496.9399228	-2.045664517	0.259170536	-4.034658154	5.46818E-05	0.000536451	#N/A
SAOUHSC_00540	YP_499112.1	3920820		Uncharacterized protein	34.7523545	2.72753019	0.430456085	4.013255364	5.98871E-05	0.000583181	Cluster 5
SAOUHSC_02972	YP_501423.1	3921673	isaB	Immunodominant staphylococcal antigen B	245.7555943	-2.717637109	0.427961419	-4.013532608	5.98167E-05	0.000583181	Cluster 1
SAOUHSC_01385	YP_499912.1	3920794	pstB	Phosphate import ATP-binding protein PstB (EC 7.3.2.1) (ABC phosphate transporter) (Phosphate-transporting ATPase)	15.35395	3.296862081	0.573306601	4.006341594	6.16664E-05	0.000598301	#N/A
SAOUHSC_01850	YP_500354.1	3920528		Catabolite control protein A	714.3418824	1.867728617	0.217188442	3.995279901	6.46179E-05	0.000624639	#N/A
SAOUHSC_00232	YP_498827.1	3920307	lrgA	Antiholin-like protein LrgA	62.81032707	-3.844083621	0.713074481	-3.988480441	6.64979E-05	0.000640467	#N/A
SAOUHSC_02171	YP_500658.1	3921865		Staphylokinase, putative	88.53020116	-3.63390298	0.661636859	-3.980889131	6.8658E-05	0.00065737	Cluster 1

SAOUHSC_02882	YP_501336.1	3921552	crtO	Glycosyl-4,4'-diaponeurosporenoate acyltransferase (EC 2.3.1.-)	64.14125667	-3.456857084	0.617212838	-3.980567047	6.87511E-05	0.00065737	#N/A
SAOUHSC_00328	YP_498917.1	3919593	tatC	Sec-independent protein translocase protein TatC	80.76952715	2.508284674	0.379728926	3.972003636	7.12706E-05	0.000679001	Cluster 6
SAOUHSC_00284	YP_498875.1	3918967		5'-nucleotidase, lipoprotein e(P4) family	44.4778808	-4.525061153	0.889760368	-3.961809586	7.43839E-05	0.000706111	#N/A
SAOUHSC_00049	YP_498654.1	3919081		Uncharacterized protein	121.6005623	-3.228096986	0.563300113	-3.955435007	7.63955E-05	0.000722608	#N/A
SAOUHSC_01336	YP_499865.1	3920200		UPF0291 protein SAOUHSC_01336	81.18322135	2.374445388	0.348285571	3.946317338	7.93624E-05	0.000747991	Cluster 4
SAOUHSC_01328	YP_499858.1	3920193	rpmG1	50S ribosomal protein L33 1	320.5567502	2.149354297	0.291527773	3.942520762	8.06297E-05	0.00075723	#N/A
SAOUHSC_00639	YP_499199.1	3920047		Uncharacterized protein	116.9592526	2.139977521	0.289437234	3.938600114	8.19584E-05	0.000766979	#N/A
SAOUHSC_01129	YP_499672.1	3920729	arcC1	Carbamate kinase 1 (EC 2.7.2.2)	63.031678	-3.888321857	0.734155084	-3.934212158	8.347E-05	0.000778365	Cluster 1
SAOUHSC_T00021		3921340			45.62898577	2.587933288	0.403972012	3.930800254	8.46636E-05	0.000781214	Cluster 6
SAOUHSC_00910	YP_499463.1	3920797		Uncharacterized protein	225.7430491	2.02719521	0.261302717	3.931054458	8.45741E-05	0.000781214	Cluster 5
SAOUHSC_01902	YP_500403.1	3920849		Uncharacterized protein	71.18165854	2.700763868	0.432547862	3.931966879	8.42537E-05	0.000781214	Cluster 6
SAOUHSC_01592	YP_500107.1	3920008		Transcriptional regulator, Fur, putative	319.1787804	2.32483898	0.337565422	3.924688054	8.68422E-05	0.000798525	#N/A
SAOUHSC_T00016		3921335			123.020012	2.501754565	0.384749231	3.903203552	9.49278E-05	0.000869842	Cluster 5
SAOUHSC_02908	YP_501361.1	3921360		Uncharacterized protein	83.05885229	-3.425664909	0.622979139	-3.893653508	9.87457E-05	0.000901695	#N/A
SAOUHSC_01002	YP_499554.1	3920402	qoxA	Probable quinol oxidase subunit 2 (EC 1.10.3.-) (Quinol oxidase polypeptide II)	2398.561993	-2.410381586	0.364157831	-3.872995348	0.000107506	0.000974942	Cluster 1
SAOUHSC_01386	YP_499913.1	3920677		Phosphate transport system permease protein PstA	16.8468517	3.575134912	0.664803888	3.873525648	0.000107272	0.000974942	#N/A
SAOUHSC_00559	YP_499131.1	3920604		Protein VraC	62.45920814	3.747100884	0.711472211	3.861149938	0.000112855	0.001019943	Cluster 5
SAOUHSC_01384	YP_499911.1	3920793		Phosphate-specific transport system accessory protein PhoU	18.58539782	3.327509884	0.606390017	3.838305082	0.000123886	0.001115824	#N/A
SAOUHSC_01334	YP_499864.1	3920199		Uncharacterized protein	81.63985872	3.029235071	0.531886567	3.815165108	0.000136092	0.001221587	#N/A
SAOUHSC_02544	YP_501007.1	3921133		Molybdenum cofactor biosynthesis protein B	92.24766525	-2.589073221	0.417224473	-3.808676919	0.000139712	0.001249834	Cluster 1
SAOUHSC_00201	YP_498798.1	3920357		conserved hypothetical protein	55.83211259	-4.168543272	0.832201737	-3.807422084	0.000140423	0.001251947	Cluster 1
SAOUHSC_00162		3919869		Type I restriction enzyme R Protein (EC 3.1.21.3)	259.2563319	-2.475669864	0.39059901	-3.77796622	0.000158114	0.001404928	#N/A
SAOUHSC_00833	YP_499387.1	3918945		Uncharacterized protein	201.4713969	2.359516435	0.360823865	3.767811857	0.000164685	0.001458399	Cluster 4
SAOUHSC_01205	YP_499742.1	3919471	ftsY	Signal recognition particle receptor FtsY (SRP receptor)	199.5591533	-2.29154004	0.345343154	-3.739874454	0.000184112	0.00162499	Cluster 1
SAOUHSC_01003	YP_499555.1	3920403		Uncharacterized protein	8.627678046	4.12272283	0.836383469	3.733601804	0.000188761	0.001660466	Cluster 5
SAOUHSC_02744	YP_501205.1	3921618		Amino acid ABC transporter, ATP-binding protein, putative	106.0850416	-2.560304628	0.418336403	-3.729784495	0.000191644	0.001680224	Cluster 1
SAOUHSC_01644	YP_500156.1	3920096		Uncharacterized protein	261.368107	-1.836436587	0.224777921	-3.721168796	0.000198303	0.001732852	#N/A
SAOUHSC_02656	YP_501118.1	3921218		Uncharacterized protein	94.659781	-2.631553568	0.439227741	-3.714595907	0.000203529	0.001772647	Cluster 1
SAOUHSC_00603	YP_499166.1	3918922		Uncharacterized protein	132.9141037	-2.217594687	0.328632489	-3.705034428	0.000211362	0.001834817	Cluster 1

SAOUHSC_01670	YP_500181.1	3920082		Cytidine deaminase (EC 3.5.4.5) (Cytidine aminohydrolase)	146.2680721	-2.081362602	0.292188213	-3.700911105	0.000214827	0.001858556	Cluster 1
SAOUHSC_02849	YP_501306.1	3921522		Pyruvate oxidase, putative (EC 1.2.3.3)	955.81855	-1.795748221	0.215060637	-3.70011096	0.000215505	0.001858556	#N/A
SAOUHSC_00883	YP_499436.1	3919230	mnhG1	Na(+)/H(+) antiporter subunit G1 (Mnh complex subunit G1)	247.0120807	-2.169083684	0.316897777	-3.689150783	0.000225004	0.001934154	Cluster 1
SAOUHSC_T00017		3921336			25.90670018	3.645159646	0.718551012	3.681241279	0.000232101	0.001988686	Cluster 5
SAOUHSC_T00060		3921068			14.7187466	3.442622392	0.667786342	3.657790282	0.000254399	0.002172683	Cluster 5
SAOUHSC_01082	YP_499628.1	3919244		conserved hypothetical protein	36.09480249	3.197798262	0.60235786	3.648658727	0.000263613	0.002244112	Cluster 5
SAOUHSC_01233	YP_499766.1	3920258		Uncharacterized protein	275.8198608	2.912441694	0.524840378	3.643853967	0.000268586	0.002279093	Cluster 5
SAOUHSC_01650	YP_500162.1	3920102		5-formyltetrahydrofolate cyclo-ligase (EC 6.3.3.2)	102.2418567	-2.476261275	0.405841688	-3.637529899	0.000275265	0.002328285	Cluster 1
SAOUHSC_02851	YP_501308.1	3921524	cidA	Holin-like protein CidA	39.11957131	-4.194063395	0.878612779	-3.63534821	0.000277605	0.002340577	Cluster 1
SAOUHSC_01821	YP_500326.1	3919291		Uncharacterized protein	193.2919047	2.014009077	0.280381363	3.616535234	0.000298573	0.002509342	#N/A
SAOUHSC_01843	YP_500348.1	3920522		conserved hypothetical protein	56.49360756	2.068396779	0.296131068	3.607851033	0.000308744	0.002586586	#N/A
SAOUHSC_02464	YP_500932.1	3919027		Uncharacterized protein	48.09426461	-3.233480588	0.619627228	-3.604555264	0.000312688	0.00261134	Cluster 1
SAOUHSC_T0004		3921323			29.02165913	3.027259588	0.562826454	3.601926618	0.000315868	0.002629572	Cluster 5
SAOUHSC_T00050		3921039			7.528816485	4.580367312	0.994818504	3.599015597	0.000319424	0.002650818	Cluster 6
SAOUHSC_00555	YP_499127.1	3920600		Hydrolase, haloacid dehalogenase-like	41.22970823	2.311435375	0.365096777	3.592021232	0.000328123	0.002714473	Cluster 6
SAOUHSC_00146	YP_498745.1	3919854		Uncharacterized protein	89.34202269	-2.851576907	0.517183168	-3.580118268	0.000343439	0.002832296	Cluster 1
SAOUHSC_00545	YP_499118.1	3920825	sdrD	Serine-aspartate repeat-containing protein D	601.2367576	-2.342464144	0.375164093	-3.578338572	0.000345785	0.002842765	#N/A
SAOUHSC_00185	YP_498782.1	3919499		Uncharacterized sensor-like histidine kinase SAOUHSC_00185 (EC 2.7.13.3)	52.45810444	-3.693728648	0.753712544	-3.57394695	0.00035164	0.002881921	#N/A
SAOUHSC_01363	YP_499890.1	3920068		Uncharacterized protein	385.186297	2.142069494	0.32000966	3.568859427	0.000358539	0.002929361	Cluster 3
SAOUHSC_T00020		3921339			18.15214539	4.569140079	1.006104728	3.547483657	0.00038893	0.003167858	#N/A
SAOUHSC_02287	YP_500769.1	3919162	leuC	3-isopropylmalate dehydratase large subunit (EC 4.2.1.33) (Alpha-IPM isomerase) (IPMI) (Isopropylmalate isomerase)	443.5088941	2.309716368	0.370044413	3.539349118	0.000401115	0.003257054	#N/A
SAOUHSC_02412	YP_500887.1	3919626		Uncharacterized protein	34.31864721	3.473717133	0.701903314	3.524298979	0.000424605	0.003437215	#N/A
SAOUHSC_02281	YP_500763.1	3919156	ilvD	Dihydroxy-acid dehydratase (DAD) (EC 4.2.1.9)	514.4671418	2.39368242	0.395924614	3.520070162	0.000431433	0.003481807	Cluster 5
SAOUHSC_00991	YP_499543.1	3920391		Uncharacterized protein	50.08891155	2.719711731	0.48965885	3.512060959	0.000444646	0.003577502	#N/A
SAOUHSC_02225	YP_500710.1	3919648		Conserved hypothetical phage protein	27.05240785	3.868499872	0.82117897	3.493148237	0.000477361	0.003829048	#N/A
SAOUHSC_00287	YP_498878.1	3918969		ABC transporter, ATP-binding protein, putative	185.4433664	-1.949084944	0.272187809	-3.486875291	0.000488699	0.003908113	#N/A
SAOUHSC_01222	YP_499758.1	3920250	topA	DNA topoisomerase 1 (EC 5.6.2.2) (DNA topoisomerase I) (Omega-protein) (Relaxing enzyme) (Swivelase) (Untwisting enzyme)	610.9216412	2.031432572	0.295877401	3.486013361	0.000490277	0.003908881	Cluster 5

SAOUHSC_02284	YP_500766.1	3919159	ilvC	Ketol-acid reductoisomerase (NADP(+)) (KARI) (EC 1.1.1.86) (Acetohydroxy-acid isomeroreductase) (AHIR) (Alpha-keto-beta-hydroxylacyl reductoisomerase) (Ketol-acid reductoisomerase type 1) (Ketol-acid reductoisomerase type I)	506.6350272	2.469032561	0.422355459	3.478190061	0.000504812	0.004012646	#N/A
SAOUHSC_02340	YP_500819.1	3920965	atpC	ATP synthase epsilon chain (ATP synthase F1 sector epsilon subunit) (F-ATPase epsilon subunit)	689.2183746	-2.186725967	0.342468527	-3.465211762	0.000529814	0.004198737	#N/A
SAOUHSC_00310	YP_498900.1	3919537	ulaA	Uncharacterized protein	216.9817801	-3.10660019	0.609653287	-3.455406927	0.000549463	0.004341415	Cluster 2
SAOUHSC_00069	YP_498670.1	3919448	spa	Immunoglobulin G-binding protein A (IgG-binding protein A) (Staphylococcal protein A) (SpA)	2604.360271	-3.15012281	0.622904555	-3.451769285	0.000556924	0.004387229	#N/A
SAOUHSC_02349	YP_500825.1	3919392	atpE	ATP synthase subunit c (ATP synthase F(0) sector subunit c) (F-type ATPase subunit c) (F-ATPase subunit c) (Lipid-binding protein)	632.8088621	-1.850817051	0.247720166	-3.434589379	0.000593452	0.004661074	Cluster 1
SAOUHSC_02862	YP_501318.1	3921534	clpL	ATP-dependent Clp protease ATP-binding subunit ClpL	214.4112904	-2.692458906	0.496405578	-3.409427659	0.000650993	0.005097839	#N/A
SAOUHSC_02662	YP_501124.1	3921224		PTS system sucrose-specific IIBC component (EC 2.7.1.69)	62.40374969	1.867465911	0.254520885	3.408230766	0.000653856	0.005105104	Cluster 3
SAOUHSC_01706	YP_500216.1	3921096		Uncharacterized protein	63.17619967	2.111912472	0.327114755	3.399151077	0.000675954	0.00526207	#N/A
SAOUHSC_01365	YP_499892.1	3920070		Uncharacterized protein	305.5337718	1.565331726	0.166540659	3.394556802	0.000687398	0.00533542	Cluster 3
SAOUHSC_02086	YP_500578.1	3919772		PV83 orf 4-like protein-related protein	4.219616718	3.753695307	0.819427994	3.360509194	0.000777989	0.006020862	#N/A
SAOUHSC_01787	YP_500292.1	3920429		Uncharacterized protein	758.7512529	-1.779312187	0.232101558	-3.357634458	0.000786125	0.006066035	#N/A
SAOUHSC_00581	YP_499148.1	3920505		Uncharacterized protein	88.45484914	2.23536283	0.368550967	3.351945701	0.000802458	0.00617401	#N/A
SAOUHSC_02584	YP_501046.1	3921580		Uncharacterized protein	115.7779406	-2.1259576	0.336145572	-3.349613063	0.000809245	0.006208134	Cluster 1
SAOUHSC_02101	YP_500592.1	3921173		Uncharacterized protein	91.48612262	2.030388282	0.308046252	3.344914196	0.00082308	0.006295968	#N/A
SAOUHSC_01997	YP_500494.1	3921877	perR	Peroxide-responsive repressor PerR	1514.389332	1.591628487	0.177540787	3.332352505	0.000861151	0.006568143	Cluster 3
SAOUHSC_01204	YP_499741.1	3919470	smc	Chromosome partition protein Smc	294.6090258	-2.138150576	0.341952487	-3.328388067	0.000873501	0.006643139	#N/A
SAOUHSC_02769	YP_501228.1	3921424		Uncharacterized protein	15.89638662	3.321441574	0.698898224	3.321573149	0.000895115	0.006787957	Cluster 5
SAOUHSC_00769	YP_499326.1	3919060	secA1	Protein translocase subunit SecA 1	1665.171631	-1.470091727	0.141629404	-3.319167581	0.000902862	0.006827088	Cluster 1
SAOUHSC_01668	YP_500179.2	3920080	era	GTPase Era	260.2634527	-1.943876455	0.285869553	-3.301773288	0.000960757	0.007244107	Cluster 1
SAOUHSC_00797	YP_499353.1	3919360	tpiA	Triosephosphate isomerase (TIM) (TPI) (EC 5.3.1.1) (Triose-phosphate isomerase)	1088.824728	-2.153699959	0.351655698	-3.280765718	0.001035257	0.007783599	#N/A
SAOUHSC_02549	YP_501011.1	3921137		Molybdenum ABC transporter, periplasmic molybdate-binding protein	213.750341	-2.432571722	0.437233741	-3.27644367	0.001051233	0.00788126	Cluster 1
SAOUHSC_02330	YP_500809.1	3920955		Phosphomethylpyrimidine kinase (EC 2.7.4.7)	20.71297614	-3.987619546	0.912413033	-3.274415684	0.001058807	0.007915559	#N/A
SAOUHSC_00329	YP_498918.1	3919594	tatA	Sec-independent protein translocase protein TatA	56.98184333	2.283103484	0.392030405	3.272969308	0.00106424	0.007933701	Cluster 6
SAOUHSC_02329	YP_500808.1	3920954	thiM	Hydroxyethylthiazole kinase (EC 2.7.1.50) (4-methyl-5-beta-hydroxyethylthiazole kinase) (TH kinase) (Thz kinase)	36.33771026	-3.582068463	0.789717016	-3.269612294	0.00107695	0.008005833	Cluster 1
SAOUHSC_02288	YP_500770.1	3919163	leuD	3-isopropylmalate dehydratase small subunit (EC 4.2.1.33) (Alpha-IPM isomerase) (IPMI) (Isopropylmalate isomerase)	245.4245348	2.482381953	0.454852127	3.259041487	0.001117893	0.008286854	#N/A
SAOUHSC_00258	YP_498852.1	3919200	esaA	ESAT-6 secretion accessory factor EsaA	134.5204961	-2.431116435	0.439687169	-3.254851482	0.001134517	0.008386526	#N/A

SAOUHSC_02087	YP_500579.1	3919773		Conserved hypothetical phage protein	4.240154825	3.637966874	0.813894191	3.241166853	0.001190415	0.008775153	#N/A
SAOUHSC_02285	YP_500767.1	3919160	leuA	2-isopropylmalate synthase (EC 2.3.3.13) (Alpha-IPM synthase) (Alpha-isopropylmalate synthase)	605.6815673	2.377162223	0.425391959	3.237395991	0.001206259	0.00886718	#N/A
SAOUHSC_01193	YP_499732.1	3919326		Uncharacterized protein SAOUHSC_01193	488.1639305	1.872752466	0.269922137	3.233348985	0.00122348	0.008968788	Cluster 5
SAOUHSC_02224	YP_500709.1	3919647	SAOUHSC_02076	Phi PVL orf 38-like protein-related protein	8.71274658	4.024946142	0.937811931	3.225535997	0.00125737	0.00919169	#N/A
SAOUHSC_00408	YP_498994.1	3920566		Uncharacterized protein	30.38554756	-3.56849077	0.797855923	-3.219241341	0.001285303	0.009369927	#N/A
SAOUHSC_02801	YP_501260.1	3921455	gtaB	UTP--glucose-1-phosphate uridylyltransferase (EC 2.7.7.9) (Alpha-D-glucosyl-1-phosphate uridylyltransferase) (UDP-glucose pyrophosphorylase) (UDPGP) (Uridine diphosphoglucose pyrophosphorylase)	78.76988147	-3.191199644	0.685820314	-3.195005455	0.001398282	0.010165471	#N/A
SAOUHSC_00209	YP_498805.1	3920364		PTS system, glucose-specific IIBC component, putative (EC 2.7.1.69)	114.7495766	-2.395100399	0.437802869	-3.186594926	0.001439582	0.010436972	Cluster 1
SAOUHSC_00051	YP_498656.1	3919083	plc	1-phosphatidylinositol phosphodiesterase (EC 4.6.1.13) (Phosphatidylinositol diacylglycerol-lyase) (Phosphatidylinositol-specific phospholipase C) (PI-PLC)	23.11514003	-3.616038648	0.822171833	-3.181863626	0.001463307	0.010552268	Cluster 1
SAOUHSC_00697	YP_499256.1	3921001		Uncharacterized protein	68.46306189	-2.764018917	0.554404075	-3.181828918	0.001463482	0.010552268	#N/A
SAOUHSC_00061	YP_498665.1	3919092		Uncharacterized protein	69.9199156	-2.44601191	0.457894853	-3.157956245	0.001588794	0.011424598	#N/A
SAOUHSC_02737	YP_501198.1	3921611		Epimerase/dehydratase, putative	102.4015204	1.806915502	0.257006017	3.139675532	0.001691351	0.012129006	Cluster 5
SAOUHSC_01013	YP_499563.1	3920274	purL	Phosphoribosylformylglycinamidine synthase subunit PurL (FGAM synthase) (EC 6.3.5.3) (Formylglycinamide ribonucleotide amidotransferase subunit II) (FGAR amidotransferase II) (FGAR-AT II) (Glutamine amidotransferase PurL) (Phosphoribosylformylglycinamidine synthase subunit II)	148.2963903	1.81898256	0.261193552	3.135538971	0.001715386	0.012268034	Cluster 6
SAOUHSC_00905	YP_499458.1	3921751	addA	ATP-dependent helicase/nuclease subunit A (EC 3.1.-.-) (EC 3.6.4.12) (ATP-dependent helicase/nuclease AddA)	567.3457555	-1.523187822	0.167128523	-3.130452015	0.001745375	0.01244877	Cluster 1
SAOUHSC_00203	YP_498800.1	3920359		Uncharacterized protein	44.86127364	-2.725779643	0.556871317	-3.099063628	0.001941333	0.013809106	Cluster 1
SAOUHSC_T00023		3921149			40.00213047	2.866868297	0.603064087	3.095638319	0.001963898	0.013923482	Cluster 5
SAOUHSC_T00058		3921047			18.5150061	2.940366038	0.626930699	3.095024761	0.001967965	0.013923482	Cluster 5
SAOUHSC_01784	YP_500289.1	3920426	rplT	50S ribosomal protein L20	349.1946733	2.752594167	0.567009145	3.090945152	0.001995205	0.014078464	Cluster 5
SAOUHSC_00901	YP_499454.1	3921747		Uncharacterized protein	188.873533	1.822252579	0.266453248	3.085916894	0.002029255	0.014280546	#N/A
SAOUHSC_01647	YP_500159.1	3920099		Uncharacterized protein	127.935754	-1.978136378	0.317257341	-3.083100851	0.002048557	0.014378038	Cluster 1
SAOUHSC_00313	YP_498903.1	3919540		Uncharacterized protein	240.6852603	-2.8494669	0.600287188	-3.080970135	0.002063273	0.014442914	Cluster 2
SAOUHSC_T00056		3921045			139.2941872	2.198038219	0.38908289	3.079133652	0.002076035	0.014493802	#N/A
SAOUHSC_01590	YP_500105.1	3920006		Uncharacterized protein	28.36659559	2.707411254	0.556185079	3.069861668	0.002141579	0.014911947	Cluster 5
SAOUHSC_01005	YP_499556.1	3920404		Uncharacterized protein	383.7309431	1.988541483	0.322715303	3.063199898	0.002189837	0.015207843	Cluster 3
SAOUHSC_00544	YP_499117.1	3920824	sdrC	Serine-aspartate repeat-containing protein C	358.6411174	-2.23151853	0.402475682	-3.059858234	0.002214418	0.015338185	#N/A
SAOUHSC_01836	YP_500342.1	3921786		Uncharacterized protein	117.8250539	-2.023549329	0.335091761	-3.054534455	0.002254101	0.015572181	#N/A

SAOUHSC_00208	YP_498804.1	3920363		Uncharacterized protein	14.25262661	-3.712750157	0.888584326	-3.052889948	0.00226649	0.015616888	Cluster 1
SAOUHSC_02784	YP_501244.1	3921439		Uncharacterized protein	68.24512264	-2.363225988	0.446743073	-3.0514765	0.002277189	0.015649742	Cluster 1
SAOUHSC_02387	YP_500864.1	3919604		Uncharacterized protein	136.7091506	-2.490332966	0.489033532	-3.047506703	0.002307484	0.015816753	#N/A
SAOUHSC_00881	YP_499434.1	3919228		Uncharacterized protein	170.6608336	1.618893173	0.203184167	3.045971453	0.002319299	0.015856551	Cluster 3
SAOUHSC_01785	YP_500290.1	3920427	rpmI	50S ribosomal protein L35	429.211427	2.756331409	0.577217514	3.042754881	0.002344232	0.015985603	Cluster 5
SAOUHSC_02351	YP_500827.1	3919394		Uncharacterized protein	753.3142865	-1.634017459	0.210211145	-3.016098217	0.002560503	0.01741538	Cluster 1
SAOUHSC_02286	YP_500768.1	3919161	leuB	3-isopropylmalate dehydrogenase (EC 1.1.1.85) (3-IPM-DH) (Beta-IPM dehydrogenase) (IMDH)	333.088165	2.106575783	0.368367347	3.004000737	0.002664547	0.01807645	#N/A
SAOUHSC_T00028		3921154			8.975207792	3.897308426	0.970122417	2.986538993	0.002821549	0.019092478	#N/A
SAOUHSC_00184	YP_498781.1	3919498		Uncharacterized response regulatory protein SAOUHSC_00184	39.17955504	-3.090303731	0.701154772	-2.98123013	0.00287093	0.019376939	#N/A
SAOUHSC_02110	YP_500602.1	3921182		Uncharacterized protein	100.5294391	2.050694617	0.352574688	2.980062534	0.002881895	0.019401332	#N/A
SAOUHSC_02945	YP_501397.1	3921651		Siroheme synthase, putative	58.73285211	-2.650466734	0.554679961	-2.975529768	0.00292483	0.019640268	#N/A
SAOUHSC_01093	YP_499638.1	3920736	pheT	Phenylalanine--tRNA ligase beta subunit (EC 6.1.1.20) (Phenylalanyl-tRNA synthetase beta subunit) (PheRS)	316.7150088	-1.785868113	0.266157368	-2.952644591	0.003150645	0.021102923	#N/A
SAOUHSC_01603	YP_500118.1	3920019		Uncharacterized protein	109.2368298	2.135495981	0.385249544	2.947429786	0.003204275	0.021407803	#N/A
SAOUHSC_00366	YP_498955.1	3919785	nfrA	NADPH-dependent oxidoreductase (EC 1.6.-.-)	120.6547299	1.811603177	0.275938102	2.941250848	0.003268897	0.021784392	#N/A
SAOUHSC_00107	YP_498707.1	3919816		Uncharacterized protein	98.74460431	-2.075539871	0.366902864	-2.931402222	0.003374356	0.022393527	#N/A
SAOUHSC_T0001		3921517			23.02221405	3.096938635	0.715506839	2.930703833	0.00338195	0.022393527	Cluster 6
SAOUHSC_02108	YP_500600.1	3921180	ftnA	Bacterial non-heme ferritin (EC 1.16.3.2)	744.5017834	2.615817664	0.551406954	2.930354164	0.003385759	0.022393527	Cluster 4
SAOUHSC_T00059		3921067			14.12523794	2.925314446	0.657749645	2.927123505	0.00342113	0.022570905	Cluster 5
SAOUHSC_01085	YP_499630.1	3919246		conserved hypothetical protein	35.98296946	2.687730202	0.577145415	2.924272045	0.003452629	0.022721912	Cluster 6
SAOUHSC_00893	YP_499446.1	3921739		FMN oxidoreductase, putative	194.7913291	1.869810181	0.298204123	2.91682815	0.003536105	0.023213388	#N/A
SAOUHSC_T00027		3921153			151.608366	2.330307649	0.456857987	2.91186252	0.003592807	0.023527093	#N/A
SAOUHSC_01645	YP_500157.1	3920097		Uncharacterized protein	214.5684354	-1.780891694	0.268753634	-2.905604222	0.003665448	0.023943357	#N/A
SAOUHSC_01477	YP_499995.1	3920232		Uncharacterized protein	2993.914471	1.749861417	0.258401353	2.901925271	0.00370877	0.024166528	#N/A
SAOUHSC_03010	YP_501459.1	3921491	hisH	Imidazole glycerol phosphate synthase subunit HisH (EC 4.3.2.10) (IGP synthase glutaminase subunit) (EC 3.5.1.2) (IGP synthase subunit HisH) (ImGP synthase subunit HisH) (IGPS subunit HisH)	48.80390563	2.242525293	0.430867364	2.883776762	0.003929372	0.025540918	#N/A
SAOUHSC_T00013		3921332			35.01336432	2.551104274	0.538658358	2.87956968	0.003982183	0.025820591	Cluster 5
SAOUHSC_00177	YP_498774.1	3919491		Maltose ABC transporter, permease protein, putative	155.9252346	-2.814101062	0.631315189	-2.873526716	0.004059167	0.026255252	#N/A
SAOUHSC_00866	YP_499419.1	3918996		D-isomer specific 2-hydroxyacid dehydrogenase, NAD binding domain protein	171.6222991	-1.940782532	0.328150239	-2.866926247	0.004144796	0.026743561	#N/A
SAOUHSC_00828	YP_499383.1	3918941		Uncharacterized protein	295.9287716	1.926217701	0.324918905	2.850611908	0.004363519	0.028086164	#N/A
SAOUHSC_T00053		3921042			37.64931753	2.227199401	0.431161755	2.84626219	0.004423576	0.028403445	Cluster 5
SAOUHSC_02990	YP_501439.1	3921472		conserved hypothetical protein	671.3938806	-2.535890154	0.543633249	-2.82523219	0.004724637	0.030262902	#N/A

SAOUHSC_02261	YP_500743.1	3919137	agrB	Accessory gene regulator protein B (EC 3.4.-.-)	95.78304712	2.017709428	0.360492618	2.823107539	0.004756062	0.030390427	#N/A
SAOUHSC_02587	YP_501049.1	3921583		Uncharacterized protein	52.40301645	2.067879502	0.382280333	2.79344609	0.005214972	0.033242298	Cluster 5
SAOUHSC_01330	YP_499860.1	3920195	guaC	GMP reductase (EC 1.7.1.7) (Guanosine 5'-monophosphate oxidoreductase) (Guanosine monophosphate reductase)	102.6156949	2.412021362	0.506977018	2.785178247	0.005349831	0.034019766	Cluster 5
SAOUHSC_00434	YP_499017.1	3919106		Transcriptional regulator, lysR family, putative	27.48045821	2.230906008	0.443620974	2.774679466	0.005525614	0.035053112	Cluster 5
SAOUHSC_02550	YP_501012.1	3921138	fdhD	Sulfur carrier protein FdhD	62.04335633	2.276306116	0.460165598	2.773580037	0.00554432	0.035087435	#N/A
SAOUHSC_01633	YP_500146.1	3919970	gcvPA	Probable glycine dehydrogenase (decarboxylating) subunit 1 (EC 1.4.4.2) (Glycine cleavage system P-protein subunit 1) (Glycine decarboxylase subunit 1) (Glycine dehydrogenase (aminomethyl-transferring) subunit 1)	268.7588855	1.715139018	0.258415624	2.767398526	0.005650563	0.03567425	Cluster 6
SAOUHSC_01806	YP_500311.1	3919276	pyk	Pyruvate kinase (PK) (EC 2.7.1.40)	1797.414785	-2.047127324	0.378799725	-2.764329682	0.005703988	0.035841252	#N/A
SAOUHSC_02647	YP_501109.1	3921209	mqo	Probable malate:quinone oxidoreductase (EC 1.1.5.4) (MQO) (Malate dehydrogenase [quinone])	419.9405481	-1.84481884	0.305615561	-2.764318794	0.005704178	0.035841252	Cluster 1
SAOUHSC_01893	YP_500394.1	3920840		Arsenical pump membrane protein	80.27410616	2.410715116	0.510665229	2.762504738	0.005735973	0.035955422	Cluster 5
SAOUHSC_00714	YP_499273.1	3919341	saeS	Histidine protein kinase SaeS (EC 2.7.13.3) (Sensor protein SaeS) (Staphylococcus exoprotein expression protein S)	562.5607449	-1.666210683	0.241670713	-2.756687701	0.005839009	0.036514561	#N/A
SAOUHSC_00533	YP_499105.1	3920386	hchA	Protein/nucleic acid deglycase HchA (EC 3.1.2.-) (EC 3.5.1.-) (EC 3.5.1.124) (Maillard deglycase)	1116.47415	2.060783374	0.386128764	2.74722702	0.006010152	0.037459673	Cluster 5
SAOUHSC_00926	YP_499479.1	3920813		Oligopeptide ABC transporter, ATP-binding protein, putative	456.0750262	-2.564326878	0.569515034	-2.746770119	0.00601853	0.037459673	Cluster 2
SAOUHSC_02891	YP_501344.1	3921343		Uncharacterized protein	56.30796802	2.067142699	0.38897849	2.743449126	0.006079747	0.037751651	Cluster 6
SAOUHSC_00717	YP_499276.1	3919344		Uncharacterized protein	206.4993598	-2.062862894	0.388911046	-2.732920301	0.006277552	0.038888403	#N/A
SAOUHSC_00266	YP_498859.1	3919207		Uncharacterized protein	18.89887924	-3.238216865	0.819877524	-2.72994051	0.006334576	0.039149754	Cluster 1
SAOUHSC_01667	YP_500178.1	3920079	recO	DNA repair protein RecO (Recombination protein O)	192.9427175	-1.687621811	0.25200938	-2.728556413	0.006361221	0.039222575	Cluster 1
SAOUHSC_00406	YP_498992.1	3920564		Uncharacterized protein	84.08899492	-2.140143291	0.418911354	-2.721681519	0.00649507	0.03988047	#N/A
SAOUHSC_01894	YP_500395.1	3920841	arsC	Arsenate reductase (EC 1.20.4.4)	24.74372802	2.344666584	0.494085673	2.721525148	0.006498144	0.03988047	Cluster 5
SAOUHSC_T00057		3921046			133.5771983	2.091940946	0.402419987	2.713436164	0.00665894	0.040759247	#N/A
SAOUHSC_03030	YP_501478.1	3921296		Sodium, sulfate symporter, putative	151.0050325	-2.268835873	0.467726029	-2.712775842	0.006672222	0.040759247	#N/A
SAOUHSC_01985	YP_500482.1	3920461		Uncharacterized protein	21.25438751	2.307040307	0.483796494	2.701632447	0.006899999	0.042053344	Cluster 6
SAOUHSC_01010	YP_499560.1	3920271	purC	Phosphoribosylaminoimidazole-succinocarboxamide synthase (EC 6.3.2.6) (SAICAR synthetase)	33.98492179	2.057886484	0.393675944	2.687201237	0.007205353	0.043712474	Cluster 6
SAOUHSC_01128	YP_499671.1	3920728	argF	Ornithine carbamoyltransferase (OTCase) (EC 2.1.3.3)	37.96727479	-3.381564644	0.886062438	-2.687806796	0.0071923	0.043712474	#N/A
SAOUHSC_01388	YP_499915.1	3920679		Uncharacterized protein	2.119249507	3.789748999	1.041020638	2.67982103	0.007366153	0.044585501	#N/A
SAOUHSC_01261	YP_499794.1	3919992	cinA	Putative competence-damage inducible protein	106.9913316	1.630370627	0.23537579	2.678145555	0.007403104	0.044706616	#N/A
SAOUHSC_00202	YP_498799.1	3920358		Uncharacterized protein	24.90117329	-2.844623371	0.692444631	-2.663929055	0.007723385	0.046534276	Cluster 1
SAOUHSC_02328	YP_500807.1	3920953	thiE	Thiamine-phosphate synthase (TP synthase) (TPS) (EC 2.5.1.3) (Thiamine-phosphate pyrophosphorylase) (TMP pyrophosphorylase) (TMP-PPase)	48.05570218	-2.76449922	0.66326054	-2.660341016	0.007806157	0.046925851	Cluster 1

SAOUHSC_02422	YP_500893.1	3919632	Uncharacterized protein	365.4117577	-1.53960354	0.202994518	-2.658217299	0.007855522	0.047115281	Cluster 1
SAOUHSC_00716	YP_499275.1	3919343	Uncharacterized protein	339.1363865	-1.994304998	0.374502156	-2.65500474	0.007930729	0.047458492	#N/A
SAOUHSC_00927	YP_499480.1	3920756	Oligopeptide ABC transporter, substrate-binding protein, putative	854.6636938	-2.475407844	0.556378499	-2.651806006	0.008006253	0.047802038	Cluster 2

HUVEC – 1 hpi vs. uninfected

Gene_id	Name	Description	baseMean	log2FoldChange	lfcSE	stat	pvalue	padj	Cluster
ENSG00000137834	SMAD6	SMAD family member 6	674.9451836	3.981444431	0.10113655	29.47939626	5.2895E-191	1.2905E-186	Cluster 1
ENSG00000123358	NR4A1	nuclear receptor subfamily 4 group A member 1	305.3721265	4.890970653	0.157025653	24.77920381	1.5025E-135	1.8328E-131	Cluster 1
ENSG00000099860	GADD45B	growth arrest and DNA damage inducible beta	285.905419	3.365155196	0.127050516	18.61586455	2.38972E-77	1.9434E-73	Cluster 1
ENSG00000184557	SOCS3	suppressor of cytokine signaling 3	442.6331569	2.709056213	0.099976347	17.09460553	1.62794E-65	9.9292E-62	Cluster 1
ENSG00000134107	BHLHE40	basic helix-loop-helix family member e40	440.0764106	2.838064742	0.111917367	16.42340945	1.30049E-60	6.34563E-57	Cluster 1
ENSG00000101665	SMAD7	SMAD family member 7	193.0112481	4.185522219	0.195910827	16.26006214	1.89546E-59	7.70724E-56	Cluster 1
ENSG00000164683	HEY1	hes-related family bHLH transcription factor with YRPW motif 1	572.044014	3.34690483	0.144971552	16.18872668	6.0569E-59	2.111E-55	Cluster 1
ENSG00000119508	NR4A3	nuclear receptor subfamily 4 group A member 3	67.78521619	5.52736599	0.304531125	14.86667741	5.42392E-50	1.65409E-46	Cluster 1
ENSG00000125968	ID1	inhibitor of DNA binding 1, dominant negative helix-loop-helix protein	924.5457356	3.113625806	0.143007692	14.77980501	1.97738E-49	5.36023E-46	Cluster 1
ENSG00000168209	DDIT4	DNA damage inducible transcript 4	357.1116247	2.556959569	0.111448754	13.97018376	2.37014E-44	5.78244E-41	Cluster 1
ENSG00000124216	SNAI1	snail family zinc finger 1	98.26041099	3.883020665	0.209167493	13.78331126	3.21195E-43	7.12381E-40	Cluster 1
ENSG00000144655	CSRNP1	cysteine-serine-rich nuclear protein 1	373.7582883	2.349231164	0.099603924	13.54596395	8.37078E-42	1.70185E-38	Cluster 1
ENSG00000127528	KLF2	Kruppel-like factor 2	179.5183644	2.902645064	0.144836852	13.13647069	2.03499E-39	3.81905E-36	Cluster 1
ENSG00000168874	ATOH8	atonal bHLH transcription factor 8	132.4802169	3.146969551	0.167849717	12.79102277	1.84036E-37	3.20709E-34	Cluster 1
ENSG00000115738	ID2	inhibitor of DNA binding 2, dominant negative helix-loop-helix protein	66.89790507	3.889105858	0.226871346	12.734556	3.80002E-37	6.1806E-34	Cluster 1
ENSG00000259884	RP11-1100L3.8		55.97674707	4.627103848	0.286072671	12.67895965	7.73517E-37	1.17947E-33	Cluster 1
ENSG00000136826	KLF4	Kruppel-like factor 4 (gut)	178.2964331	3.796465332	0.228366366	12.24552187	1.77531E-34	2.54778E-31	Cluster 1
ENSG00000158050	DUSP2	dual specificity phosphatase 2	49.07874043	4.483489322	0.285474728	12.20244378	3.01633E-34	4.0883E-31	Cluster 1
ENSG00000267519	CTD-3252C9.4	uncharacterized LOC284454	251.7864741	2.62551686	0.137839278	11.79284227	4.24953E-32	5.45662E-29	Cluster 1
ENSG00000155090	KLF10	Kruppel-like factor 10	541.7084343	2.795486644	0.1559153	11.51578226	1.09858E-30	1.3401E-27	Cluster 1
ENSG00000269926	RP11-442H21.2		227.8929107	2.415322514	0.12302797	11.50407114	1.25837E-30	1.46192E-27	Cluster 1
ENSG00000171223	JUNB	jun B proto-oncogene	671.0948894	2.725939037	0.156906709	10.99977845	3.83072E-28	4.24809E-25	Cluster 1
ENSG00000120129	DUSP1	dual specificity phosphatase 1	806.1876751	2.008467527	0.092941402	10.85057369	1.98178E-27	2.10215E-24	Cluster 1
ENSG00000125740	FOSB	FBJ murine osteosarcoma viral oncogene homolog B	264.1794142	2.475756166	0.153045459	9.642600126	5.28325E-22	5.37064E-19	Cluster 1
ENSG00000120738	EGR1	early growth response 1	153.2056312	2.512169694	0.160202618	9.439107258	3.75969E-21	3.66901E-18	Cluster 1
ENSG00000274995	RP11-321F6.2		35.87812511	3.597300218	0.275309354	9.434115406	3.94307E-21	3.69997E-18	Cluster 1
ENSG00000275993	CH507-42P11.8	serine/threonine-protein kinase SIK1	40.60013486	3.424293253	0.262670809	9.229397282	2.72159E-20	2.45921E-17	Cluster 1
ENSG00000187678	SPRY4	sprouty RTK signaling antagonist 4	194.9739607	2.230409976	0.136636584	9.004981959	2.15701E-19	1.87945E-16	Cluster 1
ENSG00000183337	BCOR	BCL6 corepressor	197.4392785	2.107353628	0.124156656	8.919003314	4.70503E-19	3.95823E-16	Cluster 1
ENSG00000188483	IER5L	immediate early response 5-like	74.65353468	2.918771318	0.219357607	8.747229441	2.18655E-18	1.77817E-15	Cluster 1
ENSG00000106003	LFNG	LFNG O-fucosylpeptide 3-beta-N-acetylglucosaminyltransferase	64.22492354	2.94963925	0.222995191	8.742965461	2.2707E-18	1.78704E-15	Cluster 1
ENSG00000104419	NDRG1	N-myc downstream regulated 1	192.4564003	1.999688922	0.12142681	8.232851734	1.82808E-16	1.39374E-13	Cluster 1
ENSG00000179388	EGR3	early growth response 3	28.4407037	3.520756201	0.308627781	8.16762572	3.14518E-16	2.32524E-13	Cluster 1
ENSG00000160888	IER2	immediate early response 2	807.2127868	1.845600725	0.104154749	8.118695837	4.7122E-16	3.38128E-13	Cluster 1
ENSG00000163659	TIPARP	TCDD-inducible poly(ADP-ribose) polymerase	608.9780369	1.904443682	0.1123002	8.053802971	8.02604E-16	5.59461E-13	Cluster 1

ENSG00000166886	NAB2	NGFI-A binding protein 2 (EGR1 binding protein 2)	133.2573258	2.135471397	0.141071848	8.048887259	8.35502E-16	5.66215E-13	Cluster 1
ENSG00000198517	MAFK	v-maf avian musculoaponeurotic fibrosarcoma oncogene homolog K	443.6311045	1.711187631	0.09512421	7.476410347	7.63802E-14	5.03635E-11	Cluster 1
ENSG00000165507	C10orf10	chromosome 10 open reading frame 10	1750.765607	-1.883396618	0.119354555	-7.401448693	1.34707E-13	8.64853E-11	Cluster 2
ENSG00000168453	HR	hair growth associated	18.26651729	3.433851324	0.35044678	6.944995548	3.78472E-12	2.36759E-09	Cluster 1
ENSG00000131196	NFATC1	nuclear factor of activated T-cells, cytoplasmic, calcineurin-dependent 1	116.6279011	2.275325409	0.184162352	6.925006094	4.35957E-12	2.65901E-09	Cluster 1
ENSG00000165943	MOAP1	modulator of apoptosis 1	206.7542696	1.792749339	0.114942531	6.896919092	5.31424E-12	3.16223E-09	Cluster 1
ENSG00000183535	COL18A1-AS1	COL18A1 antisense RNA 1	142.1293702	1.893683479	0.133322691	6.703161124	2.03958E-11	1.18476E-08	Cluster 1
ENSG00000183691	NOG	noggin	19.68092596	3.111918457	0.320270947	6.594161832	4.27665E-11	2.37535E-08	Cluster 1
ENSG00000167470	MIDN	midnolin	385.4685528	1.704618909	0.106859056	6.593909141	4.28394E-11	2.37535E-08	Cluster 1
ENSG00000117318	ID3	inhibitor of DNA binding 3, dominant negative helix-loop-helix protein	1170.831974	1.598421063	0.091514661	6.53907318	6.19012E-11	3.35601E-08	Cluster 1
ENSG00000006459	KDM7A	lysine (K)-specific demethylase 7A	181.0438746	1.808844096	0.124929534	6.474402575	9.51878E-11	5.04847E-08	Cluster 1
ENSG00000176907	C8orf4	chromosome 8 open reading frame 4	156.8549613	1.906666613	0.140270231	6.463713692	1.02164E-10	5.30319E-08	Cluster 1
ENSG00000119669	IRF2BPL	interferon regulatory factor 2 binding protein-like	201.8293169	1.911676944	0.14315203	6.36859251	1.90771E-10	9.69632E-08	Cluster 1
ENSG00000198576	ARC	activity-regulated cytoskeleton-associated protein	14.35283969	3.262053726	0.358955223	6.301771305	2.94263E-10	1.46513E-07	Cluster 1
ENSG00000197019	SERTAD1	SERTA domain containing 1	122.2864114	1.907841573	0.148086192	6.130494424	8.76064E-10	4.27467E-07	Cluster 1
ENSG00000179348	GATA2	GATA binding protein 2	240.8853556	1.668857299	0.113754191	5.879847525	4.10645E-09	1.96441E-06	Cluster 1
ENSG00000267598	CTC-250I14.6		516.6607016	1.696186888	0.121419646	5.733725222	9.82485E-09	4.60955E-06	Cluster 1
ENSG00000255112	CHMP1B	charged multivesicular body protein 1B	407.872288	1.613311285	0.107107793	5.726112605	1.02758E-08	4.73017E-06	Cluster 1
ENSG00000128594	LRRC4	leucine rich repeat containing 4	49.72652253	2.187425809	0.216650856	5.480826757	4.23343E-08	1.91265E-05	Cluster 1
ENSG00000095066	HOOK2	hook microtubule-tethering protein 2	213.5378429	1.86641951	0.160331918	5.403911593	6.52031E-08	2.89229E-05	Cluster 1
ENSG00000187479	C11orf96	chromosome 11 open reading frame 96	32.15503537	2.417384006	0.267100088	5.306565095	1.1171E-07	4.86678E-05	Cluster 1
ENSG00000172201	ID4	inhibitor of DNA binding 4, dominant negative helix-loop-helix protein	8.228193539	2.96515269	0.37828039	5.194963156	2.0476E-07	8.7641E-05	Cluster 1
ENSG00000279637	RP11-196B3.1		12.99036294	2.843768253	0.35638586	5.173516848	2.29728E-07	9.66325E-05	Cluster 1
ENSG00000224574	COL18A1-AS2	COL18A1 antisense RNA 2	85.35670516	1.881816036	0.170707787	5.165646216	2.3961E-07	9.90806E-05	Cluster 1
ENSG00000184307	ZDHHC23	zinc finger, DHHC-type containing 23	57.27885591	2.119354245	0.225913033	4.954801544	7.24042E-07	0.000289581	Cluster 1
ENSG00000204055	RP11-247A12.2		36.52073983	2.359680152	0.274282446	4.957226289	7.15067E-07	0.000289581	Cluster 1
ENSG00000136603	SKIL	SKI-like proto-oncogene	1262.333198	1.452237186	0.092185788	4.905714808	9.30877E-07	0.0003663	Cluster 1
ENSG00000153234	NR4A2	nuclear receptor subfamily 4 group A member 2	56.26448902	2.083872532	0.224789637	4.821719302	1.42326E-06	0.000551164	Cluster 1
ENSG00000100906	NFKBIA	nuclear factor of kappa light polypeptide gene enhancer in B-cells inhibitor, alpha	446.213261	1.735130502	0.153274482	4.796170179	1.61728E-06	0.000616512	Cluster 1
ENSG00000161835	GRASP	GRP1 (general receptor for phosphoinositides 1)-associated scaffold protein	390.8042068	1.457628725	0.098278991	4.656424757	3.21748E-06	0.001207643	Cluster 1
ENSG00000117525	F3	coagulation factor III (thromboplastin, tissue factor)	43.23278759	2.314236276	0.285282679	4.60678608	4.0894E-06	0.001511654	Cluster 1
ENSG00000181472	ZBTB2	zinc finger and BTB domain containing 2	74.86083751	1.864594228	0.189044989	4.573483964	4.7968E-06	0.001746681	Cluster 1
ENSG00000122863	CHST3	carbohydrate (chondroitin 6) sulfotransferase 3	95.92899446	1.725543812	0.161003833	4.506376026	6.59442E-06	0.002365942	Cluster 1
ENSG00000138166	DUSP5	dual specificity phosphatase 5	289.2508738	1.87195128	0.19730668	4.419268919	9.90354E-06	0.003501689	Cluster 1
ENSG00000075426	FOSL2	FOS-like antigen 2	749.1466481	1.412037265	0.095178208	4.329113515	1.49711E-05	0.005217848	Cluster 1
ENSG00000171604	CXXC5	CXXC finger protein 5	49.15066334	2.026095279	0.245680385	4.17654539	2.9597E-05	0.010170103	Cluster 1

ENSG00000106089	STX1A	syntaxin 1A	118.839296	1.603929783	0.14814826	4.07652297	4.57141E-05	0.01549011	Cluster 1
ENSG00000118515	SGK1	serum/glucocorticoid regulated kinase 1	835.5059714	1.50813358	0.127098282	3.997957895	6.38913E-05	0.021352827	Cluster 1
ENSG00000197461	PDGFA	platelet-derived growth factor alpha polypeptide	149.4254818	1.636069473	0.160789755	3.955907977	7.62445E-05	0.02513699	Cluster 1
ENSG00000034152	MAP2K3	mitogen-activated protein kinase kinase 3	122.3304251	1.62408157	0.160449733	3.889576865	0.000100419	0.032665683	Cluster 1
ENSG00000235831	BHLHE40-AS1	BHLHE40 antisense RNA 1	23.08035673	2.156673929	0.299117912	3.866949727	0.000110205	0.035377298	Cluster 1
ENSG00000108551	RASD1	RAS, dexamethasone-induced 1	38.8493548	1.911285904	0.236690526	3.85011568	0.000118062	0.037407271	Cluster 1
ENSG00000267165	RP11-78A19.3		160.5788058	1.567680688	0.14891797	3.81203618	0.000137827	0.043109717	Cluster 1

HUVEC – 16 hpi vs. 1 hpi

Gene_id	Name	Description	baseMean	log2FoldChange	lfcSE	stat	pvalue	padj	Cluster
ENSG00000137834	SMAD6	SMAD family member 6	674.9451836	-3.562797516	0.117484291	-21.81395912	1.7102E-105	3.0763E-101	Cluster 1
ENSG00000119917	IFIT3	interferon induced protein with tetratricopeptide repeats 3	1394.543353	6.166134813	0.27951919	18.48221874	2.87127E-76	2.58242E-72	Cluster 2
ENSG00000140853	NLRC5	NLR family, CARD domain containing 5	419.1782563	4.579751357	0.204000023	17.54779878	6.18303E-69	3.70735E-65	Cluster 2
ENSG00000128284	APOL3	apolipoprotein L, 3	452.955401	3.845172741	0.165723449	17.16819648	4.59476E-66	2.06626E-62	Cluster 2
ENSG00000152778	IFIT5	interferon induced protein with tetratricopeptide repeats 5	391.3525165	3.660575217	0.157578345	16.8841424	5.88655E-64	2.11774E-60	Cluster 2
ENSG00000184979	USP18	ubiquitin specific peptidase 18	334.7952188	3.860069374	0.176324734	16.22046608	3.61442E-59	1.0836E-55	Cluster 2
ENSG00000165507	C10orf10	chromosome 10 open reading frame 10	1750.765607	2.856602894	0.119864941	15.48912363	4.10853E-54	1.05577E-50	Cluster 2
ENSG00000138496	PARP9	poly(ADP-ribose) polymerase family member 9	512.8227769	3.623858629	0.170489676	15.39013203	1.90643E-53	4.28661E-50	Cluster 2
ENSG00000123358	NR4A1	nuclear receptor subfamily 4 group A member 1	305.3721265	-3.418704111	0.157337517	-15.37271056	2.49508E-53	4.98684E-50	Cluster 1
ENSG00000135899	SP110	SP110 nuclear body protein	217.9349397	4.492065332	0.228136771	15.3068938	6.87707E-53	1.23705E-49	Cluster 2
ENSG00000185885	IFITM1	interferon induced transmembrane protein 1	181.8742945	5.170855076	0.272949675	15.28067425	1.02871E-52	1.68222E-49	Cluster 2
ENSG00000257452	RP1-71H24.1		107.7353748	5.440308438	0.291022784	15.2575973	1.46546E-52	2.19672E-49	Cluster 2
ENSG00000099860	GADD45B	growth arrest and DNA damage inducible beta	285.905419	-3.422670737	0.159026532	-15.23438077	2.09099E-52	2.89328E-49	Cluster 1
ENSG00000068079	IFI35	interferon induced protein 35	274.7578354	4.619886162	0.240190504	15.07089621	2.51676E-51	3.23367E-48	Cluster 2
ENSG00000089127	OAS1	2'-5'-oligoadenylate synthetase 1	432.480613	5.661250168	0.309566831	15.05733075	3.09014E-51	3.70569E-48	Cluster 2
ENSG00000101347	SAMHD1	SAM domain and HD domain 1	537.7034743	3.231628737	0.148531879	15.02457758	5.06834E-51	5.69808E-48	Cluster 2
ENSG00000128335	APOL2	apolipoprotein L, 2	631.6611184	3.369435377	0.158559805	14.94348069	1.71764E-50	1.81747E-47	Cluster 2
ENSG00000168961	LGALS9	lectin, galactoside-binding, soluble, 9	261.3994593	3.508910322	0.171640992	14.61719773	2.18198E-48	2.18053E-45	Cluster 2
ENSG00000263069	CTD-2047H16.4	uncharacterized LOC100294362	415.8456059	3.585288592	0.179159675	14.43008086	3.34689E-47	3.01019E-44	Cluster 2
ENSG00000130589	HELZ2	helicase with zinc finger 2, transcriptional coactivator	2035.697532	4.123959994	0.216482931	14.43051412	3.32593E-47	3.01019E-44	Cluster 2
ENSG00000134326	CMPK2	cytidine monophosphate (UMP-CMP) kinase 2, mitochondrial	531.4512502	5.049627441	0.288568038	14.0335273	9.71946E-45	8.32541E-42	Cluster 2
ENSG00000130303	BST2	bone marrow stromal cell antigen 2	397.1495681	3.443082447	0.174605102	13.99204504	1.74326E-44	1.42535E-41	Cluster 2
ENSG00000130813	C19orf66	chromosome 19 open reading frame 66	348.5192544	3.382414254	0.17394466	13.69639205	1.06696E-42	8.34453E-40	Cluster 2
ENSG00000101665	SMAD7	SMAD family member 7	193.0112481	-4.24210079	0.23731558	-13.66155895	1.72256E-42	1.29106E-39	Cluster 1
ENSG00000119922	IFIT2	interferon induced protein with tetratricopeptide repeats 2	920.0274502	5.584192354	0.3375118	13.58231729	5.09874E-42	3.66864E-39	Cluster 2
ENSG00000060491	OGFR	opioid growth factor receptor	426.9608299	2.480699909	0.110059228	13.45366437	2.92985E-41	2.02701E-38	Cluster 2
ENSG00000187608	ISG15	ISG15 ubiquitin-like modifier	1434.920784	4.390123133	0.255275294	13.28026333	3.01333E-40	2.00754E-37	Cluster 2
ENSG00000181381	DDX60L	DEAD (Asp-Glu-Ala-Asp) box polypeptide 60-like	1813.790747	3.77342923	0.210218789	13.19306066	9.61988E-40	6.18008E-37	Cluster 2
ENSG00000280755	AC009950.1	SP110 nuclear body protein	150.438364	4.241663299	0.24765575	13.08939241	3.78624E-39	2.34852E-36	Cluster 2
ENSG00000185507	IRF7	interferon regulatory factor 7	208.6698546	3.366813198	0.180930625	13.08132989	4.21009E-39	2.52437E-36	Cluster 2
ENSG00000002549	LAP3	leucine aminopeptidase 3	1368.79701	2.524792422	0.117155669	13.01509722	1.00414E-38	5.79997E-36	Cluster 2
ENSG00000111331	OAS3	2'-5'-oligoadenylate synthetase 3	940.0457269	4.610156555	0.277426452	13.01302213	1.03179E-38	5.79997E-36	Cluster 2
ENSG00000225886	RP11-288L9.4		62.80870824	5.436656459	0.341050677	13.00878948	1.09056E-38	5.94454E-36	Cluster 2
ENSG00000184557	SOCS3	suppressor of cytokine signaling 3	442.6331569	-2.505050575	0.115903049	-12.98542695	1.4801E-38	7.83063E-36	Cluster 1
ENSG00000183486	MX2	MX dynamin-like GTPase 2	356.0366428	5.651022411	0.359341102	12.94319629	2.56722E-38	1.31941E-35	Cluster 2
ENSG00000120129	DUSP1	dual specificity phosphatase 1	806.1876751	-2.32586667	0.104573075	-12.67885327	7.74567E-37	3.87026E-34	Cluster 1

ENSG00000115415	STAT1	signal transducer and activator of transcription 1	2768.269437	2.824959453	0.144068196	12.66733048	8.97158E-37	4.36164E-34	Cluster 2
ENSG00000115267	IFIH1	interferon induced, with helicase C domain 1	628.4811053	4.646355748	0.288783356	12.62661326	1.50623E-36	7.13E-34	Cluster 2
ENSG00000178685	PARP10	poly(ADP-ribose) polymerase family member 10	437.1153032	2.724197018	0.13688579	12.59588029	2.22464E-36	1.02607E-33	Cluster 2
ENSG00000156587	UBE2L6	ubiquitin conjugating enzyme E2L 6	248.47387	3.067203665	0.164608538	12.55830158	3.57934E-36	1.60963E-33	Cluster 2
ENSG00000172183	ISG20	interferon stimulated exonuclease gene 20kDa	181.6549416	4.147419985	0.250715115	12.55377039	3.79023E-36	1.6629E-33	Cluster 2
ENSG00000173821	RNF213	ring finger protein 213	6329.405111	3.465900868	0.196546425	12.54614969	4.17313E-36	1.78729E-33	Cluster 2
ENSG00000134107	BHLHE40	basic helix-loop-helix family member e40	440.0764106	-2.58005518	0.12643387	-12.49708785	7.74363E-36	3.23936E-33	Cluster 1
ENSG00000280248	CTD-2047H16.3		224.3860078	3.509595843	0.207685746	12.08362101	1.28915E-33	5.27028E-31	Cluster 2
ENSG00000168874	ATOH8	atonal bHLH transcription factor 8	132.4802169	-3.744222553	0.227636502	-12.05528341	1.81906E-33	7.27138E-31	Cluster 1
ENSG00000168062	BATF2	basic leucine zipper transcription factor, ATF-like 2	83.01637811	4.823142775	0.317247188	12.05099026	1.91634E-33	7.49371E-31	Cluster 2
ENSG00000138642	HERC6	HECT and RLD domain containing E3 ubiquitin protein ligase family member 6	457.1481929	4.224616394	0.268871183	11.99316474	3.85875E-33	1.47684E-30	Cluster 2
ENSG00000221963	APOL6	apolipoprotein L, 6	631.5877576	4.683702862	0.310407182	11.86732483	1.74974E-32	6.55713E-30	Cluster 2
ENSG00000138035	PNPT1	polyribonucleotide nucleotidyltransferase 1	574.7822674	2.748553428	0.149083469	11.72868759	9.08565E-32	3.33536E-29	Cluster 2
ENSG00000121060	TRIM25	tripartite motif containing 25	866.691858	2.223956688	0.104583145	11.70319265	1.22747E-31	4.41594E-29	Cluster 2
ENSG00000119508	NR4A3	nuclear receptor subfamily 4 group A member 3	67.78521619	-4.891439234	0.332720343	-11.69582597	1.33878E-31	4.72196E-29	Cluster 1
ENSG00000157601	MX1	MX dynamin-like GTPase 1	3295.228182	5.118753758	0.352309548	11.69072421	1.42169E-31	4.91796E-29	Cluster 2
ENSG00000137965	IFI44	interferon induced protein 44	777.1401967	4.499301859	0.301990047	11.58747413	4.76992E-31	1.61889E-28	Cluster 2
ENSG00000228318	AP001610.5		733.0868016	5.121742537	0.356279033	11.56886078	5.92643E-31	1.97416E-28	Cluster 2
ENSG00000205413	SAMD9	sterile alpha motif domain containing 9	657.1221937	3.848622917	0.250754933	11.36018693	6.60033E-30	2.15867E-27	Cluster 2
ENSG00000111335	OAS2	2'-5'-oligoadenylate synthetase 2	764.0131342	4.983442866	0.35082349	11.35455004	7.04014E-30	2.26139E-27	Cluster 2
ENSG00000185745	IFIT1	interferon induced protein with tetratricopeptide repeats 1	1399.14554	5.152189004	0.369137566	11.24835126	2.35962E-29	7.44645E-27	Cluster 2
ENSG00000164683	HEY1	hes-related family bHLH transcription factor with YRPW motif 1	572.044014	-2.713515537	0.152512904	-11.23521675	2.73821E-29	8.49223E-27	Cluster 1
ENSG00000124201	ZNFX1	zinc finger, NFX1-type containing 1	962.249264	2.697199194	0.151624072	11.19346796	4.38924E-29	1.3382E-26	Cluster 2
ENSG00000125968	ID1	inhibitor of DNA binding 1, dominant negative helix-loop-helix protein	924.5457356	-2.644311275	0.14838671	-11.08125706	1.54685E-28	4.63747E-26	Cluster 1
ENSG00000067066	SP100	SP100 nuclear antigen	803.3625513	2.32886383	0.120053204	11.06895761	1.77453E-28	5.23282E-26	Cluster 2
ENSG00000173193	PARP14	poly(ADP-ribose) polymerase family member 14	3230.511171	3.529637657	0.229081567	11.04251946	2.38257E-28	6.91253E-26	Cluster 2
ENSG00000138646	HERC5	HECT and RLD domain containing E3 ubiquitin protein ligase 5	376.7845114	4.442732638	0.312537463	11.0154239	3.22017E-28	9.19436E-26	Cluster 2
ENSG00000225963	AC009950.2		45.38416995	4.508686056	0.322909997	10.86583285	1.67686E-27	4.71303E-25	Cluster 2
ENSG00000124216	SNAI1	snail family zinc finger 1	98.26041099	-3.722951963	0.252875722	-10.76794538	4.87765E-27	1.34983E-24	Cluster 1
ENSG00000107201	DDX58	DEAD (Asp-Glu-Ala-Asp) box polypeptide 58	1210.566667	3.928502501	0.272206264	10.75839496	5.41043E-27	1.47459E-24	Cluster 2
ENSG00000267387	CTD-2240E14.4		124.3228533	3.289002273	0.212972652	10.74786952	6.06462E-27	1.62821E-24	Cluster 2
ENSG00000267519	CTD-3252C9.4	uncharacterized LOC284454	251.7864741	-2.735645552	0.162129993	-10.70527123	9.61487E-27	2.54342E-24	Cluster 1
ENSG00000158050	DUSP2	dual specificity phosphatase 2	49.07874043	-4.593652108	0.337824825	-10.63761998	1.99151E-26	5.19179E-24	Cluster 1
ENSG00000188313	PLSCR1	phospholipid scramblase 1	465.2297432	2.53862913	0.145544383	10.57154592	4.03783E-26	1.03761E-23	Cluster 2
ENSG00000100342	APOL1	apolipoprotein L1	222.1961015	3.325405	0.220625162	10.54007156	5.64559E-26	1.43032E-23	Cluster 2
ENSG00000134321	RSAD2	radical S-adenosyl methionine domain containing 2	1354.259541	4.95319564	0.37547043	10.52864707	6.3744E-26	1.58599E-23	Cluster 2
ENSG00000170581	STAT2	signal transducer and activator of transcription 2	901.7025303	2.635576241	0.155358778	10.52773625	6.43637E-26	1.58599E-23	Cluster 2
ENSG00000240065	PSMB9	proteasome subunit beta 9	68.55825235	4.080954196	0.293299691	10.50445769	8.2395E-26	2.00287E-23	Cluster 2

ENSG00000204267	TAP2	transporter 2, ATP-binding cassette, sub-family B (MDR/TAP)	390.8166902	2.769836096	0.169268873	10.45576815	1.37877E-25	3.30684E-23	Cluster 2
ENSG00000125740	FOSB	FBJ murine osteosarcoma viral oncogene homolog B	264.1794142	-2.840623952	0.177366943	-10.37749157	3.13915E-25	7.42987E-23	Cluster 1
ENSG00000108771	DHX58	DEXH (Asp-Glu-X-His) box polypeptide 58	123.4589312	3.707047794	0.263036398	10.2915331	7.69414E-25	1.79743E-22	Cluster 2
ENSG00000276863	CTD-2047H16.5		147.6465321	3.15671211	0.211073721	10.21781442	1.65019E-24	3.8056E-22	Cluster 2
ENSG00000204261	PSMB8-AS1	PSMB8 antisense RNA 1 (head to head)	156.4121888	3.561598542	0.25227463	10.15400772	3.18029E-24	7.24139E-22	Cluster 2
ENSG00000132530	XAF1	XIAP associated factor 1	332.0599535	3.741330451	0.272144451	10.07307126	7.26726E-24	1.63404E-21	Cluster 2
ENSG00000055332	EIF2AK2	eukaryotic translation initiation factor 2 alpha kinase 2	954.9186499	2.516222751	0.151445563	10.01166839	1.35449E-23	3.00797E-21	Cluster 2
ENSG00000135114	OASL	2'-5'-oligoadenylate synthetase-like	1123.835101	4.752300058	0.37801059	9.926441636	3.19453E-23	7.0077E-21	Cluster 2
ENSG00000160932	LY6E	lymphocyte antigen 6 complex, locus E	332.6783787	2.499575736	0.151130696	9.922376949	3.32736E-23	7.21116E-21	Cluster 2
ENSG00000059378	PARP12	poly(ADP-ribose) polymerase family member 12	571.0561791	2.217271182	0.12275917	9.915928713	3.5494E-23	7.60079E-21	Cluster 2
ENSG00000177409	SAMD9L	sterile alpha motif domain containing 9-like	805.651111	3.464843461	0.254407882	9.688549901	3.37283E-22	7.13771E-20	Cluster 2
ENSG00000250264	XXbac-BPG246D15.9		251.6781398	2.656482654	0.17239673	9.608550314	7.35776E-22	1.53897E-19	Cluster 2
ENSG00000187678	SPRY4	sprouty RTK signaling antagonist 4	194.9739607	-2.616321609	0.168281695	-9.604856965	7.62637E-22	1.57682E-19	Cluster 1
ENSG00000133106	EPSTI1	epithelial stromal interaction 1 (breast)	437.8594816	4.55126232	0.371925246	9.548322833	1.31813E-21	2.69437E-19	Cluster 2
ENSG00000112773	FAM46A	family with sequence similarity 46 member A	73.06726952	3.410505033	0.25353666	9.507520665	1.95261E-21	3.94646E-19	Cluster 2
ENSG00000259884	RP11-1100L3.8		55.97674707	-3.967699273	0.314145063	-9.446907249	3.4899E-21	6.97514E-19	Cluster 1
ENSG00000101342	TLDC2	TBC/LysM-associated domain containing 2	65.56340199	3.265403356	0.242521725	9.341032681	9.5399E-21	1.88576E-18	Cluster 2
ENSG00000183337	BCOR	BCL6 corepressor	197.4392785	-2.448348884	0.155719766	-9.300995784	1.39136E-20	2.72042E-18	Cluster 1
ENSG00000155090	KLF10	Kruppel-like factor 10	541.7084343	-2.525476262	0.164312094	-9.284016919	1.63207E-20	3.15674E-18	Cluster 1
ENSG00000130812	ANGPTL6	angiopoietin like 6	96.75376562	3.26607005	0.245091582	9.245809371	2.33467E-20	4.46766E-18	Cluster 2
ENSG00000115738	ID2	inhibitor of DNA binding 2, dominant negative helix-loop-helix protein	66.89790507	-3.451250953	0.26588011	-9.219384446	2.98811E-20	5.6579E-18	Cluster 1
ENSG00000148677	ANKRD1	ankyrin repeat domain 1 (cardiac muscle)	1837.006254	-1.616757452	0.067219867	-9.175225651	4.5063E-20	8.44367E-18	Cluster 1
ENSG00000168394	TAP1	transporter 1, ATP-binding cassette, sub-family B (MDR/TAP)	688.4815401	3.522058832	0.277209377	9.098028557	9.19863E-20	1.70582E-17	Cluster 2
ENSG00000163644	PPM1K	protein phosphatase, Mg2+/Mn2+ dependent 1K	290.6755824	2.633670081	0.179840559	9.083991364	1.04665E-19	1.92113E-17	Cluster 2
ENSG00000118515	SGK1	serum/glucocorticoid regulated kinase 1	835.5059714	-2.221530638	0.136169463	-8.970664999	2.9473E-19	5.35515E-17	Cluster 1
ENSG00000131203	IDO1	indoleamine 2,3-dioxygenase 1	48.17855099	4.316677479	0.370655903	8.948130728	3.61553E-19	6.50361E-17	Cluster 2
ENSG00000197536	C5orf56		111.5178767	3.07053371	0.232568522	8.902897467	5.44074E-19	9.68991E-17	Cluster 2
ENSG00000169248	CXCL11	chemokine (C-X-C motif) ligand 11	699.2194082	4.294645839	0.37100054	8.880434086	6.65999E-19	1.17451E-16	Cluster 2
ENSG00000106003	LFNG	LFNG O-fucosylpeptide 3-beta-N-acetylglucosaminyltransferase	64.22492354	-3.527545678	0.285154765	-8.863767995	7.73546E-19	1.35093E-16	Cluster 1
ENSG00000234745	HLA-B	major histocompatibility complex, class I, B	781.498232	2.489327085	0.169943643	8.763652826	1.89023E-18	3.26938E-16	Cluster 2
ENSG00000132109	TRIM21	tripartite motif containing 21	164.9208847	2.46501526	0.168021706	8.719202372	2.80168E-18	4.79969E-16	Cluster 2
ENSG00000234290	AC116366.6	uncharacterized LOC105379175	47.52537818	3.512044432	0.291174853	8.627271234	6.2833E-18	1.06626E-15	Cluster 2
ENSG00000132470	ITGB4	integrin beta 4	90.12607484	2.915225172	0.222412573	8.611137155	7.23394E-18	1.21611E-15	Cluster 2
ENSG00000136514	RTP4	receptor (chemosensory) transporter protein 4	29.15871251	4.036607333	0.353357696	8.593579167	8.43014E-18	1.40409E-15	Cluster 2
ENSG00000132274	TRIM22	tripartite motif containing 22	1347.27451	2.247172052	0.145415775	8.5765939	9.77239E-18	1.61271E-15	Cluster 2
ENSG00000120738	EGR1	early growth response 1	153.2056312	-2.630402246	0.19023521	-8.570454667	1.03077E-17	1.68559E-15	Cluster 1
ENSG00000274995	RP11-321F6.2		35.87812511	-3.825124281	0.331478773	-8.522790942	1.55754E-17	2.52405E-15	Cluster 1
ENSG00000162654	GBP4	guanylate binding protein 4	1341.692457	3.846743585	0.336821365	8.451790421	2.86869E-17	4.60732E-15	Cluster 2

ENSG00000166750	SLFN5	schlafen family member 5	671.1521348	2.354319688	0.161133102	8.404974938	4.27953E-17	6.81241E-15	Cluster 2
ENSG00000275993	CH507-42P11.8	serine/threonine-protein kinase SIK1	40.60013486	-3.67792985	0.320121132	-8.365364183	5.99295E-17	9.45625E-15	Cluster 1
ENSG00000182179	UBA7	ubiquitin-like modifier activating enzyme 7	287.0727325	2.295452237	0.154977173	8.358987407	6.32589E-17	9.89479E-15	Cluster 2
ENSG00000140464	PML	promyelocytic leukemia	418.6767923	2.3619082	0.163172809	8.346416365	7.03654E-17	1.09115E-14	Cluster 2
ENSG00000188483	IER5L	immediate early response 5-like	74.65353468	-3.233505761	0.26771512	-8.342845037	7.25242E-17	1.11501E-14	Cluster 1
ENSG00000106785	TRIM14	tripartite motif containing 14	212.908226	2.226459855	0.14742281	8.319335737	8.84577E-17	1.34845E-14	Cluster 2
ENSG00000172936	MYD88	myeloid differentiation primary response 88	222.8247188	2.280722989	0.155600141	8.230860075	1.85874E-16	2.80966E-14	Cluster 2
ENSG00000126709	IFI6	interferon, alpha-inducible protein 6	835.6537911	3.676485003	0.32544024	8.224198105	1.96501E-16	2.94555E-14	Cluster 2
ENSG00000110446	SLC15A3	solute carrier family 15 (oligopeptide transporter), member 3	98.8390244	3.00223587	0.245615631	8.151907351	3.58229E-16	5.32547E-14	Cluster 2
ENSG00000141574	SECTM1	secreted and transmembrane 1	47.04872313	4.024250885	0.371851721	8.132948458	4.18973E-16	6.17744E-14	Cluster 2
ENSG00000165943	MOAP1	modulator of apoptosis 1	206.7542696	-2.160369289	0.144103478	-8.052333651	8.12301E-16	1.18794E-13	Cluster 1
ENSG00000137959	IFI44L	interferon induced protein 44 like	1784.087937	4.017728917	0.376155239	8.022562514	1.03562E-15	1.49172E-13	Cluster 2
ENSG00000265972	TXNIP	thioredoxin interacting protein	187.4592249	2.723376184	0.214819311	8.022445363	1.03661E-15	1.49172E-13	Cluster 2
ENSG00000179388	EGR3	early growth response 3	28.4407037	-3.799292791	0.351322626	-7.967869381	1.61433E-15	2.30465E-13	Cluster 1
ENSG00000155287	SLC25A28	solute carrier family 25 (mitochondrial iron transporter), member 28	155.0327851	2.659661746	0.209177534	7.934225595	2.11813E-15	3.00007E-13	Cluster 2
ENSG00000173786	CNP	2',3'-cyclic nucleotide 3' phosphodiesterase	1024.102411	1.955578068	0.121366303	7.873503967	3.44844E-15	4.84614E-13	Cluster 2
ENSG00000198517	MAFK	v-maf avian musculoaponeurotic fibrosarcoma oncogene homolog K	443.6311045	-1.858909069	0.110064164	-7.803712293	6.01121E-15	8.38215E-13	Cluster 1
ENSG00000131979	GCH1	GTP cyclohydrolase 1	143.3497883	2.971398345	0.252708183	7.801086313	6.13765E-15	8.49262E-13	Cluster 2
ENSG00000163840	DTX3L	deltex 3 like, E3 ubiquitin ligase	587.8976362	2.949708699	0.250773926	7.774766436	7.55867E-15	1.03329E-12	Cluster 2
ENSG00000204264	PSMB8	proteasome subunit beta 8	209.3800008	2.146285615	0.147444214	7.774368219	7.58249E-15	1.03329E-12	Cluster 2
ENSG00000266728	AC015688.3		106.8314222	2.409287551	0.191392728	7.363328624	1.7938E-13	2.42608E-11	Cluster 2
ENSG00000168016	TRANK1	tetratricopeptide repeat and ankyrin repeat containing 1	293.1633185	2.402890385	0.190663151	7.357952364	1.86753E-13	2.50695E-11	Cluster 2
ENSG00000185201	IFITM2	interferon induced transmembrane protein 2	207.0203681	2.516685645	0.207082215	7.324074851	2.40552E-13	3.20523E-11	Cluster 2
ENSG00000137198	GMPR	guanosine monophosphate reductase	193.6646801	2.493119529	0.204676441	7.295023899	2.98606E-13	3.9495E-11	Cluster 2
ENSG00000271581	XXbac-BPG248L24.12		230.4336802	2.565370865	0.215386817	7.267719009	3.65608E-13	4.77482E-11	Cluster 2
ENSG00000105939	ZC3HAV1	zinc finger CCCH-type, antiviral 1	697.5668898	2.197793583	0.164816019	7.267458518	3.66314E-13	4.77482E-11	Cluster 2
ENSG00000137628	DDX60	DEAD (Asp-Glu-Ala-Asp) box polypeptide 60	311.1276007	2.422040945	0.195800111	7.262717787	3.79389E-13	4.90968E-11	Cluster 2
ENSG00000144655	CSRNP1	cysteine-serine-rich nuclear protein 1	373.7582883	-1.779724803	0.110211576	-7.074799504	1.49665E-12	1.92298E-10	Cluster 1
ENSG00000204525	HLA-C	major histocompatibility complex, class I, C	772.0531872	2.057291958	0.149776291	7.059140994	1.67535E-12	2.13732E-10	Cluster 2
ENSG00000271503	CCL5	chemokine (C-C motif) ligand 5	24.53621412	3.632863691	0.37633579	6.996049168	2.63281E-12	3.33514E-10	Cluster 2
ENSG00000121858	TNFSF10	tumor necrosis factor superfamily member 10	843.7576164	3.173747691	0.311347754	6.981735575	2.91556E-12	3.66748E-10	Cluster 2
ENSG00000117228	GBP1	guanylate binding protein 1, interferon-inducible	579.6687074	3.155147716	0.309224936	6.969514641	3.18036E-12	3.9728E-10	Cluster 2
ENSG00000183691	NOG	noggin	19.68092596	-3.474636986	0.358496263	-6.902825053	5.09784E-12	6.32413E-10	Cluster 1
ENSG00000010030	ETV7	ets variant 7	25.25225197	3.557963965	0.371018292	6.894441652	5.40768E-12	6.66256E-10	Cluster 2
ENSG00000136158	SPRY2	sprouty RTK signaling antagonist 2	193.2179689	-2.073346463	0.155775407	-6.890346064	5.56568E-12	6.81058E-10	Cluster 1
ENSG00000204642	HLA-F	major histocompatibility complex, class I, F	64.58755191	2.937642684	0.28216753	6.866993827	6.55689E-12	7.96928E-10	Cluster 2
ENSG00000225864	HCG4P11		21.15026919	3.495153104	0.364170584	6.851605297	7.30258E-12	8.81603E-10	Cluster 2
ENSG00000166886	NAB2	NGFI-A binding protein 2 (EGR1 binding protein 2)	133.2573258	-2.1769804	0.172014899	-6.842316617	7.79226E-12	9.34448E-10	Cluster 1

ENSG00000117226	GBP3	guanylate binding protein 3	159.3868754	2.302830166	0.190804457	6.828090845	8.60521E-12	1.0251E-09	Cluster 2
ENSG00000043143	JADE2	jade family PHD finger 2	259.4342307	2.085901547	0.160250882	6.776259421	1.23327E-11	1.45948E-09	Cluster 2
ENSG00000075426	FOSL2	FOS-like antigen 2	749.1466481	-1.704699993	0.104546995	-6.740509327	1.57832E-11	1.85561E-09	Cluster 1
ENSG00000168209	DDIT4	DNA damage inducible transcript 4	357.1116247	-1.805997134	0.120069895	-6.71273288	1.91013E-11	2.23113E-09	Cluster 1
ENSG00000110057	UNC93B1	unc-93 homolog B1 (C. elegans)	140.832682	2.393295804	0.207950584	6.700129306	2.08235E-11	2.4166E-09	Cluster 2
ENSG00000105559	PLEKHA4	pleckstrin homology domain containing A4	71.82932436	2.78996286	0.268695559	6.661676385	2.70722E-11	3.12163E-09	Cluster 2
ENSG00000130513	GDF15	growth differentiation factor 15	417.6112509	-1.785363008	0.120016397	-6.543797557	5.9976E-11	6.87164E-09	Cluster 1
ENSG00000239713	APOBEC3G	apolipoprotein B mRNA editing enzyme, catalytic polypeptide-like 3G	46.97332891	3.116005945	0.324563075	6.519552311	7.05175E-11	8.02829E-09	Cluster 2
ENSG00000142961	MOB3C	MOB kinase activator 3C	108.2317261	2.163138401	0.180736359	6.435552897	1.23025E-10	1.3918E-08	Cluster 2
ENSG00000164342	TLR3	toll-like receptor 3	99.52457431	3.257950169	0.354000546	6.378380471	1.7897E-10	2.01208E-08	Cluster 2
ENSG00000173530	TNFRSF10D	tumor necrosis factor receptor superfamily member 10d, decoy with truncated death domain	793.7748272	-1.709330768	0.111504369	-6.361461683	1.99843E-10	2.23278E-08	Cluster 1
ENSG00000123609	NMI	N-myc and STAT interactor	137.4867522	2.23944925	0.195999513	6.323736376	2.55313E-10	2.83492E-08	Cluster 2
ENSG00000232133	IMPDH1P10		200.5752207	2.262316798	0.201240909	6.272664959	3.5492E-10	3.91675E-08	Cluster 2
ENSG00000155363	MOV10	Mov10 RISC complex RNA helicase	385.1859822	1.908994382	0.145365649	6.253158055	4.02235E-10	4.41183E-08	Cluster 2
ENSG00000130775	THEMIS2	thymocyte selection associated family member 2	60.86618117	2.765536663	0.284029658	6.216029254	5.09893E-10	5.55876E-08	Cluster 2
ENSG00000146859	TMEM140	transmembrane protein 140	194.14462	1.927876896	0.151027805	6.143748794	8.05963E-10	8.73353E-08	Cluster 2
ENSG00000262112	RP11-670E13.5		133.0556563	2.035230368	0.169924475	6.092297009	1.11302E-09	1.19886E-07	Cluster 2
ENSG00000154451	GBP5	guanylate binding protein 5	21.35526702	3.261118148	0.371936947	6.079305019	1.20705E-09	1.2924E-07	Cluster 2
ENSG00000160710	ADAR	adenosine deaminase, RNA-specific	1726.799075	1.720679455	0.118872828	6.062608815	1.33931E-09	1.42553E-07	Cluster 2
ENSG00000174640	SLCO2A1	solute carrier organic anion transporter family member 2A1	24.43114885	2.9478443	0.321840889	6.052196491	1.42884E-09	1.50304E-07	Cluster 2
ENSG00000006327	TNFRSF12A	tumor necrosis factor receptor superfamily member 12A	306.0748689	-1.858620591	0.141848995	-6.053060779	1.42119E-09	1.50304E-07	Cluster 1
ENSG00000279296	RP11-609D21.3		137.8440574	2.994311669	0.330601294	6.032377081	1.61565E-09	1.68967E-07	Cluster 2
ENSG00000004799	PDK4	pyruvate dehydrogenase kinase, isozyme 4	119.7633771	2.618443962	0.275604247	5.872347674	4.29666E-09	4.46754E-07	Cluster 2
ENSG00000170345	FOS	FBJ murine osteosarcoma viral oncogene homolog	298.5800129	-1.913961344	0.157833688	-5.790660776	7.011E-09	7.24793E-07	Cluster 1
ENSG00000184898	RBM43	RNA binding motif protein 43	72.04695544	2.236710376	0.214038764	5.777973832	7.56056E-09	7.7369E-07	Cluster 2
ENSG00000269926	RP11-442H21.2		227.8929107	-1.784829728	0.135836251	-5.777763458	7.57001E-09	7.7369E-07	Cluster 1
ENSG00000160888	IER2	immediate early response 2	807.2127868	-1.635509854	0.110379899	-5.757478127	8.53798E-09	8.67691E-07	Cluster 1
ENSG00000181472	ZBTB2	zinc finger and BTB domain containing 2	74.86083751	-2.3339018	0.232077215	-5.747663768	9.04849E-09	9.14406E-07	Cluster 1
ENSG00000133321	RARRES3	retinoic acid receptor responder (tazarotene induced) 3	15.1564096	3.116926007	0.372961346	5.675993049	1.37886E-08	1.38564E-06	Cluster 2
ENSG00000196776	CD47	CD47 molecule	483.8831496	1.881455151	0.15804606	5.57720422	2.44415E-08	2.44252E-06	Cluster 2
ENSG00000096696	DSP	desmoplakin	70.84729299	2.40832778	0.252616751	5.574958021	2.4759E-08	2.46058E-06	Cluster 2
ENSG00000204055	RP11-247A12.2		36.52073983	-2.759044148	0.317570691	-5.539063259	3.04094E-08	3.00552E-06	Cluster 1
ENSG00000185753	CXorf38	chromosome X open reading frame 38	206.1011199	1.889210764	0.162704637	5.465183891	4.62427E-08	4.52073E-06	Cluster 2
ENSG00000176907	C8orf4	chromosome 8 open reading frame 4	156.8549613	-1.896305832	0.163990368	-5.465600461	4.61342E-08	4.52073E-06	Cluster 1
ENSG00000004468	CD38	CD38 molecule	25.14643705	3.033538753	0.377933525	5.380678395	7.42057E-08	7.2152E-06	Cluster 2
ENSG00000125347	IRF1	interferon regulatory factor 1	344.1413453	2.086849386	0.202155715	5.376298113	7.60329E-08	7.35311E-06	Cluster 2
ENSG00000142089	IFITM3	interferon induced transmembrane protein 3	322.4807148	1.707755558	0.131703032	5.373874443	7.70625E-08	7.41284E-06	Cluster 2

ENSG00000169871	TRIM56	tripartite motif containing 56	225.7994339	1.747049108	0.139598269	5.351420985	8.72662E-08	8.34971E-06	Cluster 2
ENSG00000238025	ZDHHC4P1		13.81069942	2.959312344	0.373553658	5.245062662	1.56229E-07	1.48691E-05	Cluster 2
ENSG00000197461	PDGFA	platelet-derived growth factor alpha polypeptide	149.4254818	-1.969990832	0.186231973	-5.208508607	1.90365E-07	1.80225E-05	Cluster 1
ENSG00000102760	RGCC	regulator of cell cycle	102.9683351	-2.103909012	0.213095762	-5.180342404	2.21479E-07	2.08584E-05	Cluster 1
ENSG00000229023	AC067945.3		13.20065734	2.894604292	0.367400627	5.15678024	2.51232E-07	2.35373E-05	Cluster 2
ENSG00000132622	HSPA12B	heat shock protein family A (Hsp70) member 12B	58.41989996	2.212551334	0.237574893	5.103869849	3.32777E-07	3.10155E-05	Cluster 2
ENSG00000167470	MIDN	midnolin	385.4685528	-1.60563972	0.119204861	-5.080662944	3.7612E-07	3.48745E-05	Cluster 1
ENSG00000117318	ID3	inhibitor of DNA binding 3, dominant negative helix-loop-helix protein	1170.831974	-1.486827872	0.096493358	-5.045195681	4.53057E-07	4.17928E-05	Cluster 1
ENSG00000038210	PI4K2B	phosphatidylinositol 4-kinase type 2 beta	320.3848889	1.926270409	0.183813835	5.039176769	4.67539E-07	4.29086E-05	Cluster 2
ENSG00000156030	ELMSAN1	ELM2 and Myb/SANT-like domain containing 1	568.4852642	-1.518208703	0.104598396	-4.954270058	7.26023E-07	6.62929E-05	Cluster 1
ENSG00000269720	CTD-2521M24.5		9.880737249	2.864346063	0.376869699	4.946924813	7.53951E-07	6.84953E-05	Cluster 2
ENSG00000141682	PMAIP1	phorbol-12-myristate-13-acetate-induced protein 1	259.8845716	2.378373915	0.281232327	4.90119301	9.52564E-07	8.61041E-05	Cluster 2
ENSG00000108551	RASD1	RAS, dexamethasone-induced 1	38.8493548	-2.406251149	0.287281475	-4.895028989	9.82912E-07	8.84031E-05	Cluster 1
ENSG00000198576	ARC	activity-regulated cytoskeleton-associated protein	14.35283969	-2.799153639	0.36940893	-4.870357733	1.11396E-06	9.96915E-05	Cluster 1
ENSG00000213689	TREX1	three prime repair exonuclease 1	79.64528288	2.077449735	0.221970285	4.854026896	1.20979E-06	0.000107731	Cluster 2
ENSG00000141526	SLC16A3	solute carrier family 16 (monocarboxylate transporter), member 3	502.6031999	-1.67553501	0.139560771	-4.840436233	1.29554E-06	0.000114799	Cluster 1
ENSG00000163565	IFI16	interferon, gamma-inducible protein 16	951.145506	1.662650633	0.138045858	4.800221045	1.58491E-06	0.000139751	Cluster 2
ENSG00000034152	MAP2K3	mitogen-activated protein kinase kinase 3	122.3304251	-1.90606076	0.188861502	-4.797487845	1.60668E-06	0.00014098	Cluster 1
ENSG00000137842	TMEM62	transmembrane protein 62	140.7285858	1.827830443	0.173645514	4.767358647	1.86657E-06	0.000162989	Cluster 2
ENSG00000224574	COL18A1-AS2	COL18A1 antisense RNA 2	85.35670516	-1.966843531	0.204213795	-4.73446728	2.19631E-06	0.000190856	Cluster 1
ENSG00000070610	GBA2	glucosidase, beta (bile acid) 2	649.310775	-1.447513793	0.095227201	-4.699432389	2.60886E-06	0.000225616	Cluster 1
ENSG00000204138	PHACTR4	phosphatase and actin regulator 4	301.5269014	1.628098647	0.134694441	4.663137111	3.11425E-06	0.000268034	Cluster 2
ENSG00000171617	ENC1	ectodermal-neural cortex 1 (with BTB domain)	660.5194556	-1.409885354	0.088183935	-4.648072838	3.35051E-06	0.000286995	Cluster 1
ENSG00000166889	PATL1	protein associated with topoisomerase II homolog 1 (yeast)	197.408343	1.743939779	0.160216554	4.643339022	3.42823E-06	0.000292261	Cluster 2
ENSG00000224611	AC007919.18		10.17385468	2.727620163	0.375855402	4.596502145	4.29643E-06	0.000364548	Cluster 2
ENSG00000168453	HR	hair growth associated	18.26651729	-2.655022711	0.361195649	-4.58206713	4.60402E-06	0.000387101	Cluster 1
ENSG00000185722	ANKFY1	ankyrin repeat and FYVE domain containing 1	383.6505267	1.59951289	0.130840578	4.582010411	4.60527E-06	0.000387101	Cluster 2
ENSG00000225492	GBP1P1	guanylate binding protein 1, interferon-inducible pseudogene 1	10.48246095	2.711443258	0.376033534	4.551304879	5.33142E-06	0.000446054	Cluster 2
ENSG00000196116	TDRD7	tudor domain containing 7	175.0094745	1.860177277	0.19178684	4.485069346	7.28902E-06	0.000607014	Cluster 2
ENSG00000204592	HLA-E	major histocompatibility complex, class I, E	2824.041113	1.493181529	0.110017644	4.482749425	7.36874E-06	0.000610825	Cluster 2
ENSG00000277402	MIR6891	microRNA 6891	15.84701502	2.592491136	0.356223023	4.470489085	7.80409E-06	0.000643945	Cluster 2
ENSG00000165949	IFI27	interferon, alpha-inducible protein 27	519.7449245	2.359567614	0.304476378	4.46526467	7.99698E-06	0.000656848	Cluster 2
ENSG00000213928	IRF9	interferon regulatory factor 9	148.5322803	1.90234381	0.202567186	4.454540878	8.40729E-06	0.000687411	Cluster 2
ENSG00000135148	TRAFD1	TRAF-type zinc finger domain containing 1	296.9174084	1.905142845	0.203375896	4.450590571	8.56345E-06	0.00069701	Cluster 2
ENSG00000255112	CHMP1B	charged multivesicular body protein 1B	407.872288	-1.52627867	0.1185203	-4.440409522	8.97878E-06	0.000727524	Cluster 1
ENSG00000184307	ZDHHC23	zinc finger, DHHC-type containing 23	57.27885591	-2.142479819	0.258528306	-4.419167242	9.9082E-06	0.000799231	Cluster 1
ENSG00000106089	STX1A	syntaxin 1A	118.839296	-1.782298733	0.177581019	-4.405306025	1.05635E-05	0.000848284	Cluster 1
ENSG00000138670	RASGEF1B	RasGEF domain family member 1B	47.05669694	2.443241728	0.328171136	4.39783262	1.09337E-05	0.000874115	Cluster 2

ENSG00000244255	XXbac-BPG116M5.17		22.67599839	2.491805601	0.339798074	4.390270916	1.1321E-05	0.000901068	Cluster 2
ENSG00000216490	IFI30	interferon, gamma-inducible protein 30	19.5700805	2.651307616	0.377798825	4.370864875	1.23755E-05	0.000980666	Cluster 2
ENSG00000137752	CASP1	caspase 1	44.39741738	2.23210802	0.287243124	4.289425637	1.79136E-05	0.001413287	Cluster 2
ENSG00000179348	GATA2	GATA binding protein 2	240.8853556	-1.55939196	0.130763693	-4.277884386	1.88678E-05	0.00148207	Cluster 1
ENSG00000102921	N4BP1	NEDD4 binding protein 1	195.0511851	1.795129828	0.187368939	4.243658695	2.19905E-05	0.001719846	Cluster 2
ENSG00000131196	NFATC1	nuclear factor of activated T-cells, cytoplasmic, calcineurin-dependent 1	116.6279011	-1.855494587	0.201909154	-4.237027241	2.26499E-05	0.001763748	Cluster 1
ENSG00000185404	SP140L	SP140 nuclear body protein like	111.6466051	1.744889792	0.177419949	4.198455672	2.68741E-05	0.002081355	Cluster 2
ENSG00000169228	RAB24	RAB24, member RAS oncogene family	77.75896615	1.837994948	0.199630333	4.197733559	2.696E-05	0.002081355	Cluster 2
ENSG00000259529	RP11-468E2.4		291.3178963	1.527991015	0.126985755	4.157875927	3.2122E-05	0.002469279	Cluster 2
ENSG00000261884	CTC-479C5.12		135.8474075	1.935131284	0.225599611	4.145092626	3.39676E-05	0.002600042	Cluster 2
ENSG00000137200	CMTR1	cap methyltransferase 1	332.8999316	1.54883268	0.132586394	4.139434391	3.48163E-05	0.002653711	Cluster 2
ENSG00000225075	RP11-426L16.3		41.56134499	2.08772979	0.264943784	4.105511643	4.03421E-05	0.003061914	Cluster 2
ENSG00000172201	ID4	inhibitor of DNA binding 4, dominant negative helix-loop-helix protein	8.228193539	-2.513013215	0.371253567	-4.075417312	4.59319E-05	0.003471525	Cluster 1
ENSG00000281028	RP11-717K11.2	phosphatidylinositol 4-kinase type 2 beta	171.3090741	1.815512681	0.200668298	4.063983643	4.82422E-05	0.003630882	Cluster 2
ENSG00000277511	CTD-2095E4.5		9.434067971	2.506227919	0.375183036	4.014648252	5.95345E-05	0.004462112	Cluster 2
ENSG00000128394	APOBEC3F	apolipoprotein B mRNA editing enzyme, catalytic polypeptide-like 3F	27.35345852	2.203804008	0.300469159	4.006414541	6.16474E-05	0.004601302	Cluster 2
ENSG00000136147	PHF11	PHD finger protein 11	103.3452573	1.693003442	0.173572657	3.992584168	6.53571E-05	0.004858033	Cluster 2
ENSG00000114127	XRN1	5'-3' exoribonuclease 1	783.9417977	2.009771946	0.253186052	3.988260561	6.65595E-05	0.004927049	Cluster 2
ENSG00000241158	ADAMTS9-AS1	ADAMTS9 antisense RNA 1	130.1364007	1.693551855	0.176110248	3.938168635	8.21059E-05	0.006052953	Cluster 2
ENSG00000183535	COL18A1-AS1	COL18A1 antisense RNA 1	142.1293702	-1.606054299	0.154355986	-3.92634141	8.62477E-05	0.006332342	Cluster 1
ENSG00000136603	SKIL	SKI-like proto-oncogene	1262.333198	-1.378885211	0.09669446	-3.918375593	8.91477E-05	0.006518655	Cluster 1
ENSG00000122863	CHST3	carbohydrate (chondroitin 6) sulfotransferase 3	95.92899446	-1.744452937	0.190572473	-3.906403302	9.36801E-05	0.006822339	Cluster 1
ENSG00000145632	PLK2	polo-like kinase 2	1715.598191	1.300402446	0.077548376	3.873742583	0.000107177	0.007766964	Cluster 2
ENSG00000146592	CREB5	cAMP responsive element binding protein 5	71.04256981	-1.839298206	0.21670631	-3.8729754	0.000107515	0.007766964	Cluster 1
ENSG00000165806	CASP7	caspase 7	409.4992691	1.455273399	0.119142525	3.821250214	0.000132777	0.00955356	Cluster 2
ENSG00000221869	CEBPD	CCAAT/enhancer binding protein (C/EBP), delta	22.11461266	2.267965791	0.333211158	3.805292111	0.000141637	0.010150459	Cluster 2
ENSG00000171223	JUNB	jun B proto-oncogene	671.0948894	-1.602648061	0.158802681	-3.794948919	0.000147674	0.010541101	Cluster 1
ENSG00000081479	LRP2	LDL receptor related protein 2	6.154561465	2.401045418	0.370805323	3.778385402	0.000157848	0.011222838	Cluster 2
ENSG00000128594	LRRC4	leucine rich repeat containing 4	49.72652253	-1.929478257	0.248145623	-3.745696766	0.000179894	0.012739879	Cluster 1
ENSG00000177706	FAM20C	family with sequence similarity 20 member C	75.25268866	-1.826271571	0.222816701	-3.708301785	0.000208654	0.014684235	Cluster 1
ENSG00000267598	CTC-250I14.6		516.6607016	-1.477934068	0.128896017	-3.707904093	0.000208982	0.014684235	Cluster 1
ENSG00000112343	TRIM38	tripartite motif containing 38	296.9064196	1.626223824	0.169237026	3.700276707	0.000215365	0.01507384	Cluster 2
ENSG00000279637	RP11-196B3.1		12.99036294	-2.349794421	0.36801246	-3.66779544	0.000244651	0.01705728	Cluster 1
ENSG00000154553	PDLIM3	PDZ and LIM domain 3	77.5322563	-1.79033061	0.216336568	-3.653245574	0.000258946	0.017984275	Cluster 1
ENSG00000205220	PSMB10	proteasome subunit beta 10	140.6755247	1.817094037	0.224477253	3.639985899	0.000272653	0.018863391	Cluster 2
ENSG00000243649	CFB	complement factor B	23.99095462	2.199562421	0.329773064	3.637539118	0.000275255	0.018970475	Cluster 2
ENSG00000124226	RNF114	ring finger protein 114	340.9491206	1.65274108	0.179705438	3.632283408	0.000280924	0.019287279	Cluster 2

ENSG00000120217	CD274	CD274 molecule	108.8300084	1.800637657	0.221550571	3.613791885	0.000301751	0.020638412	Cluster 2
ENSG00000258521	RP11-638I2.9		12.81555778	2.302790038	0.36188481	3.600013046	0.000318201	0.021681074	Cluster 2
ENSG00000006459	KDM7A	lysine (K)-specific demethylase 7A	181.0438746	-1.508410619	0.142455157	-3.568916906	0.00035846	0.024332	Cluster 1
ENSG00000136999	NOV	nephroblastoma overexpressed	19.22890231	2.222875861	0.345327288	3.541208308	0.000398299	0.026934595	Cluster 2
ENSG00000163638	ADAMTS9	ADAM metallopeptidase with thrombospondin type 1 motif 9	1152.3517	1.304282776	0.085964512	3.539632442	0.000400685	0.026994441	Cluster 2
ENSG00000245556	SCAMP1-AS1	SCAMP1 antisense RNA 1	26.68305563	2.079668418	0.306387475	3.523866034	0.000425299	0.02854583	Cluster 2
ENSG00000185880	TRIM69	tripartite motif containing 69	72.21305148	1.82105253	0.233785058	3.511997457	0.000444752	0.029740534	Cluster 2
ENSG00000238160	AC116366.5		13.04409285	2.276493166	0.366514587	3.482789531	0.000496218	0.033059167	Cluster 2
ENSG00000122861	PLAU	plasminogen activator, urokinase	65.68900465	-1.828184906	0.238896403	-3.466711497	0.000526867	0.03497153	Cluster 1
ENSG00000164136	IL15	interleukin 15	38.65632421	2.028156849	0.298145405	3.448508111	0.000563693	0.037278314	Cluster 2
ENSG00000104973	MED25	mediator complex subunit 25	129.8214577	1.583864665	0.169724391	3.440075184	0.000581553	0.038318565	Cluster 2
ENSG00000003402	CFLAR	CASP8 and FADD like apoptosis regulator	1980.295877	1.402639223	0.117094692	3.438577912	0.000584778	0.038390478	Cluster 2
ENSG00000134470	IL15RA	interleukin 15 receptor subunit alpha	171.4786886	1.765708653	0.222899233	3.435223367	0.000592066	0.038727549	Cluster 2
ENSG00000163131	CTSS	cathepsin S	76.58654692	2.031805291	0.300895852	3.429111049	0.000605562	0.039466826	Cluster 2
ENSG00000227051	C14orf132		90.7468605	-1.682512186	0.199891161	-3.41441905	0.000639182	0.041507592	Cluster 1
ENSG00000137727	ARHGAP20	Rho GTPase activating protein 20	40.08260048	1.987609542	0.290723905	3.397070291	0.000681115	0.04407155	Cluster 2
ENSG00000133561	GIMAP6	GTPase, IMAP family member 6	1029.326971	1.282124764	0.083136806	3.393500131	0.000690055	0.044490014	Cluster 2

HUVEC – 16 hpi vs. uninfected

Gene_id	Name	Description	baseMean	log2FoldChange	lfcSE	stat	pvalue	padj	Cluster
ENSG00000119917	IFIT3	interferon induced protein with tetratricopeptide repeats 3	1394.543353	6.547000913	0.274705778	20.19251633	1.13912E-90	3.18488E-86	Cluster 2
ENSG00000101347	SAMHD1	SAM domain and HD domain 1	537.7034743	3.805562608	0.145654246	19.26179759	1.12408E-82	1.5714E-78	Cluster 2
ENSG00000152778	IFIT5	interferon induced protein with tetratricopeptide repeats 5	391.3525165	3.846847186	0.148385404	19.18549336	4.89275E-82	4.55988E-78	Cluster 2
ENSG00000140853	NLRC5	NLR family, CARD domain containing 5	419.1782563	4.497988251	0.189841559	18.42582981	8.15308E-76	5.6988E-72	Cluster 2
ENSG00000128284	APOL3	apolipoprotein L, 3	452.955401	3.863911731	0.15553426	18.41338186	1.0261E-75	5.73777E-72	Cluster 2
ENSG00000263069	CTD-2047H16.4	uncharacterized LOC100294362	415.8456059	4.14520039	0.175477328	17.92368518	7.70507E-72	3.59043E-68	Cluster 2
ENSG00000257452	RP1-71H24.1		107.7353748	5.688673153	0.268083258	17.4896157	1.71913E-68	6.86643E-65	Cluster 2
ENSG00000130589	HELZ2	helicase with zinc finger 2, transcriptional coactivator	2035.697532	4.633212571	0.215350362	16.87117002	7.33294E-64	2.56277E-60	Cluster 2
ENSG00000184979	USP18	ubiquitin specific peptidase 18	334.7952188	3.693348333	0.161690255	16.65745615	2.67148E-62	8.29909E-59	Cluster 2
ENSG00000185885	IFITM1	interferon induced transmembrane protein 1	181.8742945	5.02572448	0.246471733	16.33341247	5.70993E-60	1.59644E-56	Cluster 2
ENSG00000134326	CMPK2	cytidine monophosphate (UMP-CMP) kinase 2, mitochondrial	531.4512502	5.595023796	0.28495207	16.12560245	1.68607E-58	4.28553E-55	Cluster 2
ENSG00000178685	PARP10	poly(ADP-ribose) polymerase family member 10	437.1153032	3.11018954	0.132479342	15.92844218	4.02254E-57	9.37219E-54	Cluster 2
ENSG00000128335	APOL2	apolipoprotein L, 2	631.6611184	3.418414879	0.152661317	15.84170061	1.60423E-56	3.4502E-53	Cluster 2
ENSG00000138496	PARP9	poly(ADP-ribose) polymerase family member 9	512.8227769	3.510568439	0.161778248	15.51857851	2.59741E-54	5.18722E-51	Cluster 2
ENSG00000135899	SP110	SP110 nuclear body protein	217.9349397	4.159140993	0.20365662	15.51209579	2.87345E-54	5.35591E-51	Cluster 2
ENSG00000185507	IRF7	interferon regulatory factor 7	208.6698546	3.603189392	0.170102247	15.30367434	7.22593E-53	1.26269E-49	Cluster 2
ENSG00000173821	RNF213	ring finger protein 213	6329.405111	3.974630234	0.196259848	15.15659094	6.85355E-52	1.12717E-48	Cluster 2
ENSG00000170581	STAT2	signal transducer and activator of transcription 2	901.7025303	3.332548535	0.154467719	15.10055659	1.60572E-51	2.49413E-48	Cluster 2
ENSG00000156587	UBE2L6	ubiquitin conjugating enzyme E2L 6	248.47387	3.357910277	0.156406022	15.07557216	2.34476E-51	3.45037E-48	Cluster 2
ENSG00000068079	IFI35	interferon induced protein 35	274.7578354	4.251304185	0.219961437	14.78124635	1.93551E-49	2.70575E-46	Cluster 2
ENSG00000280248	CTD-2047H16.3		224.3860078	3.932915806	0.200884989	14.59997496	2.80949E-48	3.7405E-45	Cluster 2
ENSG00000130303	BST2	bone marrow stromal cell antigen 2	397.1495681	3.384344717	0.165384086	14.41701418	4.04465E-47	5.1402E-44	Cluster 2
ENSG00000060491	OGFR	opioid growth factor receptor	426.9608299	2.458859338	0.101678971	14.34769965	1.1013E-46	1.33875E-43	Cluster 2
ENSG00000168961	LGALS9	lectin, galactoside-binding, soluble, 9	261.3994593	3.215966722	0.154582941	14.33513104	1.31997E-46	1.53771E-43	Cluster 2
ENSG00000089127	OAS1	2'-5'-oligoadenylate synthetase 1	432.480613	5.153729432	0.293495799	14.1526027	1.79971E-45	2.01272E-42	Cluster 2
ENSG00000119922	IFIT2	interferon induced protein with tetratricopeptide repeats 2	920.0274502	5.691911595	0.333732119	14.05891531	6.79192E-45	7.30367E-42	Cluster 2
ENSG00000168062	BATF2	basic leucine zipper transcription factor, ATF-like 2	83.01637811	5.329334951	0.308320849	14.04165486	8.6665E-45	8.97432E-42	Cluster 2
ENSG00000225886	RP11-288L9.4		62.80870824	5.418559483	0.317039936	13.93691766	3.77912E-44	3.77359E-41	Cluster 2
ENSG00000172183	ISG20	interferon stimulated exonuclease gene 20kDa	181.6549416	4.302988988	0.237647987	13.89866173	6.45368E-44	6.22201E-41	Cluster 2
ENSG00000115267	IFIH1	interferon induced, with helicase C domain 1	628.4811053	4.941261144	0.28488189	13.83471988	1.57348E-43	1.46643E-40	Cluster 2
ENSG00000130813	C19orf66	chromosome 19 open reading frame 66	348.5192544	3.246239388	0.163204076	13.763378	4.23278E-43	3.81756E-40	Cluster 2
ENSG00000168016	TRANK1	tetratricopeptide repeat and ankyrin repeat containing 1	293.1633185	3.644369508	0.193700015	13.65188075	1.96735E-42	1.71891E-39	Cluster 2
ENSG00000276863	CTD-2047H16.5		147.6465321	3.825457551	0.20755891	13.61279819	3.36124E-42	2.84778E-39	Cluster 2
ENSG00000121060	TRIM25	tripartite motif containing 25	866.691858	2.359066056	0.101099913	13.44280148	3.39342E-41	2.79049E-38	Cluster 2
ENSG00000183486	MX2	MX dynamin-like GTPase 2	356.0366428	5.743940577	0.353777182	13.40940234	5.32663E-41	4.25507E-38	Cluster 2
ENSG00000221963	APOL6	apolipoprotein L, 6	631.5877576	5.116780787	0.307755964	13.37677012	8.26621E-41	6.41986E-38	Cluster 2
ENSG00000138646	HERC5	HECT and RLD domain containing E3 ubiquitin protein ligase 5	376.7845114	5.142285533	0.310907846	13.32319396	1.6969E-40	1.28226E-37	Cluster 2
ENSG00000132109	TRIM21	tripartite motif containing 21	164.9208847	3.202489663	0.166134096	13.25730069	4.09367E-40	3.01197E-37	Cluster 2
ENSG00000280755	AC009950.1	SP110 nuclear body protein	150.438364	3.906800652	0.222068711	13.08964526	3.77366E-39	2.70533E-36	Cluster 2

ENSG00000187608	ISG15	ISG15 ubiquitin-like modifier	1434.920784	4.239611276	0.252296967	12.84046857	9.72839E-38	6.7999E-35	Cluster 2
ENSG00000111331	OAS3	2'-5'-oligoadenylate synthetase 3	940.0457269	4.500241229	0.273193491	12.8123156	1.39897E-37	9.53992E-35	Cluster 2
ENSG00000115415	STAT1	signal transducer and activator of transcription 1	2768.269437	2.797654524	0.142791539	12.58936304	2.41615E-36	1.60841E-33	Cluster 2
ENSG00000205413	SAMD9	sterile alpha motif domain containing 9	657.1221937	4.103889808	0.247570321	12.53740671	4.65997E-36	3.02995E-33	Cluster 2
ENSG00000138642	HERC6	HECT and RLD domain containing E3 ubiquitin protein ligase family member 6	457.1481929	4.255635496	0.262708007	12.39260097	2.86595E-35	1.82111E-32	Cluster 2
ENSG00000107201	DDX58	DEAD (Asp-Glu-Ala-Asp) box polypeptide 58	1210.566667	4.281727968	0.270794947	12.1188671	8.3911E-34	5.21348E-31	Cluster 2
ENSG00000100342	APOL1	apolipoprotein L1	222.1961015	3.548827878	0.212710772	11.98259899	4.38362E-33	2.66438E-30	Cluster 2
ENSG00000124201	ZNFX1	zinc finger, NFX1-type containing 1	962.249264	2.774497611	0.148787927	11.9263548	8.6269E-33	5.13191E-30	Cluster 2
ENSG00000188313	PLSCR1	phospholipid scramblase 1	465.2297432	2.672721646	0.140451643	11.9095912	1.05493E-32	6.14476E-30	Cluster 2
ENSG00000181381	DDX60L	DEAD (Asp-Glu-Ala-Asp) box polypeptide 60-like	1813.790747	3.44499055	0.207762503	11.76819933	5.69266E-32	3.24819E-29	Cluster 2
ENSG00000112773	FAM46A	family with sequence similarity 46 member A	73.06726952	3.84592778	0.242547847	11.73346956	8.58654E-32	4.80142E-29	Cluster 2
ENSG00000101342	TLDC2	TBC/LysM-associated domain containing 2	65.56340199	3.693790368	0.23033387	11.69515524	1.3494E-31	7.39763E-29	Cluster 2
ENSG00000267387	CTD-2240E14.4		124.3228533	3.280067605	0.195420719	11.66748138	1.86874E-31	1.00477E-28	Cluster 2
ENSG00000182179	UBA7	ubiquitin-like modifier activating enzyme 7	287.0727325	2.753797456	0.151006929	11.61401977	3.49777E-31	1.84517E-28	Cluster 2
ENSG00000173193	PARP14	poly(ADP-ribose) polymerase family member 14	3230.511171	3.61203406	0.228295354	11.44146831	2.59457E-30	1.34336E-27	Cluster 2
ENSG00000185745	IFIT1	interferon induced protein with tetratricopeptide repeats 1	1399.14554	5.150021688	0.368238448	11.26993043	1.84716E-29	9.38994E-27	Cluster 2
ENSG00000240065	PSMB9	proteasome subunit beta 9	68.55825235	4.026705804	0.268672979	11.26538968	1.9449E-29	9.71024E-27	Cluster 2
ENSG00000204261	PSMB8-AS1	PSMB8 antisense RNA 1 (head to head)	156.4121888	3.663347729	0.240998061	11.05132431	2.16005E-28	1.05952E-25	Cluster 2
ENSG00000131979	GCH1	GTP cyclohydrolase 1	143.3497883	3.780674806	0.251721238	11.0466436	2.27565E-28	1.09698E-25	Cluster 2
ENSG00000225963	AC009950.2		45.38416995	4.103208185	0.282722613	10.9761584	4.97639E-28	2.35822E-25	Cluster 2
ENSG00000111335	OAS2	2'-5'-oligoadenylate synthetase 2	764.0131342	4.803160748	0.347937375	10.93058986	8.23116E-28	3.83558E-25	Cluster 2
ENSG00000002549	LAP3	leucine aminopeptidase 3	1368.79701	2.222782495	0.113816399	10.74346495	6.36112E-27	2.91558E-24	Cluster 2
ENSG00000132530	XAF1	XIAP associated factor 1	332.0599535	3.851816844	0.266613917	10.69642902	1.05776E-26	4.77E-24	Cluster 2
ENSG00000134321	RSAD2	radical S-adenosyl methionine domain containing 2	1354.259541	4.914605814	0.37499497	10.43908886	1.64381E-25	7.29512E-23	Cluster 2
ENSG00000108771	DHX58	DEXH (Asp-Glu-X-His) box polypeptide 58	123.4589312	3.559110577	0.245701859	10.41551168	2.10662E-25	9.20295E-23	Cluster 2
ENSG00000168394	TAP1	transporter 1, ATP-binding cassette, sub-family B (MDR/TAP)	688.4815401	3.854651403	0.275386536	10.36598028	3.54109E-25	1.52316E-22	Cluster 2
ENSG00000157601	MX1	MX dynamin-like GTPase 1	3295.228182	4.617466262	0.351430371	10.29355048	7.53458E-25	3.19181E-22	Cluster 2
ENSG00000136514	RTP4	receptor (chemosensory) transporter protein 4	29.15871251	4.539720648	0.347368534	10.19010157	2.19535E-24	9.12567E-22	Cluster 2
ENSG00000160932	LY6E	lymphocyte antigen 6 complex, locus E	332.6783787	2.46125838	0.143414747	10.18903852	2.21948E-24	9.12567E-22	Cluster 2
ENSG00000204267	TAP2	transporter 2, ATP-binding cassette, sub-family B (MDR/TAP)	390.8166902	2.627344425	0.162021487	10.04400374	9.76292E-24	3.95596E-21	Cluster 2
ENSG00000177409	SAMD9L	sterile alpha motif domain containing 9-like	805.651111	3.524771047	0.251960749	10.02049351	1.23883E-23	4.94806E-21	Cluster 2
ENSG00000135114	OASL	2'-5'-oligoadenylate synthetase-like	1123.835101	4.764715451	0.377750594	9.966140388	2.14399E-23	8.44279E-21	Cluster 2
ENSG00000163644	PPM1K	protein phosphatase, Mg2+/Mn2+ dependent 1K	290.6755824	2.725650579	0.17319151	9.963828931	2.19445E-23	8.52146E-21	Cluster 2
ENSG00000228318	AP001610.5		733.0868016	4.497639209	0.352703525	9.916655095	3.52367E-23	1.34957E-20	Cluster 2
ENSG00000067066	SP100	SP100 nuclear antigen	803.3625513	2.137421965	0.115495565	9.848187339	6.97913E-23	2.63688E-20	Cluster 2
ENSG00000131203	IDO1	indoleamine 2,3-dioxygenase 1	48.17855099	4.593009048	0.366435454	9.805298607	1.06831E-22	3.9825E-20	Cluster 2
ENSG00000137965	IFI44	interferon induced protein 44	777.1401967	3.872849012	0.297106838	9.66941397	4.067E-22	1.49618E-19	Cluster 2
ENSG00000250264	XXbac-BPG246D15.9		251.6781398	2.572244665	0.162780351	9.658688259	4.51609E-22	1.63981E-19	Cluster 2
ENSG00000204264	PSMB8	proteasome subunit beta 8	209.3800008	2.345540556	0.139572868	9.64041633	5.39686E-22	1.9345E-19	Cluster 2
ENSG00000138035	PNPT1	polyribonucleotide nucleotidyltransferase 1	574.7822674	2.317153267	0.142076308	9.270745341	1.8485E-20	6.54204E-18	Cluster 2
ENSG00000141574	SECTM1	secreted and transmembrane 1	47.04872313	4.404762402	0.368929994	9.228749231	2.7381E-20	9.56933E-18	Cluster 2

ENSG00000137628	DDX60	DEAD (Asp-Glu-Ala-Asp) box polypeptide 60	311.1276007	2.75425497	0.192057799	9.133994991	6.60171E-20	2.27873E-17	Cluster 2
ENSG00000234745	HLA-B	major histocompatibility complex, class I, B	781.498232	2.525667201	0.167148271	9.127627779	7.00166E-20	2.38731E-17	Cluster 2
ENSG00000130812	ANGPTL6	angiopoietin like 6	96.75376562	3.047094947	0.224483281	9.119142155	7.57213E-20	2.55071E-17	Cluster 2
ENSG00000138670	RASGEF1B	RasGEF domain family member 1B	47.05669694	4.073050858	0.338668598	9.073917319	1.14813E-19	3.8215E-17	Cluster 2
ENSG00000197536	C5orf56		111.5178767	2.914719703	0.215580241	8.881703131	6.58444E-19	2.16581E-16	Cluster 2
ENSG00000133106	EPSTI1	epithelial stromal interaction 1 (breast)	437.8594816	4.277858726	0.370242765	8.853268813	8.49926E-19	2.76315E-16	Cluster 2
ENSG00000146859	TMEM140	transmembrane protein 140	194.14462	2.281589176	0.145152489	8.829260745	1.0537E-18	3.38625E-16	Cluster 2
ENSG00000137198	GMPR	guanosine monophosphate reductase	193.6646801	2.747127741	0.198471844	8.802899723	1.33326E-18	4.23448E-16	Cluster 2
ENSG00000059378	PARP12	poly(ADP-ribose) polymerase family member 12	571.0561791	2.029355107	0.116949958	8.801671487	1.34793E-18	4.23448E-16	Cluster 2
ENSG00000173786	CNP	2',3'-cyclic nucleotide 3' phosphodiesterase	1024.102411	2.046738033	0.118997532	8.796300353	1.41401E-18	4.39269E-16	Cluster 2
ENSG00000132274	TRIM22	tripartite motif containing 22	1347.27451	2.253136445	0.143583168	8.727599902	2.60134E-18	7.9924E-16	Cluster 2
ENSG00000110446	SLC15A3	solute carrier family 15 (oligopeptide transporter), member 3	98.8390244	3.014941956	0.231402673	8.707513756	3.10613E-18	9.43958E-16	Cluster 2
ENSG00000125347	IRF1	interferon regulatory factor 1	344.1413453	2.69454559	0.200580364	8.448212755	2.95796E-17	8.89264E-15	Cluster 2
ENSG00000155287	SLC25A28	solute carrier family 25 (mitochondrial iron transporter), member 28	155.0327851	2.644007791	0.198433251	8.284941089	1.18168E-16	3.51473E-14	Cluster 2
ENSG00000055332	EIF2AK2	eukaryotic translation initiation factor 2 alpha kinase 2	954.9186499	2.223100046	0.147982213	8.265182807	1.39481E-16	4.10499E-14	Cluster 2
ENSG00000096696	DSP	desmoplakin	70.84729299	3.045009658	0.248640374	8.224769067	1.95567E-16	5.69569E-14	Cluster 2
ENSG00000169248	CXCL11	chemokine (C-X-C motif) ligand 11	699.2194082	4.037573252	0.37005652	8.208403554	2.24149E-16	6.4608E-14	Cluster 2
ENSG00000172936	MYD88	myeloid differentiation primary response 88	222.8247188	2.181225594	0.14538912	8.1245804	4.48913E-16	1.28073E-13	Cluster 2
ENSG00000266728	AC015688.3		106.8314222	2.424856259	0.17665053	8.065960855	7.26621E-16	2.05208E-13	Cluster 2
ENSG00000126709	IFI6	interferon, alpha-inducible protein 6	835.6537911	3.584598941	0.323937877	7.978687048	1.47898E-15	4.13508E-13	Cluster 2
ENSG00000140464	PML	promyelocytic leukemia	418.6767923	2.253665985	0.157546665	7.957426382	1.75655E-15	4.8625E-13	Cluster 2
ENSG00000225864	HCG4P11		21.15026919	3.825829673	0.357738468	7.899149599	2.80813E-15	7.6973E-13	Cluster 2
ENSG00000234290	AC116366.6	uncharacterized LOC105379175	47.52537818	3.000835743	0.2550995	7.843354217	4.38668E-15	1.19075E-12	Cluster 2
ENSG00000163840	DTX3L	deltex 3 like, E3 ubiquitin ligase	587.8976362	2.92900973	0.248113086	7.774719839	7.56146E-15	2.0328E-12	Cluster 2
ENSG00000162654	GBP4	guanylate binding protein 4	1341.692457	3.572234676	0.335821999	7.659518083	1.86632E-14	4.96957E-12	Cluster 2
ENSG00000204525	HLA-C	major histocompatibility complex, class I, C	772.0531872	2.126562673	0.14717166	7.654752795	1.93684E-14	5.1087E-12	Cluster 2
ENSG00000117226	GBP3	guanylate binding protein 3	159.3868754	2.386669082	0.181746364	7.629693662	2.35312E-14	6.14869E-12	Cluster 2
ENSG00000123609	NMI	N-myc and STAT interactor	137.4867522	2.418374682	0.187280237	7.573541681	3.63184E-14	9.4021E-12	Cluster 2
ENSG00000137959	IFI44L	interferon induced protein 44 like	1784.087937	3.830844821	0.375985313	7.529136712	5.10769E-14	1.31015E-11	Cluster 2
ENSG00000106785	TRIM14	tripartite motif containing 14	212.908226	2.017230819	0.13528196	7.519338269	5.50542E-14	1.39933E-11	Cluster 2
ENSG00000121858	TNFSF10	tumor necrosis factor superfamily member 10	843.7576164	3.311906146	0.310295689	7.450655057	9.28779E-14	2.33944E-11	Cluster 2
ENSG00000166750	SLFN5	schlafen family member 5	671.1521348	2.170559995	0.157276877	7.442670628	9.86699E-14	2.46314E-11	Cluster 2
ENSG00000271503	CCL5	chemokine (C-C motif) ligand 5	24.53621412	3.785326909	0.374663266	7.434214023	1.05192E-13	2.6027E-11	Cluster 2
ENSG00000136826	KLF4	Kruppel-like factor 4 (gut)	178.2964331	2.73696114	0.235647396	7.371017765	1.6933E-13	4.11679E-11	Cluster 1
ENSG00000262112	RP11-670E13.5		133.0556563	2.177270958	0.159697694	7.371871988	1.68249E-13	4.11679E-11	Cluster 2
ENSG00000271581	XXbac-BPG248L24.12		230.4336802	2.504511005	0.208296074	7.22294461	5.08737E-13	1.22619E-10	Cluster 2
ENSG00000164342	TLR3	toll-like receptor 3	99.52457431	3.51990219	0.350542009	7.18858832	6.54646E-13	1.56438E-10	Cluster 2
ENSG00000168404	MLKL	mixed lineage kinase domain-like	174.1473205	2.432793566	0.199902823	7.167450378	7.64073E-13	1.8104E-10	#N/A
ENSG00000204642	HLA-F	major histocompatibility complex, class I, F	64.58755191	2.900491027	0.266764965	7.124215237	1.04675E-12	2.45934E-10	Cluster 2
ENSG00000185201	IFITM2	interferon induced transmembrane protein 2	207.0203681	2.405574084	0.198629336	7.076367025	1.47983E-12	3.44787E-10	Cluster 2
ENSG00000279296	RP11-609D21.3		137.8440574	3.313674417	0.327361341	7.067647061	1.57583E-12	3.6412E-10	Cluster 2

ENSG00000117228	GBP1	guanylate binding protein 1, interferon-inducible	579.6687074	3.165621321	0.307513463	7.042362625	1.89007E-12	4.33152E-10	Cluster 2
ENSG00000010030	ETV7	ets variant 7	25.25225197	3.518013537	0.363937451	6.918808529	4.55458E-12	1.0353E-09	Cluster 2
ENSG00000132470	ITGB4	integrin beta 4	90.12607484	2.344864776	0.19470679	6.907128298	4.94563E-12	1.11512E-09	Cluster 2
ENSG00000239713	APOBEC3G	apolipoprotein B mRNA editing enzyme, catalytic polypeptide-like 3G	46.97332891	3.143784858	0.311545237	6.881135069	5.93775E-12	1.32811E-09	Cluster 2
ENSG00000238025	ZDHHC4P1		13.81069942	3.535067971	0.372157673	6.811811638	9.63772E-12	2.13858E-09	Cluster 2
ENSG00000105939	ZC3HAV1	zinc finger CCCH-type, antiviral 1	697.5668898	2.096694901	0.161673402	6.783397173	1.17382E-11	2.58417E-09	Cluster 2
ENSG00000127528	KLF2	Kruppel-like factor 2	179.5183644	2.06037386	0.156483988	6.776245131	1.23339E-11	2.6941E-09	Cluster 1
ENSG00000141682	PMAIP1	phorbol-12-myristate-13-acetate-induced protein 1	259.8845716	2.893724219	0.279630112	6.772247118	1.26797E-11	2.74816E-09	Cluster 2
ENSG00000043143	JADE2	jade family PHD finger 2	259.4342307	2.02447789	0.152641363	6.711666298	1.92414E-11	4.13824E-09	Cluster 2
ENSG00000154451	GBP5	guanylate binding protein 5	21.35526702	3.410503846	0.366888777	6.570121517	5.02742E-11	1.07299E-08	Cluster 2
ENSG00000185880	TRIM69	tripartite motif containing 69	72.21305148	2.511009794	0.231060624	6.539451715	6.17448E-11	1.30782E-08	Cluster 2
ENSG00000196116	TDRD7	tudor domain containing 7	175.0094745	2.206642675	0.18698409	6.453183651	1.09524E-10	2.3024E-08	Cluster 2
ENSG00000171388	APLN	apelin	896.8443711	-1.550046759	0.085803723	-6.410523237	1.45021E-10	3.02585E-08	#N/A
ENSG00000160710	ADAR	adenosine deaminase, RNA-specific	1726.799075	1.731164846	0.11744458	6.225615912	4.79668E-10	9.9341E-08	Cluster 2
ENSG00000184371	CSF1	colony stimulating factor 1 (macrophage)	92.01746268	2.564798905	0.253052442	6.183694141	6.26186E-10	1.28732E-07	#N/A
ENSG00000110057	UNC93B1	unc-93 homolog B1 (C. elegans)	140.832682	2.204782508	0.19598763	6.147237493	7.8844E-10	1.60905E-07	Cluster 2
ENSG00000142961	MOB3C	MOB kinase activator 3C	108.2317261	2.009437392	0.16450413	6.136243478	8.44956E-10	1.71189E-07	Cluster 2
ENSG00000137752	CASP1	caspase 1	44.39741738	2.669900423	0.279963783	5.964701606	2.45081E-09	4.92966E-07	Cluster 2
ENSG00000105559	PLEKHA4	pleckstrin homology domain containing A4	71.82932436	2.49351368	0.25053388	5.961324195	2.50202E-09	4.99671E-07	Cluster 2
ENSG00000269720	CTD-2521M24.5		9.880737249	3.229671819	0.3779495	5.89939085	3.64846E-09	7.23456E-07	Cluster 2
ENSG00000155363	MOV10	Mov10 RISC complex RNA helicase	385.1859822	1.808332536	0.139710636	5.785762352	7.21842E-09	1.42127E-06	Cluster 2
ENSG00000102921	N4BP1	NEDD4 binding protein 1	195.0511851	2.034134201	0.182226064	5.675007065	1.38683E-08	2.71149E-06	Cluster 2
ENSG00000136147	PHF11	PHD finger protein 11	103.3452573	1.919486265	0.163969919	5.607652142	2.0509E-08	3.98202E-06	Cluster 2
ENSG00000130775	THEMIS2	thymocyte selection associated family member 2	60.86618117	2.488719572	0.266334422	5.589662651	2.27511E-08	4.38689E-06	Cluster 2
ENSG00000173530	TNFRSF10D	tumor necrosis factor receptor superfamily member 10d, decoy with truncated death domain	793.7748272	-1.609384587	0.110193748	-5.530119409	3.20013E-08	6.12825E-06	Cluster 1
ENSG00000133321	RARRES3	retinoic acid receptor responder (tazarotene induced) 3	15.1564096	3.013248888	0.365994423	5.500763843	3.78149E-08	7.1437E-06	Cluster 2
ENSG00000132622	HSPA12B	heat shock protein family A (Hsp70) member 12B	58.41989996	2.213463076	0.220580236	5.501232104	3.77146E-08	7.1437E-06	Cluster 2
ENSG00000141664	ZCCHC2	zinc finger, CCHC domain containing 2	239.2721298	1.670971764	0.126766835	5.292959834	1.20352E-07	2.25834E-05	#N/A
ENSG00000163565	IFI16	interferon, gamma-inducible protein 16	951.145506	1.716786308	0.136043872	5.268787914	1.37328E-07	2.55969E-05	Cluster 2
ENSG00000004468	CD38	CD38 molecule	25.14643705	2.949495351	0.376379914	5.179594543	2.22369E-07	4.11736E-05	Cluster 2
ENSG00000026950	BTN3A1	butyrophilin subfamily 3 member A1	52.0139792	2.306454713	0.252783432	5.168276673	2.36262E-07	4.34583E-05	#N/A
ENSG00000169871	TRIM56	tripartite motif containing 56	225.7994339	1.673925418	0.130695038	5.156472874	2.51645E-07	4.59852E-05	Cluster 2
ENSG00000137842	TMEM62	transmembrane protein 62	140.7285858	1.839427583	0.163621487	5.130301633	2.89278E-07	5.2519E-05	Cluster 2
ENSG00000196776	CD47	CD47 molecule	483.8831496	1.783372932	0.153984222	5.087358439	3.63085E-07	6.54935E-05	Cluster 2
ENSG00000132256	TRIM5	tripartite motif containing 5	458.7526789	1.709864713	0.141738533	5.008269068	5.49217E-07	9.84331E-05	#N/A
ENSG00000225075	RP11-426L16.3		41.56134499	2.257167674	0.251120757	5.006227636	5.55071E-07	9.88486E-05	Cluster 2
ENSG00000023445	BIRC3	baculoviral IAP repeat containing 3	40.03472066	2.531406923	0.31097974	4.924458812	8.45941E-07	0.000149694	#N/A
ENSG00000198719	DLL1	delta-like 1 (Drosophila)	71.73402048	2.091174907	0.222375953	4.906892543	9.25307E-07	0.000162709	#N/A
ENSG00000001629	ANKIB1	ankyrin repeat and IBR domain containing 1	315.2387528	1.602923343	0.124067457	4.859641344	1.17599E-06	0.000205496	#N/A
ENSG00000125826	RBCK1	RanBP-type and C3HC4-type zinc finger containing 1	324.2751346	1.681619243	0.142119663	4.79609387	1.61789E-06	0.000280961	#N/A
ENSG00000188290	HES4	hes family bHLH transcription factor 4	163.6132349	1.880136242	0.184251769	4.776812986	1.78095E-06	0.000307368	#N/A

ENSG00000120690	ELF1	E74-like factor 1 (ets domain transcription factor)	402.8167686	1.711015999	0.154843838	4.591826246	4.39384E-06	0.000753665	#N/A
ENSG00000225492	GBP1P1	guanylate binding protein 1, interferon-inducible pseudogene 1	10.48246095	2.734645854	0.379000413	4.576897008	4.71924E-06	0.000804543	Cluster 2
ENSG00000185722	ANKFY1	ankyrin repeat and FYVE domain containing 1	383.6505267	1.569854762	0.12555744	4.538598143	5.66294E-06	0.000959577	Cluster 2
ENSG00000116514	RNF19B	ring finger protein 19B	209.5293317	1.707470511	0.157429288	4.493893877	6.99325E-06	0.001177857	#N/A
ENSG00000116663	FBXO6	F-box protein 6	25.85490339	2.353544609	0.30523457	4.434440727	9.23116E-06	0.001540695	#N/A
ENSG00000216490	IFI30	interferon, gamma-inducible protein 30	19.5700805	2.667444566	0.3760739	4.433821566	9.25773E-06	0.001540695	Cluster 2
ENSG00000100226	GTPBP1	GTP binding protein 1	569.5488583	1.589102902	0.133271921	4.420307732	9.85604E-06	0.001630563	#N/A
ENSG00000137727	ARHGAP20	Rho GTPase activating protein 20	40.08260048	2.220291739	0.280835	4.345226698	1.39132E-05	0.002288229	Cluster 2
ENSG00000081479	LRP2	LDL receptor related protein 2	6.154561465	2.630946444	0.376503798	4.331819361	1.47882E-05	0.002417918	Cluster 2
ENSG00000258521	RP11-638I2.9		12.81555778	2.52994973	0.353694497	4.325624915	1.521E-05	0.002472422	Cluster 2
ENSG00000137200	CMTR1	cap methyltransferase 1	332.8999316	1.548790337	0.126932446	4.323483508	1.53585E-05	0.002482123	Cluster 2
ENSG00000112343	TRIM38	tripartite motif containing 38	296.9064196	1.707879716	0.164763181	4.296346511	1.73636E-05	0.002790053	Cluster 2
ENSG00000174640	SLCO2A1	solute carrier organic anion transporter family member 2A1	24.43114885	2.208370382	0.282160434	4.282564939	1.84751E-05	0.002951691	Cluster 2
ENSG00000165949	IFI27	interferon, alpha-inducible protein 27	519.7449245	2.277315305	0.30316389	4.213283137	2.51685E-05	0.00399822	Cluster 2
ENSG00000135148	TRAFD1	TRAF-type zinc finger domain containing 1	296.9174084	1.831258852	0.19889292	4.179429072	2.92242E-05	0.004616265	Cluster 2
ENSG00000079385	CEACAM1	carcinoembryonic antigen-related cell adhesion molecule 1 (biliary glycoprotein)	26.00048596	2.414511483	0.341332497	4.14408677	3.41171E-05	0.005358869	#N/A
ENSG00000213928	IRF9	interferon regulatory factor 9	148.5322803	1.802457415	0.193700868	4.142766235	3.43142E-05	0.005359719	Cluster 2
ENSG00000117475	BLZF1	basic leucine zipper nuclear factor 1	162.1984777	1.748579582	0.18080914	4.140164495	3.47057E-05	0.005390757	#N/A
ENSG00000244255	XXbac-BPG116M5.17		22.67599839	2.323968362	0.323345844	4.094589078	4.22918E-05	0.006532795	Cluster 2
ENSG00000124226	RNF114	ring finger protein 114	340.9491206	1.717070694	0.175971897	4.07491597	4.6031E-05	0.007071317	Cluster 2
ENSG00000004799	PDK4	pyruvate dehydrogenase kinase, isozyme 4	119.7633771	2.070850828	0.264378484	4.050446214	5.11201E-05	0.007810195	Cluster 2
ENSG00000072121	ZFYVE26	zinc finger, FYVE domain containing 26	501.7855797	1.602879449	0.149202653	4.040675126	5.32975E-05	0.00809862	#N/A
ENSG00000243649	CFB	complement factor B	23.99095462	2.279124695	0.317159223	4.033067944	5.50534E-05	0.008320198	Cluster 2
ENSG00000162645	GBP2	guanylate binding protein 2, interferon-inducible	486.2106326	1.731982055	0.185544497	3.945048571	7.97838E-05	0.011992878	#N/A
ENSG00000196141	SPATS2L	spermatogenesis associated, serine rich 2 like	344.1209706	1.559745238	0.141938004	3.943589602	8.0271E-05	0.012001585	#N/A
ENSG00000277402	MIR6891	microRNA 6891	15.84701502	2.332245446	0.338922856	3.930822077	8.46559E-05	0.012589864	Cluster 2
ENSG00000005810	MYCBP2	MYC binding protein 2, E3 ubiquitin protein ligase	1343.230927	1.591393027	0.150585031	3.927302887	8.59038E-05	0.01270785	#N/A
ENSG00000038210	PI4K2B	phosphatidylinositol 4-kinase type 2 beta	320.3848889	1.698160123	0.178298336	3.915685024	9.01479E-05	0.013265497	Cluster 2
ENSG00000134470	IL15RA	interleukin 15 receptor subunit alpha	171.4786886	1.845822163	0.217635185	3.886421959	0.000101733	0.014891843	Cluster 2
ENSG00000224611	AC007919.18		10.17385468	2.420187628	0.370544092	3.832708875	0.00012674	0.018455839	Cluster 2
ENSG00000258581	RP11-638I2.10		12.52613757	2.427907421	0.375165181	3.806076615	0.000141189	0.020453336	#N/A
ENSG00000144837	PLA1A	phospholipase A1 member A	12.28602337	2.440711549	0.378791775	3.803439363	0.000142701	0.020565853	#N/A
ENSG00000277511	CTD-2095E4.5		9.434067971	2.400897752	0.369473677	3.791603679	0.000149678	0.021460703	Cluster 2
ENSG00000140105	WARS	tryptophanyl-tRNA synthetase	2285.567922	2.142261146	0.307413887	3.71571095	0.000202633	0.028905202	#N/A
ENSG00000114127	XRN1	5'-3' exoribonuclease 1	783.9417977	1.917122937	0.2519542	3.640038292	0.000272597	0.038688089	Cluster 2
ENSG00000096968	JAK2	Janus kinase 2	269.597428	2.092478962	0.302963143	3.605979762	0.000310977	0.043912204	#N/A
ENSG00000102524	TNFSF13B	tumor necrosis factor superfamily member 13b	35.23610173	2.360508331	0.377815494	3.600986068	0.000317013	0.044539469	#N/A

Supplementary Table S3

Two tables containing the HUVEC genes from Cluster 1 and 2 from the functional enrichment analysis that have been mapped to the Reactome Pathway database.

HUVEC Cluster 1

Cluster	ID	Description	GeneRatio	BgRatio	pvalue	p.adjust	qvalue	geneID	Count
Cluster 1	R-HSA-9006936	Signaling by TGF-beta family members	5/45	102/10619	6.67311E-05	0.013234824	0.010745291	6498/4091/9241/4092/3726	5
Cluster 1	R-HSA-112409	RAF-independent MAPK1/3 activation	3/45	23/10619	0.000118698	0.013234824	0.010745291	1844/1843/1847	3
Cluster 1	R-HSA-201451	Signaling by BMP	3/45	28/10619	0.000216338	0.016081155	0.013056213	4091/9241/4092	3
Cluster 1	R-HSA-5675221	Negative regulation of MAPK pathway	3/45	40/10619	0.000629679	0.035104632	0.028501282	1844/1843/1847	3
Cluster 1	R-HSA-2173793	Transcriptional activity of SMAD2/SMAD3:SMAD4 heterotrimer	3/45	44/10619	0.000834151	0.037203142	0.030205053	6498/4092/3726	3

HUVEC Cluster 2

Cluster	ID	Description	GeneRatio	BgRatio	pvalue	p.adjust	qvalue	geneID	Count
Cluster 2	R-HSA-913531	Interferon Signaling	50/118	199/10619	1.3866E-56	5.07494E-54	3.70732E-54	9636/2537/2635/2633/115361/115362/103/91543/5610/6772/6672/7318/51191/3659/10475/3134/3133/3107/3106/5696/23586/9830/10581/8519/10410/3665/6737/10346/9246/3433/3437/3434/6773/4938/4940/4939/8638/10379/3429/5371/3669/54739/3430/7706/25939/684/10437/11274/4600/4599	50
Cluster 2	R-HSA-909733	Interferon alpha/beta signaling	33/118	69/10619	9.62277E-48	1.76097E-45	1.28641E-45	9636/2537/103/91543/6772/3659/3134/3133/3107/3106/5696/10581/8519/10410/3665/3433/3437/3434/6773/4938/4940/4939/8638/10379/3429/3669/54739/3430/25939/684/11274/4600/4599	33
Cluster 2	R-HSA-877300	Interferon gamma signaling	24/118	92/10619	4.71974E-27	5.75808E-25	4.20637E-25	2635/2633/115361/115362/6772/6672/3659/10475/3134/3133/3107/3106/9830/3665/6737/10346/4938/4940/4939/8638/10379/5371/7706/10437	24
Cluster 2	R-HSA-1169410	Antiviral mechanism by IFN-stimulated genes	16/118	80/10619	2.83628E-16	2.59519E-14	1.89583E-14	9636/5610/6772/7318/51191/23586/9246/3434/4938/4940/4939/8638/7706/11274/4600/4599	16
Cluster 2	R-HSA-1169408	ISG15 antiviral mechanism	12/118	72/10619	1.76136E-11	1.28931E-09	9.41863E-10	9636/5610/6772/7318/51191/23586/9246/3434/7706/11274/4600/4599	12
Cluster 2	R-HSA-936440	Negative regulators of DDX58/IFIH1 signaling	8/118	34/10619	2.61072E-09	1.59254E-07	1.16338E-07	9636/64135/7318/51191/23586/9246/84166/7706	8
Cluster 2	R-HSA-983169	Class I MHC mediated antigen processing & presentation	20/118	371/10619	3.91588E-09	2.04745E-07	1.49569E-07	1520/4615/7318/151636/55008/51191/3134/3133/3107/3106/6891/5696/5698/6890/6737/9246/140691/5699/57674/55905	20
Cluster 2	R-HSA-1236975	Antigen processing-Cross presentation	11/118	99/10619	1.10296E-08	5.04606E-07	3.68622E-07	1520/4615/3134/3133/3107/3106/6891/5696/5698/6890/5699	11
Cluster 2	R-HSA-168928	DDX58/IFIH1-mediated induction of interferon-alpha/beta	10/118	78/10619	1.30236E-08	5.29625E-07	3.86899E-07	9636/64135/7318/51191/23586/3665/9246/84166/79132/7706	10
Cluster 2	R-HSA-1236974	ER-Phagosome pathway	10/118	83/10619	2.40308E-08	8.79527E-07	6.42507E-07	4615/3134/3133/3107/3106/6891/5696/5698/6890/5699	10
Cluster 2	R-HSA-1236977	Endosomal/Vacuolar pathway	5/118	11/10619	6.81633E-08	2.26798E-06	1.65679E-06	1520/3134/3133/3107/3106	5
Cluster 2	R-HSA-983170	Antigen Presentation: Folding, assembly and peptide loading of class I MHC	6/118	25/10619	2.46963E-07	7.53238E-06	5.50252E-06	3134/3133/3107/3106/6891/6890	6
Cluster 2	R-HSA-1834949	Cytosolic sensors of pathogen-associated DNA	7/118	63/10619	5.78686E-06	0.000162922	0.000119017	3428/4615/11277/7098/81844/3665/6737	7
Cluster 2	R-HSA-918233	TRAF3-dependent IRF activation pathway	4/118	14/10619	1.33089E-05	0.000347933	0.00025417	64135/23586/3665/7706	4
Cluster 2	R-HSA-983168	Antigen processing: Ubiquitination & Proteasome degradation	12/118	309/10619	0.000160979	0.003927889	0.002869381	7318/151636/55008/51191/5696/5698/6737/9246/140691/5699/57674/55905	12
Cluster 2	R-HSA-933543	NF-kB activation through FADD/RIP-1 pathway mediated by caspase-8 and -10	3/118	12/10619	0.000273524	0.005977088	0.004366351	64135/23586/7706	3
Cluster 2	R-HSA-933541	TRAF6 mediated IRF7 activation	4/118	29/10619	0.000277624	0.005977088	0.004366351	64135/23586/3665/7706	4
Cluster 2	R-HSA-1679131	Trafficking and processing of endosomal TLR	3/118	13/10619	0.000352705	0.006964535	0.005087696	1520/7098/81622	3
Cluster 2	R-HSA-196807	Nicotinate metabolism	4/118	31/10619	0.000361547	0.006964535	0.005087696	83666/54625/952/84875	4
Cluster 2	R-HSA-5656169	Termination of translesion DNA synthesis	4/118	32/10619	0.000409675	0.007497044	0.005476702	9636/7318/9246/7706	4
Cluster 2	R-HSA-3134975	Regulation of innate immune responses to cytosolic DNA	3/118	15/10619	0.000552086	0.009622063	0.007029059	11277/81844/6737	3
Cluster 2	R-HSA-1834941	STING mediated induction of host immune responses	3/118	16/10619	0.000674001	0.011212926	0.008191208	3428/11277/6737	3
Cluster 2	R-HSA-109581	Apoptosis	8/118	176/10619	0.000754714	0.012009791	0.00877333	8837/8743/1832/5696/5698/840/5699/5366	8
Cluster 2	R-HSA-5357801	Programmed Cell Death	8/118	179/10619	0.000842832	0.012853185	0.009389442	8837/8743/1832/5696/5698/840/5699/5366	8
Cluster 2	R-HSA-110313	Translesion synthesis by Y family DNA polymerases bypasses lesions on DNA template	4/118	39/10619	0.000882611	0.012921431	0.009439297	9636/7318/9246/7706	4
Cluster 2	R-HSA-197264	Nicotinamide salvaging	3/118	19/10619	0.001138254	0.016023117	0.011705124	83666/54625/84875	3
Cluster 2	R-HSA-451927	Interleukin-2 family signaling	4/118	44/10619	0.001395859	0.018921639	0.013822537	6772/3600/3601/3965	4
Cluster 2	R-HSA-73893	DNA Damage Bypass	4/118	49/10619	0.002087756	0.026957844	0.019693104	9636/7318/9246/7706	4
Cluster 2	R-HSA-5260271	Diseases of Immune System	3/118	24/10619	0.002283315	0.026957844	0.019693104	4615/7098/81622	3
Cluster 2	R-HSA-5602358	Diseases associated with the TLR signaling cascade	3/118	24/10619	0.002283315	0.026957844	0.019693104	4615/7098/81622	3
Cluster 2	R-HSA-933542	TRAF6 mediated NF-kB activation	3/118	24/10619	0.002283315	0.026957844	0.019693104	64135/23586/7706	3
Cluster 2	R-HSA-912694	Regulation of IFNA signaling	3/118	26/10619	0.002886136	0.033010181	0.024114426	6772/6773/11274	3
Cluster 2	R-HSA-180585	Vif-mediated degradation of APOBEC3G	4/118	54/10619	0.00298648	0.033122783	0.024196684	5696/5698/5699/60489	4

Supplementary Table S4: List of primers used for qPCR (Supplementary Fig. S6).

Name	Sequence	Used for
agrC_qPCR_F	CATTCGCGTTGCATTTATTG	ATCC29213
agrC_qPCR_R	CCTAAACCACGACCTTCACC	ATCC29213
hlgA_qPCR_F	GAGGCAGTGGCTCATTCAAC	ATCC29213
hlgA_qPCR_R	CTTGACCATTCGGTGTAACG	ATCC29213
isdB_qPCR_F	GGACAATACCATGTCAGAATCG	ATCC29213
isdb_qPCR_R	ATGGTGTTGGTTTGCTAGGC	ATCC29213
srtB_qPCR_F	AGCGAGAACATCGACGTAAAG	ATCC29213
srtB_qPCR_R	CATCGTATTATCACCGACATGG	ATCC29213
gyrB_fwd3	GAAGCATTAGCTGGTTATGCAA	Ref ATCC29213
gyrB_rev3	CCACGTCCGTTATCCGTTAC	Ref ATCC29213
JUNB_qPCR_F	CACCTCCCGTTTACACCAAC	HUVEC
JUNB_qPCR_R	GGAGGTAGCTGATGGTGGTC	HUVEC
SMAD6_qPCR_F	ACCAACTCCCTCATCACTGC	HUVEC
SMAD6_qPCR_R	TGGTCGTACACCGCATAGAG	HUVEC
HLA-B_qPCR_F	ACACCCAGTTCGTGAGGTTC	HUVEC
HLA-B_qPCR_R	GTGTGTTCCGGTCCCAATAC	HUVEC
PSMB10_qPCR_F	CACTGAGCTCACCCACAGAG	HUVEC
PSMB10_qPCR_R	TCCAGGGTTAGTGGCTTCAC	HUVEC
B2M_qPCR_F	TCTCTCTTTCTGGCCTGGAG	Ref HUVEC
B2M_qPCR_R	AATGTCGGATGGATGAAACC	Ref HUVEC

Supplementary Information References

1. Scheutz, F. VirulenceFinder 2.0. https://cge.cbs.dtu.dk/services/VirulenceFinder/ (2019).
2. Jenul, C. & Horswill, A. R. Regulation of Staphylococcus aureus virulence. *Microbiol Spectr.* **6**(1), GPP3-0031-2018 (2019).
3. Laarman, A. J. *et al.* Staphylococcus aureus metalloprotease aureolysin cleaves complement C3 to mediate immune evasion. *J Immunol.* **186**(11):6445-53 (2011).
4. Thammavongsa, V., Kim, H. K., Missiakas, D., Schneewind, O. Staphylococcal manipulation of host immune responses. *Nat Rev Microbiol*. **13**(9):529-43 (2015).
5. Skaar, E. P., Humayun, M., Bae, T., DeBord, K. L., Schneewind, O. Iron-source preference of Staphylococcus aureus infections.*Science*. **305**(5690):1626-8 (2004).
6. Marr, J. C. *et al.* Characterization of novel type C staphylococcal enterotoxins: biological and evolutionary implications. *Infect Immun*. **61**(10):4254-62 (1993).
7. Clarke, S. R., Wiltshire, M. D. & Foster, S. J. IsdA of Staphylococcus aureus is a broad spectrum, iron-regulated adhesin. Mol Microbiol. **51**(5):1509-19 (2004).
8. Bradshaw, W. J. *et al.* Molecular features of the sortase enzyme family. *FEBS J.* **282**(11), 2097-2114 (2015).
